# Pan-cancer analysis reveals synergistic effects of CDK4/6i and PARPi combination treatment in RB-proficient and RB-deficient breast cancer cells

**DOI:** 10.1038/s41419-020-2408-1

**Published:** 2020-04-06

**Authors:** Songyu Li, Yixiang Zhang, Na Wang, Rong Guo, Qiaoling Liu, Changsheng Lv, Jinguang Wang, Lina Wang, Qing-kai Yang

**Affiliations:** 1 Department of Oncology, Institute of Cancer Stem Cell, Dalian Medical University, 9 Western Lvshun South Road, 116044 Dalian, Liaoning China; 2 Department of Thoracic Surgery, The First Affiliated Hospital of Dalian Medicine University, No. 222 Zhongshan Road, Dalian, 116000 Dalian, Liaoning China

**Keywords:** Cancer therapy, Drug development

## Abstract

DNA damage results in mutations and plays critical roles in cancer development, progression, and treatment. Targeting DNA damage response in cancers by inhibiting poly-(ADP-ribose) polymerases (PARPs) offers an important therapeutic strategy. However, the failure of PARP inhibitors to markedly benefit patients suggests the necessity for developing new strategies to improve their efficacy. Here, we show that the expression of cyclin-dependent kinase 4/6 (CDK4/6) complex members significantly correlates with mutations (as proxies of DNA damages), and that the combination of CDK4/6 and PARP inhibitors shows synergy in both RB-proficient and RB-deficient breast cancer cells. As PARPs constitute sensors of DNA damage and are broadly involved in multiple DNA repair pathways, we hypothesized that the combined inhibition of PARPs and DNA repair (or repair-related) pathways critical for cancer (DRPCC) should show synergy. To identify druggable candidate DRPCC(s), we analyzed the correlation between the genome-wide expression of individual genes and the mutations for 27 different cancer types, assessing 7146 exomes and over 1,500,000 somatic mutations. Pathway enrichment analyses of the top-ranked genes correlated with mutations indicated “cell cycle pathway” as the top candidate DRPCC. Additionally, among functional cell-cycle complexes, the CDK4/6 complex showed the most significant negative correlation with mutations, also suggesting that combined CDK4/6 and PARP inhibition might exhibit synergy. Furthermore, combination treatment showed synergy in not only RB-proficient but also RB-deficient breast cancer cells in a reactive oxygen species-dependent manner. These findings suggest a potential therapeutic strategy to improve the efficacy of PARP and CDK4/6 inhibitors in cancer treatment.

## Introduction

DNA damage results in mutations and plays critical roles in the development, progression, and treatment of nearly all cancers^1–5^. Cells employ multiple kinds of DNA repair mechanisms to repair DNA damage. However, compared with normal cells, cancer cells frequently harbor decreased DNA repair and/or repair-related pathways^1–5^. In some cases, cancer cells even enhance mutagenic pathways to drive oncogenesis^2^. Consequently, cancer cells are often more reliant on a subset of DNA repair or repair-related pathway(s) critical for cancer (DRPCCs) to survive DNA damage. As important members of DRPCCs, poly (ADP-ribose) polymerases (PARPs) are best known for their critical roles in DNA repair^2,6^. Blocking the catalytic activity of PARP(s) has been demonstrated to inhibit DNA repair, resulting in the accumulation of DNA damage^2,3,5,7^. Importantly, inhibition of PARPs by small-molecule compounds disrupts the ability of cancer cells to survive ongoing DNA damage and consequently results in cell cycle arrest and/or cell death. Currently, the U.S. Food and Drug Administration (FDA) has approved PARP inhibitors as monotherapy for the treatment of ovarian cancer^8^. Furthermore, PARP inhibitors have also shown encouraging clinical activity in breast^9–12^, prostate^9,10,13^, and other solid cancers^9,10^. Although targeting PARPs offers an important therapeutic strategy, failure of PARP inhibitors (PARPis) to markedly benefit patients suggests the necessity for developing new strategies to improve their efficacy. Recent studies have shown that disruptions of HR-related pathways, such as by BRCA^1,14^, IDH1/2^15^, and PTEN mutations^16^, can enhance the sensitivity of cancer cells to PARPis. However, the majority of cancers are HR-proficient. Therefore, there is a need for the development of strategies to block DRPCC(s) and subsequently sensitize cancer cells to PARPi using methods such as systems-level analysis.

**Fig. 1:**
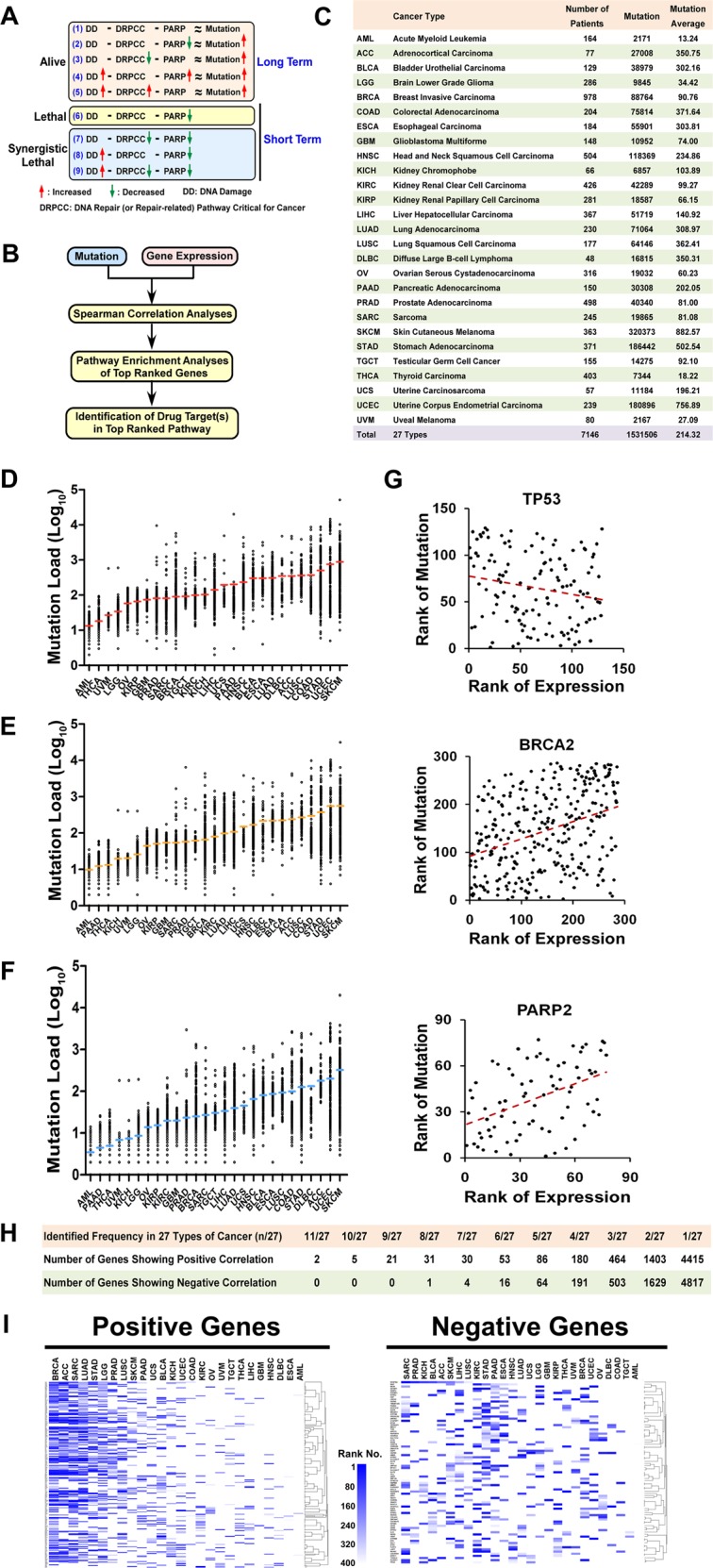
Pan-cancer analysis of the correlation between mutations and the genome-wide expression of individual genes. **a** Diagram showing the possible relationships among DNA damage, PARPs, DRPCC, and mutation. In detail, DNA damage and mutations are closely related (Formula 1). As both DRPCC(s) and PARP are involved in DNA repair, it is logical to suppose that cancer cells with decreased activity of DRPCC(s) and/or PARP should be biased toward acquiring more mutations (Formula 2 and 3). Moreover, cancer cells are often reliant on increased activity of PARP and/or DRPCC(s) to survive extra DNA damage from enhanced mutagenic pathway(s) and/or decreased DNA repair pathway(s) (Formula 4 and 5). Considering that PARPs are sensors of DNA damage and broadly involved in multiple DNA repair pathways, we reasoned that the combined inhibition of PARPs and DRPCC(s) should show synergy (Formula 7–9). **b** Schematic showing the pipeline used for the prediction of DRPCC(s) and drug target(s). **c** Summary statistics for the 27 different cancer types in this study. **d**, **f** All mutations (**d**), missense mutations (**e**), and sense mutations (**f**) across 27 cancer types. Each data point represents one tumor sample, and the *y-*axis is log_10_ transformed for better data visualization. Red, orange, or blue horizontal bars indicate the average mutation load. The *z*-scores for mRNA expression were calculated for each sample by comparing them RNA expression of a gene to the distribution in a reference population that represents typical expression for the gene. The returned value (*z*-score) indicates the number of standard deviations away from the mean of expression in the reference population. **g** Scatter plots showing the correlation between the ranks of mutation load and gene expression. Each data point represents one tumor sample. Data of TP53, BRCA2, and PAPR2 are from BLCA, LGG, and ACC, respectively. The red dashed line is the best fit for visualization. **h** Summary statistics for positive and negative genes across 27 cancer types. **i** Heatmap depicting the enrichment of positive (left) and negative (right) genes, respectively.

Owing to the large open-access efforts of The Cancer Genome Atlas (TCGA), it is now possible to explore the correlation between DNA damage (using mutations as proxies) and the genome-wide expression of individual genes in a pan-cancer cohort^17^. The majority of patients included in the TCGA database are accompanied by data regarding both mutations and genome-wide expression of individual genes^17^. Recent studies have demonstrated that there is a gene-specific correlation between messenger RNA (mRNA) and protein levels in human cells^18–20^. Additionally, the closely relativity between DNA damage and mutation allows quantitative analyses of mutation to be taken as a proxy of DNA damage (Fig. 1a)^2,4,5^. Considering that PARPs are broadly involved in multiple DNA repair pathways, we reasoned that the combined inhibition of PARPs and DRPCC(s) should show synergy (Fig. 1a). Moreover, the potential exists that systematic analyses of the pan-cancer cohort may identify candidate DRPCC(s) for inhibition to improve the efficacy of PARPis.

Additionly, cyclin-dependent kinase 4 (CDK4), cyclin-dependent kinase (CDK6), and D-type cyclins (D1–3) are components of the core cell cycle complex, which drives cell proliferation^21,22^. As a result of CDK4/6 activation, cancer cells are driven toward progression into the DNA synthetic (S) phase of the cell cycle^21,22^. Furthermore, the phosphorylation of RB by CDK4/6 has been described to be essential for the entry into S phase from G1^21,22^. Accordingly, inhibition of cyclin D-CDK4/6 kinase in RB-proficient cancer cells causes cell cycle arrest and/or senescence^23,24^, whereas, RB-deficient cancer cells do not halt their proliferation upon CDK4/6 inhibition^21,23,25^. Therefore, a large fraction of patients with RB-deficient cancers may not benefit from a CDK4/6 inhibitor (CDK4/6i), suggesting the necessity for developing new strategies to improve the efficacy of CDK4/6i.

In this study, we analyzed the correlation between individual gene expression and mutation in TCGA pan-cancer cohort, and identified the CDK4/6 complex as the top candidate DRPCC (Fig. 1b). The combination of CDK4/6i and PARPi showed synergy in not only RB-proficient but also in RB-deficient breast cancer cells in a reactive oxygen species (ROS)-dependent manner. These findings suggest a potential therapeutic strategy to improve the efficacy of PARPi and CDK4/6i in cancer treatment.

## Materials and methods

### Sample preparation

Samples from patients with cancer from TCGA used for mRNA expression and mutation analysis were collected through The cBio Cancer Genomics Portal (http://cbioportal.org) on 16 March, 2016 (Supplementary Table S1); the information regarding gender, age, and other clinical information can be found at http://cbioportal.org. Only samples with available mutation and transcriptomic data at that time point were used in this study.

### Cell culture

All cell lines were obtained from the American Type Tissue Collection. HeLa, Hs578T, and SKOV3 cells were maintained in DMEM containing 10% FBS and 1% penicillin–streptomycin at 37 °C under a humidified atmosphere of 5% CO_2_. MDA-MB231, MDA-MB468, and MDA-MB436 cells were maintained in L15 containing 10% FBS and 1% penicillin–streptomycin. MCF7 was maintained in EMEM containing 0.01 mg/ml human recombinant insulin, 10% FBS, and 1% penicillin–streptomycin. BT549 and HCC1937 cells were maintained in RPMI 1640 containing 10% fetal bovine serum (FBS) and 1% penicillin–streptomycin.

### Reagents

H2DCFDA (Cat. number: HY-D0940), BrdU (Cat. number: HY-15910), palbociclib (Cat. number: HY-50767), ribociclib (Cat. number: HY-15777), niraparib (Cat. number: HY-10619), and olaparib (Cat. number: HY-10162) were from MedChem Express. Anti-phospho-γ-H2AX (Ser139) (Cat. number: 05-636) and FITC-conjugated anti-BrdU (Cat. number: MAB3262F) antibodies were from Millipore. Anti-Rb (ab181616), Anti-phospho-Rb (Ser780) (ab173289), Anti-histone H3 (ab1791), and Anti- PARP1 (ab191217) were from Abcam.

### Immunofluorescence

Immunofluorescence was carried out as described in our previous study^26^.

### Comet assays

Comet assays were performed as described in a previous study^27^. Briefly, cells were trypsinized, washed with phosphate-buffered saline (PBS), and suspended in agarose (Trevigen). Neutral electrophoresis was conducted at 21 V for 1 h in the CometAssay Electrophoresis System (BioRad). Data were collected with an Olympus microscope and analyzed using OpenComet software^28^. Data are shown as the means ± SEM for three biological replicates with >100 cells analyzed per replicate. Statistical analysis was performed using the student’s *t*-test.

### Colony-forming assay

The colony-forming assay was carried out as described in a previous study^29^. Briefly, cells were seeded in 6-well plates and treated with single or combined drugs. Then, cells were re-fed every 2 days with fresh medium containing inhibitors until the colonies were noticeably formed. Resultant cells were washed in phosphate-buffered saline (PBS), stained with 0.05% crystal violet for 30 min, and counted.

### Determination of short-term cytotoxicity

Determination of short-term cytotoxicity was carried out as described in a previous study^29^. Briefly, 1 × 10^3^ cells/well were cultured in 96-well microplatesand treated every 2 days with various concentrations of inhibitors alone or in combination at a constant ratio (1:4 for niraparib:palbociclib, 1:2 for olaparib:palbociclib, 1:4 for niraparib: ribociclib, and 1:2 for olaparib: ribociclib). The concentrations of inhibitors ranged from 10 to 160 nM for niraparib, 20 to 320 nM for olaparib, 40 to 640 nM for palbociclib and 40 to 640 nM for ribociclib. After 7 days, MTT assay was carried out to determine cell survival. Combination indexes (CIs) were calculated using the formula: CI = (*E*_A_ + *E*_B_)/*E*_AB_, where *E*_A_ is the effect of drug A; *E*_B_ is the effect of drug B; and *E*_AB_ is the effect of the combination of drug A and drug B. CI indicates a synergistic (CI < 1), antagonistic (CI > 1), or similar (CI = 1) effect.

### BrdU incorporation analysis

BrdU incorporation analysis was carried out as described in previous studies^26,30,31^. Exponentially growing cells were pulsed with BrdU (Thermo Fisher Scientific) for 1 h. Then, cells were trypsinized and fixed in 75% ice-cold ethanol. This was followed by incubation with FITC-conjugated anti-BrdU antibody, propidium iodide (0.2 μg/μl), and RNase A. Resultant samples were analyzed by FACSC6 (Accuri) using CFlow software. All experiments were performed in triplicate from a minimum of three independent experiments.

### Intracellular ROS detection

Intracellular ROS detection was carried out as described in a previous study^32^. Briefly, the intracellular ROS were measured through the detection of dichlorodihydrofluorescein, the cleavage product of H2DCFDA by ROS^33^. Cells treated with or without CDK4/6i and/or PARPi were washed with PBS and incubated with 5 μM H2DCFDA for 30 min. Then, cells were dissociated with accutase after 30 min culture in phenol red-free medium. The fluorescent signals from cells were analyzed by FACS (BD Calibur; excitation/emission = 488/530 nm).

### Liquid chromatography/tandem mass spectrometry (LC-MS/MS) analysis of 8-oxo-dG

LC-multiple reaction monitoring (MRM) MS/MS analysis of 8-oxo-dG was carried out as described in our previous studies^34,35^ with a minor modification. Briefly, the digested DNA was separated by reverse phase ultra-performance liquid chromatography on a C18 column using the mobile phase of 90% water (containing 0.1% formic acid) and 10.0% methanol at a flow rate of 0.4 ml/min. Mass spectrometric detection was achieved by an API 5500 triple quadrupole (ABSciex) with an electrospray ionization source. MRM mode was used for the LC-MS/MS analysis. 8-oxo-dG and deoxyguanosine (dG) was detected in the form of the mono-nucleoside, *m*/*z* 284.0 to 168.0 (collision energy (CE) 24 V; declustering potential (DP) 91 V) for 8-oxo-dG; *m*/*z* 268.0 to 152.0 (CE 18 V; DP 60 V) for dG. Linearity in ionization efficiencies was verified by analyzing dilution series of authentic standards. External calibration curves for 8-oxo-dG and dG were used to create standard curves for subsequent normalization and quantification. The concentration of 8-oxodG or dG was calibrated by standard curve.

### Immunoblotting

To prepare whole-cell lysates, cells were lysed with RIPA lysis buffer. After thorough mixing and incubation at 4 °C for 30 min, lysates were centrifuged and supernatants were collected. To prepare chromatin-bound subcellular fraction, cells were collected and fractionated using a Subcellular Protein Fractionation Kit from Thermo Scientific (78840) following the manufacturer’s instructions. Immunoblotting was carried out as described in our previous study^26^.

### Xenografts

The following animal-handling procedures were approved by the Animal Care and Use Committee of Dalian Medical University. Xenograft models were carried out as previously described^26^. Briefly, 1 × 10^7^ cultured MDA-MB-231 or MDA-MB-468 cells were suspended and injected subcutaneously into the both left and right flank of 6-week-old female nude mice. After 7 days, these tumor-bearing mice were randomized into four groups (eight mice per group, *n* = 8). Then, mice were treated by oral gavage twice a day with vehicle, niraparib (4 mg/kg), palbociclib (16 mg/kg), or niraparib (4 mg/kg), and palbociclib (16 mg/kg) combination. Mice were observed daily and weighed 4 days per week. Tumor size was measured twice a week by caliper and tumor volume was calculated using the formula: 0.52 × *L* × *W*^2^, where *L* is the longest diameter and *W* is the shortest diameter. Mice were euthanized when tumors reached 1200 mm^3^ or showed necrosis. The correlation model for the repeated measures was spatial power. Treated groups are compared to the control group at each time point and measured at least twice each time.

### Mice sample preparation and HPLC–MS/MS conditions

Mice were treated by oral gavage for 6 h with vehicle, niraparib (4 mg/kg), palbociclib (16 mg/kg), or niraparib (4 mg/kg), and palbociclib (16 mg/kg) combination. Moreover, then the whole blood was collected into a labeled sodium heparin sprayed plastic tube. Five-hundred microliters of whole blood was collected and kept on ice for 2 h. The samples were subjected to centrifuge for 5 min at 4000 rpm. The supernatant was transferred to clean eppen-dorf tubes before evaporating to dryness (at 40 °C) under a gentle stream of nitrogen. Dry extracts were reconstituted using 100 μl of 80% methanol. The samples were subjected to HPLC–MS/MS analysis of Niraparib (Nir) and Palbociclib (Pal). Quantification of analytes was achieved on an HPLC system (Waters, Milford, MA, USA) coupled to an API 5500 triple quadrupole (ABSciex, Framingham, MA, USA) operating in positive electrospray ionization mode. The chromatographic separation was performed at 25 °C with the use of an ACQUITY UPLC BEH C18 column (2.1 × 100 mm, 1.7 μm); The mobile phase system consisting of 0.1% formic acid (A) and methanol (B) was applied at a flow rate of 0.4 ml/min. The following conditions were used: 0.0–1.0 min (1% B), 1.0–3.0 min (1–25% B), 3.0–4.0 min (25% B), and 4.0–5.0 min (1% B) were used. To minimize potential salt and other contaminants in the electrospray ionization (ESI) source, a time segment was set to direct the first 0.5 min of column elute to waste. For mass spectrometry detection, the multiple reaction monitoring (MRM) was implemented using the following mass transitions: 321.0/304.1 (Niraparib) and 448.0/141.1 (Palbociclib).

### Statistical analysis

*P*-values were calculated using a student’s *t*-test, Spearman correlation analysis, or chi-square test as noted. *p-*values < 0.05 were considered statistically significant.

## Results

### Pan-cancer analysis of the correlation between mutations and the genome-wide expression of individual genes

To identify the genes correlated with DNA damage, we performed a pan-cancer analysis of the correlation between mutations and the genome-wide expression of individual genes. We retrieved RNA-Seq *z*-score data together with exomic mutation data corresponding to the 27 different human cancers that are available in TCGA through The cBio Cancer Genomics Portal. As a result, data were collected from 7146 individuals out of over 8000 cancer patients included in the TCGA project (Fig. 1c). *z*-scores were determined from the expression values as previously described^36,37^, and used to enable the quantitative comparisons between RNA-Seq data. Unless specifically noted, mutations include base-substitution mutations, insertions, and deletions (Fig. 1d and Supplementary Table S1). To evaluate the impact of selective stress, missense (Fig. 1e and Supplementary Table S2) and sense (Fig. 1f and Supplementary Table S3) mutations were also used to carry out the correlation analyses with gene expression.

Spearman’s rank analyses were carried out to assess the correlation between the expression of individual genes and mutations. For example, the expression of TP53 showed a negative correlation with the mutations in BLCA (*p*-value = 0.0121, *r* = −0.222 by Spearman’s correlation) (Fig. 1g). Conversely, BRCA2 (*p* = 2.61E − 10, *r* = 0.362) and PARP2 (*p* = 4.38E − 5, *r* = 0.448) showed positive correlations with the mutation loads in LGG and ACC, respectively (Fig. 1g). However, we noticed that the different sample sizes of different cancer types affected the calculated correlation value of a gene. Therefore, to enable quantitative comparisons between different cancer types, we used the rank number of a gene as a threshold, instead of the *r* correlation value. The top 400 genes showing positive or negative correlation with mutation in each type of cancer are shown in the Supplementary Material. Notably, genes showing positive correlation with mutation (positive genes) were biased toward enrichment in several types of cancers, compared with genes showing negative correlation with mutation (negative genes) (Fig. 1h). A total of 142 positive genes were ranked as among the top 400 in at least six types of cancers, whereas only 21 negative genes were ranked accordingly (Fig. 1h). Furthermore, BRCA, LUAD, LUSC, LGG, STAD, PRAD, SARC, and ACC shared multiple positive genes (Fig. 1i). In contrast, the positive genes of AML and ESCA showed little similarity with the positive genes of other cancers (Fig. 1i).

### Pathway enrichment analysis demonstrates that the “cell cycle pathway” shows the most significant positive correlation with mutations

**Fig. 2:**
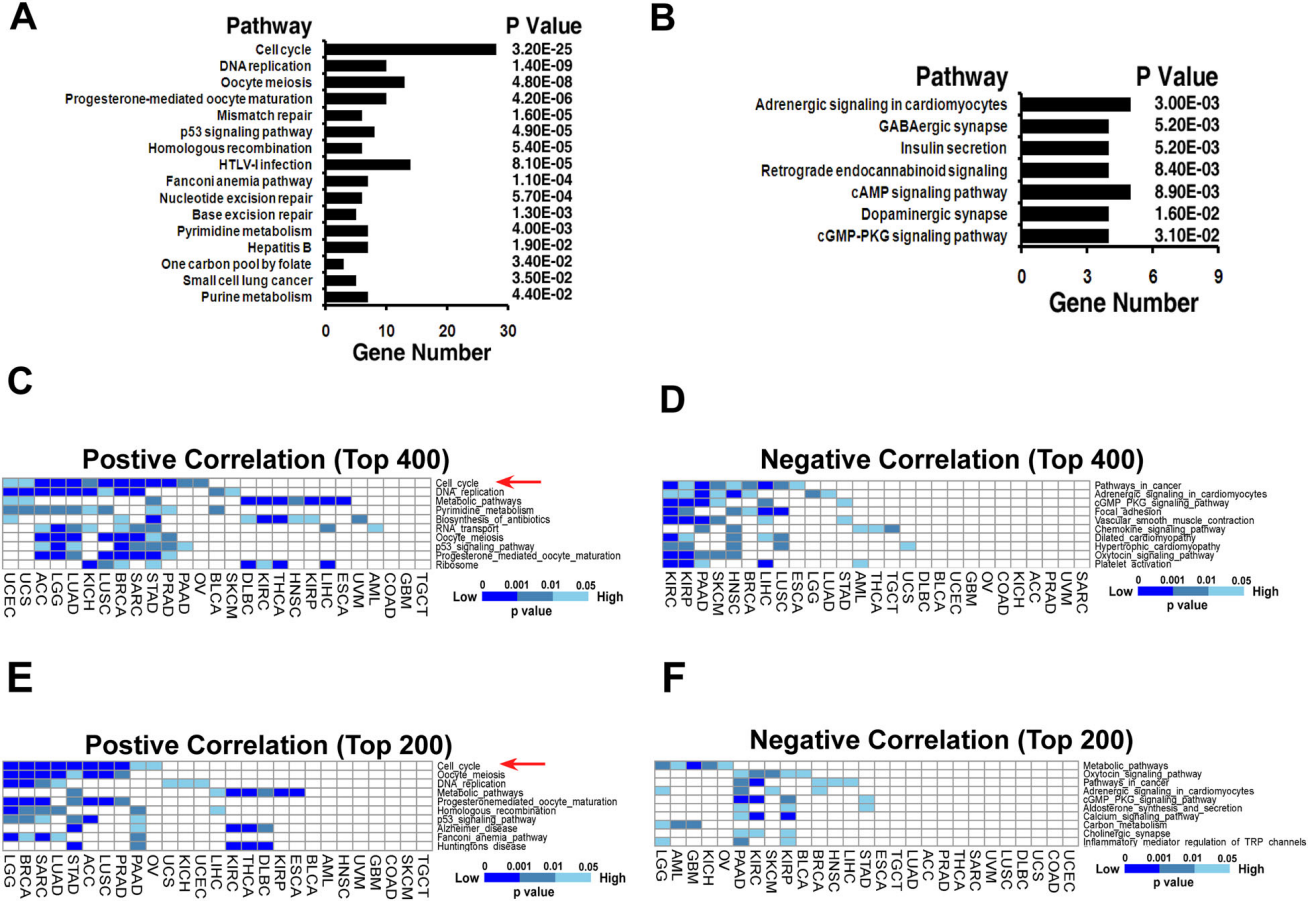
Cell cycle pathway shows the most significant positive correlation with mutation. **a**, **b** Top-ranked pathways (*p* < 0.05) of positive (**a**) and negative (**b**) genes ranked as among the top 400 in at least 5 types of cancers. **c**, **d** Heatmap depicting the top ten enriched pathways of the top 400 positive (**c**) and negative (**d**) genes correlated with all mutations across 27 cancer types. **e**, **f** Heatmap depicting the enriched pathways of the top 200 positive (**e**) and negative (**f**) genes correlated with all mutations.

To identify druggable DRPCC(s), we performed pathway enrichment analysis using the DAVID Functional Annotation Tool with *p* < 0.05. The positive or negative genes ranked as among the top 400 in at least five types of cancers (Supplementary Table S4) were used for this analysis. As shown in Fig. 2a, the top-ranking positive genes were significantly enriched in “cell cycle” (*p* = 3.2E − 25), “DNA replication”, and “oocyte meiosis” pathways, whereas the top-ranking negative genes (Supplementary Table S5) were enriched in “adrenergic signaling in cardiomyocytes” (*p* = 3.0E − 03), “GABAergic synapse”, and “insulin secretion” pathways (Fig. 2b).

Next, we carried out pathway enrichment analyses of the top positive and negative genes in every single type of cancer. We first analyzed the top 400 positive (Fig. 2c and Supplementary Table S6) and negative (Fig. 2d and Supplementary Table S7) genes for all mutations in 27 types of cancers. As shown in Fig. 2c, the “cell cycle pathway” showed the most significant ability to enrich positive genes in indicated cancers. To determine the impact of gene number, we analyzed the top 200 positive (Fig. 2e and Supplementary Table S8) and negative (Fig. 2f and Supplementary Table S9) genes in multi types of cancers. Consistent with the observation of the top 400 genes, the “cell cycle pathway” still showed the most notable ability to enrich positive genes in those cancers. To further determine the impact of selective stress, we analyzed the top 400 genes correlated with missense mutations (Fig. S1A, B and Supplementary Tables S10, 11) or sense mutations (Fig. S1C, D and Supplementary Table S12, 13). Finally, to determine the impact of gene number, we also analyzed the top 200 genes correlated with missense mutations (Fig. S1E, F and Supplementary Tables S14, 15) or sense mutations (Fig. S1G, H and Supplementary Tables S16, 17). In line with the results of all mutations, the “cell cycle pathway” always showed the most significant positive correlation with missense or sense mutations. We then identified the cell cycle pathway containing 124 genes (Supplementary Table S18) as a candidate DRPCC for potential combination with PARPis.

### CDK4/6 complex among the cell cycle pathway complexes shows the most significant negative correlation with mutations

**Fig. 3:**
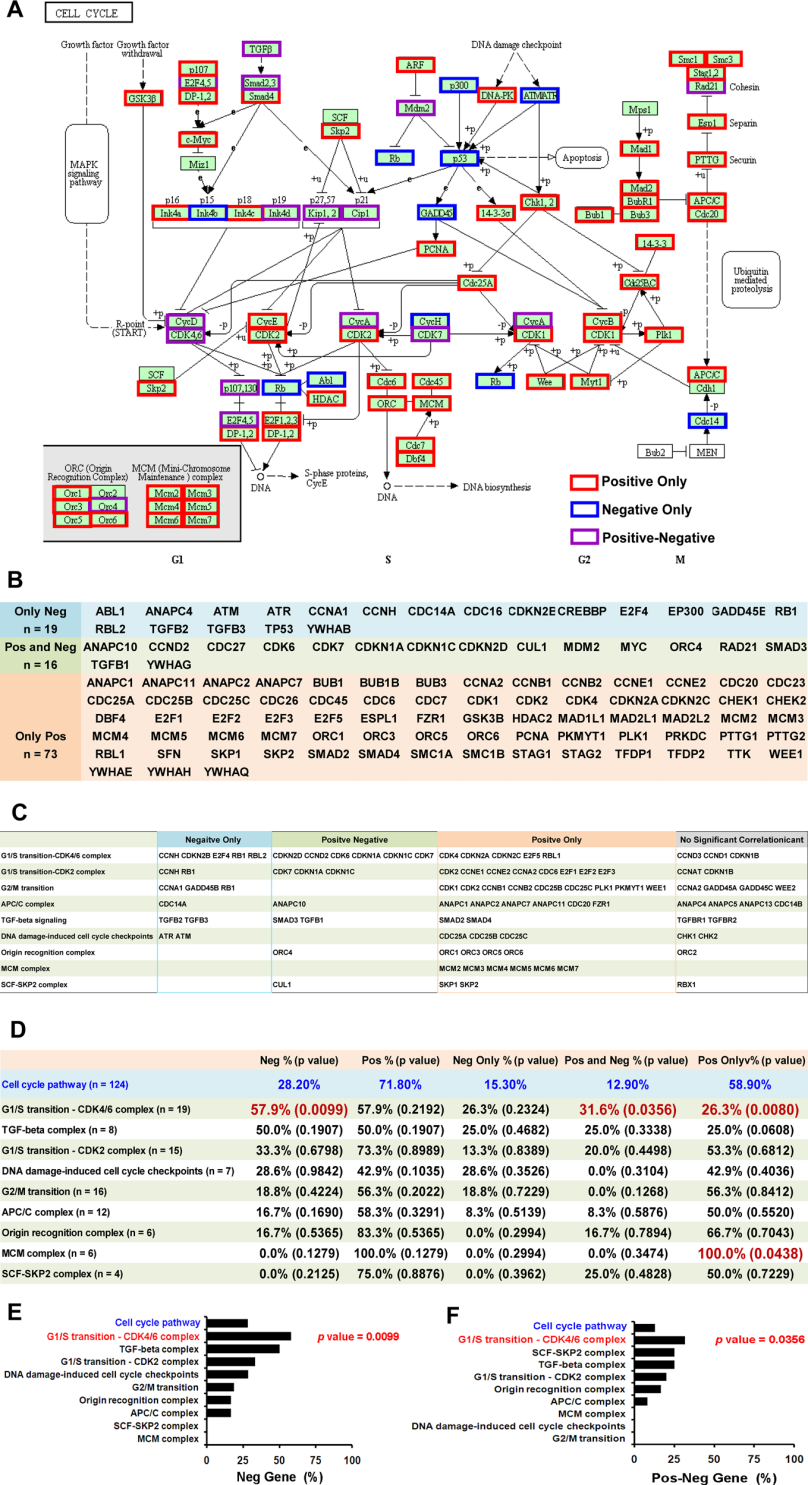
CDK4/6 complex shows the most significant negative correlation with mutations. **a** Diagram of the “cell cycle pathway” showing positive only, negative only, and positive-negative pathway genes. The schematic diagram of the “cell cycle pathway” is from DIVD (david.ncifcrf.gov). Red rectangle: positive-only genes showing only positive correlation with mutations across 27 cancer types; blue rectangle: negative only genes showing only negative correlation with mutations; purple rectangle: positive-negative genes showing positive correlation in at least one cancer type, and negative correlation in at least one cancer type. **b** Summary for positive only, negative only, and positive-negative genes in the “cell cycle pathway”. **c** Summary for nine functional complexes of the “cell cycle pathway”. **d** Summary statistics for the enrichment analysis of nine functional complexes. Chi-square test was used to calculate the enrichment significance (*p*-value) between the individual functional complex and the “cell cycle pathway” (blue). Significant enrichments (*p*-value < 0.05) are highlighted (red). **e** Negative gene enrichment (%) of every complex. **f** Negative-positive gene enrichment (%) of every complex.

Considering that the cell cycle pathway contains 124 genes and nine functional complexes, we performed gene enrichment analyses to identify the functional complex on which cancer cells are reliant to survival DNA damage. Among the 124 genes, 19 genes, including TP53, RB1, ATM, and ATR, showed only negative correlation with mutations in multiple types of cancers (Fig. 3a, b). In comparison, 73 genes including MCM2 through MCM7 showed only positive correlation (Fig. 3a, b). Additionally, 16 genes showed not only positive correlation in at least one cancer type but also negative correlation in at least one other cancer type (positive-negative genes) (Fig. 3a, b). Based on the well-studied roles of TP53, RB1, ATM, and ATR in the reduction of DNA damage, we supposed that the negative genes might contribute to the decrease of DNA damage. We further reasoned that the percentage of negative genes in the individual complex at least partially reflected its ability to reduce DNA damage. We then carried out a chi-square test to calculate the *p*-values of enrichment against the cell cycle pathway. As shown in Fig. 3c, d, the positive-only genes were notably enriched in the mini chromosome maintenance (MCM) complex (percentage of positive-only genes = 100%, chi-square test *p* = 0.0438). While, the other eight complexes showed no significant ability to enrich the positive genes (*p* > 0.7) (Fig. 3d). Importantly, the negative genes (57.9%, *p* = 0.0099) (Fig. 3d, e), as well as the positive-negative genes (31.6%, *p* = 0.0356) (Fig. 3d, f) were enriched in the CDK4/6 complex. As the MCM complex showed enrichment only for positive genes, it appeared that this complex might serve as a potential resource of DNA damage. Conversely, it was highly likely that cancer cells depended on the CDK4/6 complex to survive DNA damage. Therefore, we hypothesized that a combination of CDK4/6i and PARPi might show synergistic effects.

### Combined inhibition of CDK4/6 and PARP shows synergy in RB-proficient cells

**Fig. 4:**
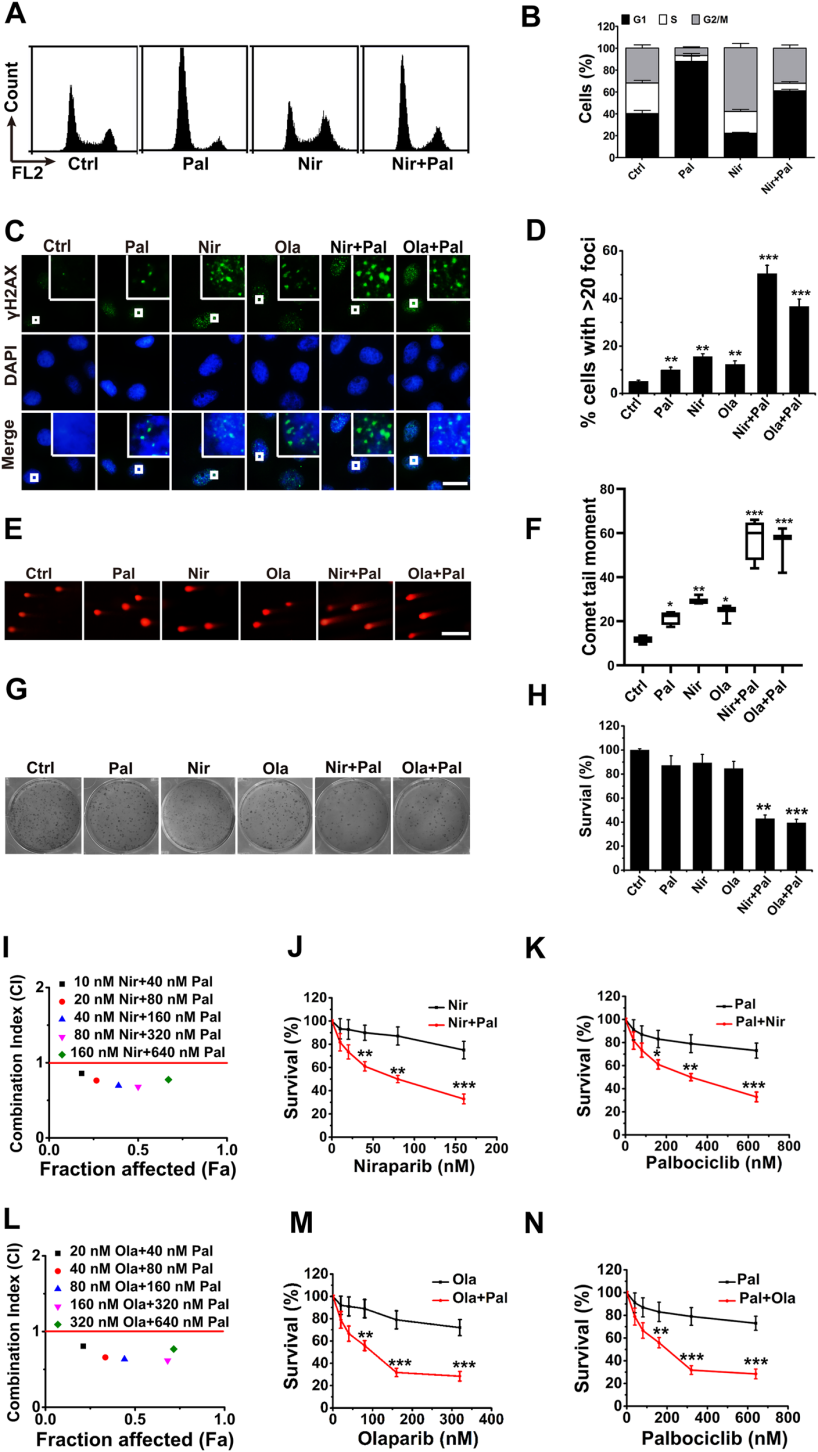
Combined inhibition of CDK4/6 and PARP shows synergy in RB-proficient cells. **a** Representative propidium iodide (PI) staining of MDA-MB-231 cells treated with 200 nM palbociclib, 50 nM niraparib, or the combination of palbociclib and niraparib (concurrent) for 24 h. **b** Percentages of the different cell cycle phase (G1, S, and G2/M) in the cells as described in **a**. **c** Representative fluorescent images of γ-H2AX foci in MDA-MB-231 cells treated with 50 nM niraparib, 100 nM olaparib, 200 nM palbociclib, or the combination of inhibitors as noted for 24 h. Scale bar = 10 μm. **d** Percentage of cells with >20 γ-H2AX foci in the MDA-MB-231 cells described in **c**. *p*-values between drug treatments vs. control were calculated by the *t*-test. **e** Representative images of neutral comet analyses of the MDA-MB-231 cells treated as described in **c**. Scale bar = 50 μm. **f** Comet tail moment of the MDA-MB-231 cells treated as described in **c**. *p*-values between drug treatments vs. control were calculated by the *t*-test. **g** Representative crystal violet staining of MDA-MB-231 cells treated every 2 days with 50 nM niraparib, 100 nM olaparib, 200 nM palbociclib, or the combination of inhibitors until the colonies were notably formed. **h** Clonogenic survival percentage of MDA-MB-231 cells as described in **g**. *p*-values between drug treatments vs. control were calculated by the *t*-test. **i** Combination indexes (CIs) between palbociclib and niraparib at different concentrations. MDA-MB-231 cells were treated every 2 days with niraparib (10–160 nM), palbociclib (40–640 nM), or the combination of niraparib and palbociclib at a constant ratio (niraparib:palbociclib = 1:4) for 7 days. MTT assay was carried out to determine cell survival. CIs were calculated using the formula: CI = (*E*_A_ + *E*_B_)/*E*_AB_, where *E*_A_ is the effect of drug A; *E*_B_ is the effect of drug B; and E_AB_ is the effect of the combination of drug A and drug B. Fa is the fraction affected by the combination of drug A and drug B. **j**, **k** Cell survival percentage of MDA-MB-231 cells treated as described in **i**. *p*-values between single treatment and combination treatments were calculated by the *t*-test. **l**, **n**, CI (**l**) and cell survival (**m**, **n**) analyses of MDA-MB-231 cells treated every 2 days with olaparib (20–320 nM), palbociclib (40–640 nM), or the combination of olaparib and palbociclib at a constant ratio (olaparib:palbociclib = 1: 2) for 7 days. Data are from three independent experiments, mean ± SEM (represented by error bars). *p < 0.05, **p < 0.01, ***p < 0.001, by the student’s *t*-test.

Previous studies have demonstrated that HR DNA repair is highly suppressed in G1/S phase cells to ensure that mitotic recombination only occurs between sister chromatids^38–40^. Considering that inhibition of CDK4/6 is supposed to cause G1/S cell cycle arrest in RB-proficient cells^23,24^, we first determined whether the combination of CDK4/6i and PARPi synergistically enhanced DNA damage in such cells. Several RB-proficient cells were treated with CDK4/6i (palbociclib and ribociclib) and/or PARPi (niraparib and or olaparib). As expected, palbociclib treatment resulted in a prominent G1 to S cell cycle arrest, whereas niraparib treatment elicited a profound arrest of cells with a tetraploid DNA content (G2/M phase) (Fig. 4a, b). Moreover, the combination of palbociclib and niraparib led to a hybrid cell cycle distribution, wherein the majority of cells were distributed in G1 and G2/M phase (Fig. 4a, b). As a measure of functional activity of CDK4/6 the phosphorylation state of Rb was assessed using immunoblotting. CDK4/6i resulted in inhibition of Rb phosphorylation, while PARPi have no effect on Rb phosphorylation (Supplementary Fig. S2A). In addition, the combination of CDK4/6i and PARPi showed the similar inhibition level of pRB as treatment with CDK4/6i alone (Supplementary Fig. S2A). Further 5-bromo-2-deoxyuridine (BrdU) incorporation analyses also indicated that CDK4/6i and PARPi result in G1/S and G2/M cell cycle arrest, respectively (Supplementary Fig. S2B, G). We then assessed the DNA damage in RB-proficient cells treated with CDK4/6i, PARPi, or their combination. As shown in Fig. 4c, d, the combination of CDK4/6i and PARPi enhanced γ-H2AX foci in MDA-MB231 cells, with the number of γ-H2AX-positive MDA-MB231 cells containing over 20 foci being increased approximately fivefold by the drug combination, compared with that following palbociclib or niraparib treatment alone (Fig. 4d). Consistent with the observation in MDA-MB231 cells, the combination also enhanced γ-H2AX foci in the cells (Supplementary Fig. S2H–L). To further confirm the impact of the drug combination on DNA damage, we also carried out a neutral comet assay. In line with the results of γ-H2AX foci, the combination increased comet tail moments in MDA-MB231 (Fig. 4e, f) and other RB-proficient cells (Supplementary Fig. S2M–Q) by approximately fivefold. Moreover, trapping PARP1 on damaged DNA has recently been proposed as a mechanism accounting for the cytotoxicity of PARPi^41^. We examined chromatin-bound PARP1 using a cellular assay to measure PARP1 trapping on damaged DNA^41^. As a result, in RB-proficient cells, combinations of PARPi and CDK4/6i lead to enhanced PARP1 bound in chromatin compared with PARPi treatment alone (Supplementary Fig. S2R).

Next, we determined whether the combined inhibition of CDK4/6 and PARP showed synergistic effect in RB-proficient cells. A colony formation assay was first carried out to evaluate the long-term role of the drug combination. As shown in Fig. 4g, h, MDA-MB231 cells showed ~5–10-fold greater sensitivity to the combined drugs than to each single drug. Consistent with this observation, the combination also exhibited synergistic effect in the long-term inhibition of the cancer cells (Supplementary Fig. S3A, D). To evaluate the short-term cytotoxicity resulting from CDK4/6i and PARPi combination, we treated the cells with different doses of inhibitor(s) for 7 days and assessed the resultant cell survival using the MTT assay. As shown in Fig. 4i, combination indices (CIs) between palbociclib and niraparib at different concentrations ranged from 0.4 to 0.7, indicating a synergistic effect between these drugs in MDA-MB231 cells. The survival of MDA-MB231 cells treated with palbociclib and/or niraparib at different concentrations are shown in Fig. 4j, k. In line with these results, CI and cell survival analyses indicated that palbociclib enhanced the short-term sensitivity of MDA-MB231 cells to olaparib (Fig. 4l–n). Furthermore, another CDK4/6i ribociclib also enhance the sensitivity of those in MDA-MB-231 and SKOV3 cells (Fig. S3E–H). Moreover, CDK4/6i and PARPi combination treatment also showed synergistic effect in the short-term inhibition of MCF7, Hs578T, HeLa, and SKOV3 cells (Supplementary Fig. S3I–P). As HR DNA repair is highly suppressed in G1/S phase cells^38–40^, it is not surprising that the combined inhibition of CDK4/6 and PARP shows synergy in RB-proficient cells.

### Combined inhibition of CDK4/6 and PARP also synergy in RB-deficient cells as well

**Fig. 5:**
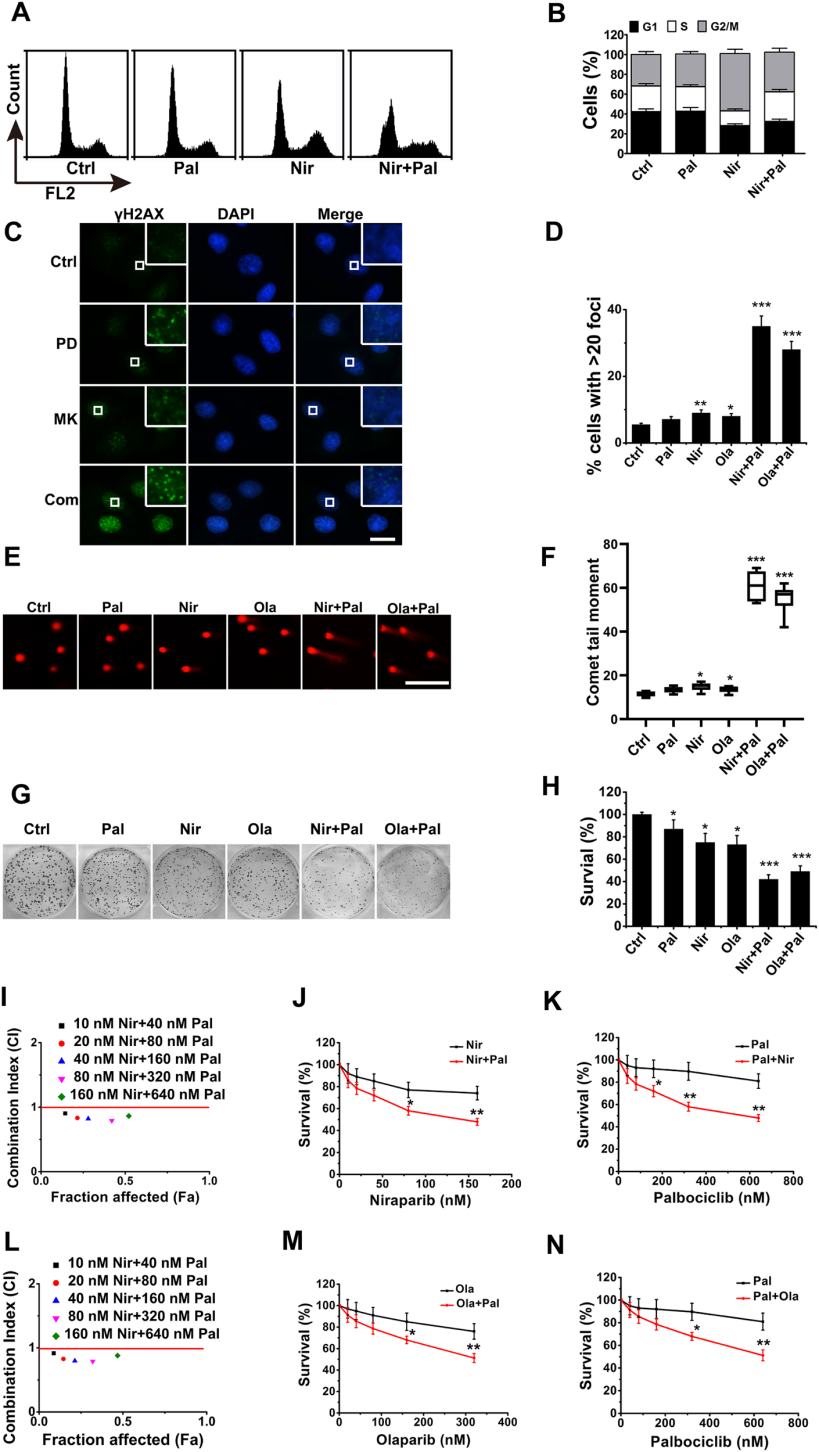
Combined inhibition of CDK4/6 and PARP also shows synergy in RB-deficient cells. **a** Representative flow cytometric analyses of MDA-MB-468 cells treated with 50 nM niraparib, 200 nM palbociclib, or the combination of niraparib and palbociclib for 24 h. **b** Percentages of the different cell cycle phase (G1, S, G2/M) in the cells described in **a**. **c**, **d** γ-H2AX foci in MDA-MB-468 cells treated with 50 nM niraparib, 100 nM olaparib, 200 nM palbociclib, or the combination of inhibitors as noted for 24 h. Scale bar = 10 μm (**c**). *p*-values between drug treatments vs. control were calculated by the *t-*test. **e**, **f** Representative images of neutral comet analyses and comet tail moment of the MDA-MB-468 cells treated as described in **c**. *p*-values between drug treatments vs. control were calculated by the *t*-test. Scale bar = 50 μm (**e**). **g**, **h** Representative crystal violet staining and clonogenic survival percentage of MDA-MB-468 cells treated as described in (**c**). *p*-values between drug treatments vs. control were calculated by the *t*-test. **i**–**k** Combination index (**i**) and cell survival (**j**, **k**) analyses of MDA-MB-468 cells treated every 2 days with niraparib (10–160 nM), palbociclib (40–640 nM), or the combination of niraparib and palbociclib at a constant ratio (niraparib: palbociclib = 1: 4) for 7 days. *p-*values between single treatment and combination treatments were calculated by the *t*-test. **l**–**n** Combination index (**l**) and cell survival (**m**, **n**) analyses of MDA-MB-468 cells treated every 2 days with olaparib (20–320 nM), palbociclib (40–640 nM), or the combination of olaparib and palbociclib at a constant ratio (olaparib:palbociclib = 1: 2) for 7 days. Experiments performed in triplicates; mean ± SEM (represented by error bars). *p < 0.05, **p < 0.01, ***p < 0.001.

Considering that the large fraction of patients with RB-deficient cancers may not benefit from CDK4/6i, we then determined whether the combined inhibition of CDK4/6 and PARP showed synergistic effect in RB-deficient cancer cells. The RB-deficient cell lines MDA-MB468, MDA-MB436, BT549, and HCC1937, as described in previous studies^31^, were used for this purpose. Unlike RB-proficient cells, RB-deficient cells by passed the G1/S cell cycle arrest effect stemming from CDK4/6 inhibition, which was consistent with previous report^31^ (Fig. 5a, b). In addition, the combination of CDK4/6i and PARPi showed similar cell cycle distribution as treatment with PARPi-alone (Fig. 5a, b). Subsequent BrdU incorporation analyses confirmed that RB-deficient cells showed no appreciable changes in BrdU incorporation in response to CDK4/6i (Supplementary Fig. S4A–E), while the combination of CDK4/6i and PARPi exhibited similar BrdU incorporation levels as treatment with PARPi-alone (Supplementary Fig. S4A–E). We then carried out γ-H2AX focus formation and neutral comet assays to evaluate the impacts of the combination of CDK4/6i and PARPi on DNA damage in RB-deficient cells. Notably, the number of γ-H2AX-positive cells were still increased ~5–10-fold by the drug combination in the RB-deficient cells (Fig. 5c, d and Supplementary Fig. S4F–H). Consistent with this observation, we found that the CDK4/6i and PARPi combination also led to an ~5-fold increase of comet tail moments in the RB-deficient cells (Fig. 5e, f and Supplementary Fig. S4I–K) as well. Moreover, the combination of CDK4/6i and PARPi also enhanced the bound of PARP1 in chromatin compared with PARPi treatment alone in the RB-deficient cells (Supplementary Fig. S4L).

Next, we determined whether the combined inhibition of CDK4/6 and PARP showed synergistic effect on the inhibition of RB-deficient cells. We first carried out a colony formation assay to evaluate the long-term role of CDK4/6i and PARPi combination. The assays demonstrated that the RB-deficient cells showed ~5–10-fold enhanced sensitivity to the combined drugs than to each single drug (Fig. 5g, h and Supplementary Fig. S5A–C). To evaluate the short-term cytotoxicity resulting from CDK4/6i and PARPi combination, we treated the RB-deficient cells with different doses of inhibitor(s) for 7 days. RB-deficient cells also showed greater sensitivity to combined drugs than to niraparib (Fig. 5i–k and Supplementary Fig. S5D, F, H, J, and L) or olaparib (Fig. 5l–n and Supplementary Fig. S5E, G, I, K, and M) alone. The CIs between CDK4/6i and PARPi at different concentrations ranged from 0.3 to 0.8, indicating a synergistic effect between these inhibitors in RB-deficient cells (Fig. 5i–n and Supplementary Fig. 5D–M).

### Inhibition of CDK4/6 shows synergistic effect with PARPi in RB-deficient cells through ROS

**Fig. 6:**
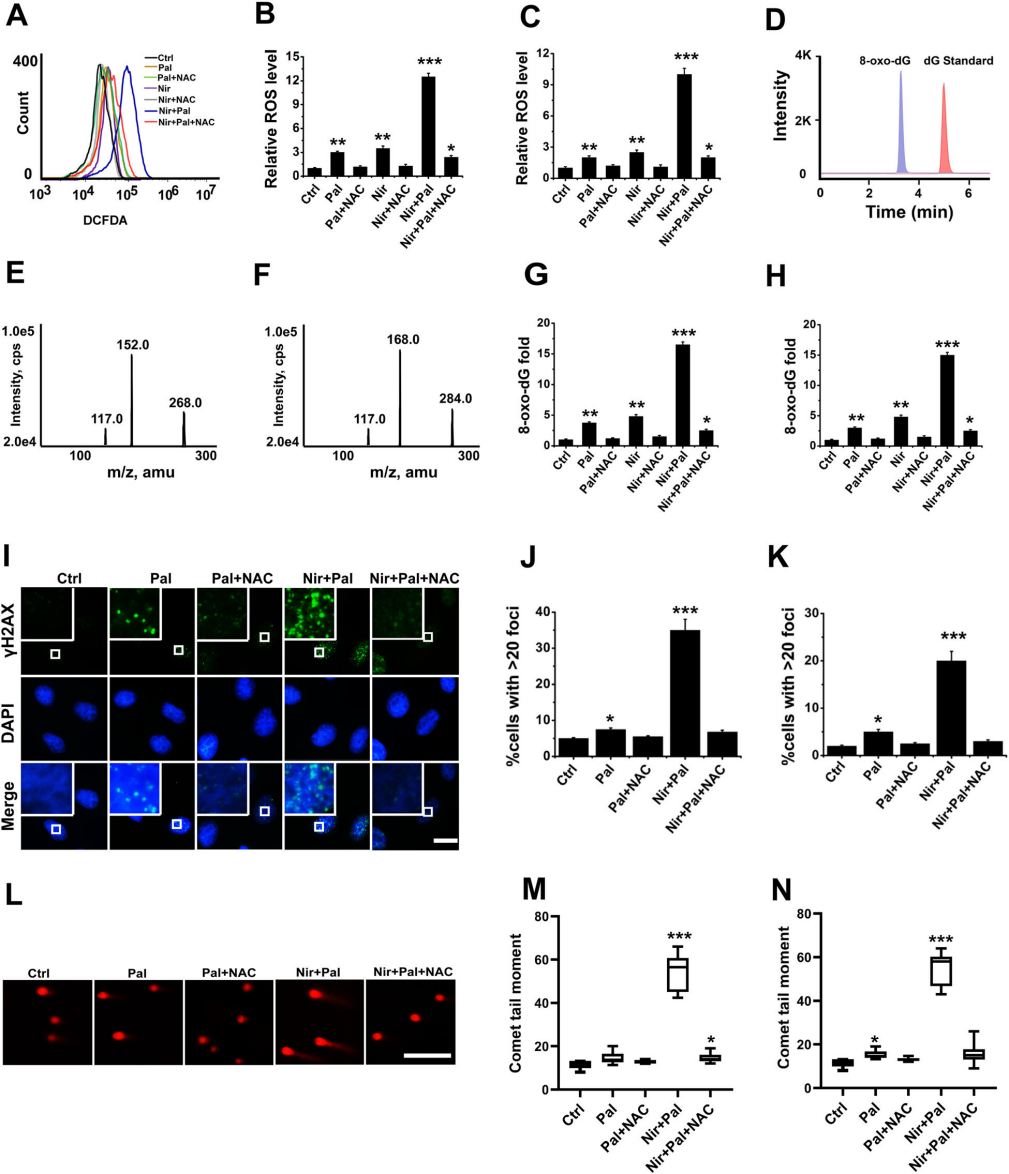
Combination of CDK4/6i and PARPi shows synergy to increase DNA damage in RB-deficient cancer cells through ROS. **a**–**c** ROS levels in MDA-MB-468 (**a**, **b**) and MDA-MB436 (**c**) cells treated with 50 nM niraparib, 200 nM palbociclib, 2.5 mM NAC, or the combination of drugs as noted for 24 h. ROS level of control cells mock-treated was taken as 1. **d** Retention time of dG and 8-oxo-dG in HPLC–MS/MS analysis. **e** Base ion mass transitions of dG (268.0 to 152.0) in HPLC–MS/MS analysis. **f** Base ion mass transitions of 8-oxo-dG (284.0 to 168.0). **g**, **h** 8-oxo-dG levels (8-oxo-dG/dG) of MDA-MB-468 (**g**) and MDA-MB436 (**h**) cells treated with 50 nM niraparib, 200 nM palbociclib, 2.5 mM NAC, or the combination of drugs as noted for 24 h. 8-oxo-dG levels of the cells treated with DMSO were taken as 1. **i**–**k** γ-H2AX analyses of MDA-MB-468 (**i**, **j**) and MDA-MB436 (**k**) cells treated with 50 nM niraparib, 200 nM palbociclib, 2.5 mM NAC, or the combination of drugs as noted for 24 h. Scale bar = 10 μm. **l**–**n** Comet analyses of MDA-MB-468 (**l**, **m**) and MDA-MB436 (**k**) cells treated with 50 nM niraparib, 200 nM palbociclib, 2.5 mM NAC, or the combination of drugs as noted for 24 h. Experiments in triplicate are represented, mean ± SEM (represented by error bars). *p < 0.05, **p < 0.01, ***p < 0.001, by the student’s *t*-test, drug treatments vs. control.

We then investigated the mechanism of the synergistic effects of CDK4/6i and PARPi combination in RB-deficient cells. As CDK4/6i enhanced γ-H2AX foci (a classic indicator of double strand breaks that frequently result from endogenous ROS) in RB-deficient cells, we reasoned that CDK4/6i might show synergistic effect with PARPi through ROS. To evaluate this hypothesis, we first measured the amount of intracellular ROS by detecting dichlorodihydrofluorescein (DCFDA), the cleavage product of 2, 7-dichlorodihydrofluorescein diacetate (carboxy-H2DCFDA) by ROS. DCFDA analyses indicated that palbociclib alone or the combination notably enhanced the ROS in the RB-deficient cells, whereas the antioxidant N-acetyl-cysteine (NAC) blocked the increase of ROS levels (Fig. 6a–c and Supplementary Fig. S6A, B). We then employed LC-MS/MS to assess the levels of 8-oxo-2’-deoxyguanosine (8-oxo-dG) (Fig. 6d), the most frequent oxidative product of DNA damage by ROS. The nucleosides were quantified by MS using the nucleoside to base ion mass transitions of 268.0 to 152.0 for dG (Fig. 6e), and 284.0 to 168.0 for 8-OXO-dG (Fig. 6f). LC-MS/MS analyses of 8-oxo-dG indicated that CDK4/6i enhanced the 8-OXO-dG levels, whereas NAC diminished it upon CDK4/6i treatment alone or the combination of CDK4/6i and PARPi (Fig. 6g, h and Supplementary Fig. S6E–N). Furthermore, NAC blocked the increase of γ-H2AX foci upon treatment with CDK4/6i alone or the combination of CDK4/6i and PARPi in the RB-deficient cells (Fig. 6i–k and Supplementary Fig. S6O–P). Consistent with these observations, neutral comet assays demonstrated that NAC diminished the increase of comet tail levels in response to drug treatments (Fig. 6l–n and Supplementary Fig. S6Q, R). It therefore appeared that the combined inhibition of CDK4/6 and PARP showed synergy to increase DNA damage in RB-deficient cancer cells through ROS.

**Fig. 7:**
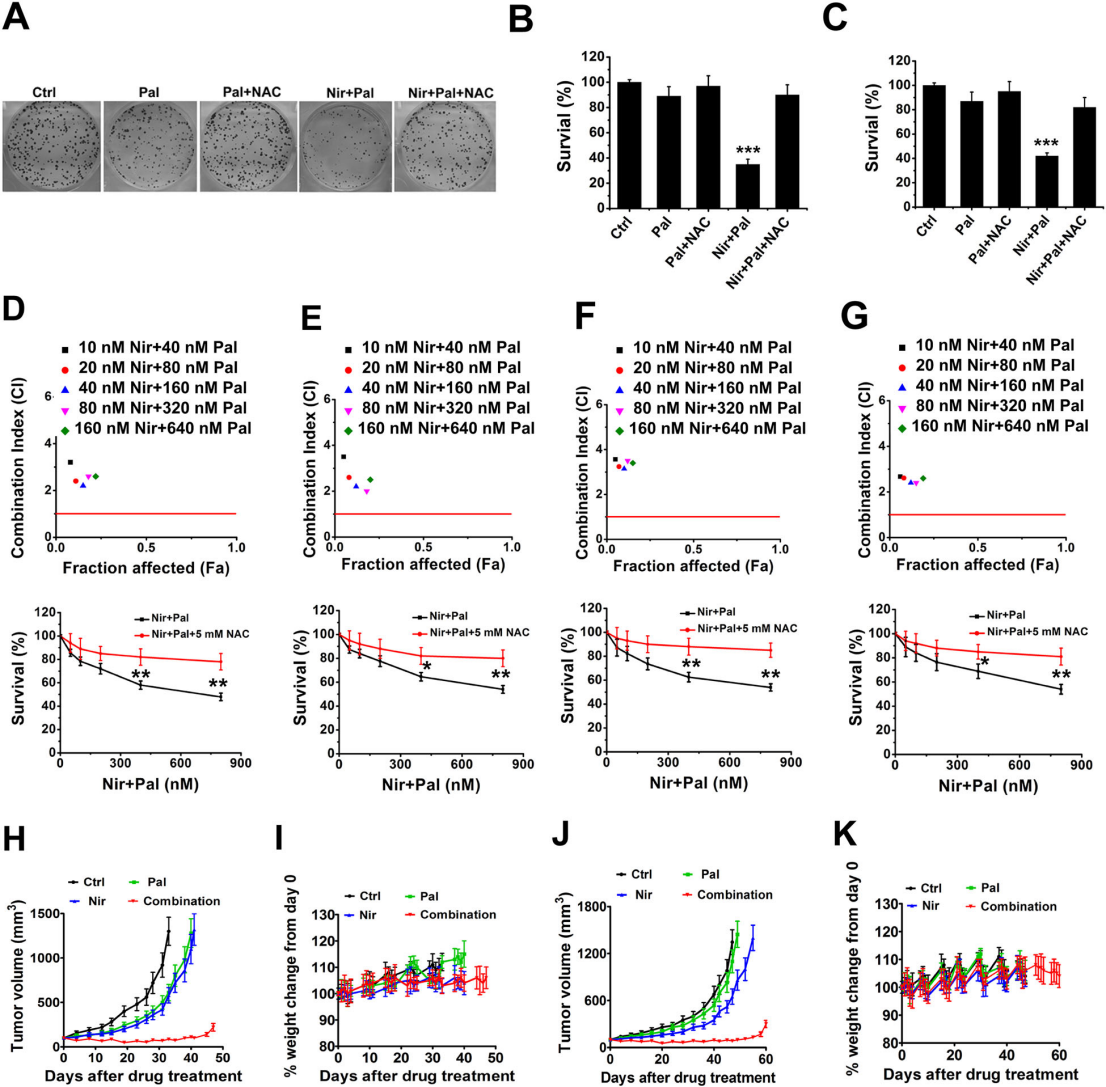
Combination of CDK4/6i and PARPi shows synergistic effect in the inhibition of RB-deficient cells through ROS. **a**–**c** Colony formation of MDA-MB-468 (**a**, **b**) and MDA-MB436 (**c**) cells treated with 50 nM niraparib, 200 nM palbociclib, 2.5 mM NAC, or the combination of drugs as noted. *p-*values between drug treatments vs. control were calculated by the *t-*test. **d**–**g** Combination index and cell survival analyses of 7MDA-MB468 (**d**), MDA-MB436 (**e**), BT549 (**f**), and HCC1937 (**g**) cells treated every 2 days with niraparib and palbociclib (Nir + Pal) or Nir + Pal + NAC for 7 days. *p-*values between single treatment and combination treatments were calculated by the *t*-test. Data are from three independent experiments, mean ± SEM (represented by error bars). *p < 0.05, **p < 0.01, ***p < 0.001, by the student’s *t*-test. **h** Tumor volume of MDA-MB-231 (RB-proficient) xenografts treated with mock (vehicle), palbociclib, niraparib, or the combination of palbociclib and niraparib. **i** Body weight change percentage of the xenografts described in **h** with time for the duration of the experiment. **j**, **k** Tumor volume (**j**) and body weight change percentage (**k**) of MDA-MB-468 (RB-deficient) xenografts treated with mock, palbociclib, niraparib, or the combination of palbociclib and niraparib as noted. In all of the above xenograft model, *n* = 8, error bars represent the SEM.

Next, we determined whether NAC treatment diminished the synergistic effect in the inhibition of RB-deficient cells. Colony formation assays demonstrated that NAC diminished the synergistic effect resulting from the combination of PARPi and CDK4/6i (Fig. 7a–c and Supplementary Fig. S6S, T). Consistent with the observation above, NAC also blocked the short-term synergistic cytotoxicity stemming from the drug combination in the RB-deficient cells (Fig. 7d–g). The CIs ranged from about 2.5 to 8, indicating that NAC disrupted the synergistic effect between the combination (Fig. 7d–g, top panel).

Finally, we determined whether the combination of CDK4/6i and PARPi showed synergistic effect in xenograft mouse models. For RB-proficient MDA-MB-231 xenografts, as expected, the combination of palbociclib and niraparib yielded a synergistic tumor burden reduction compared with that of either drug alone (Fig. 7h) and was well tolerated (Fig. 7i). Importantly, the combined treatment of MDA-MB468 cells (RB-deficient) with palbociclib and niraparib also resulted in a synergistic reduction in tumor volumes, compared with either drug alone (Fig. 7j), with the drug doses used being tolerated (Fig. 7k). Further MS analyses indicated that there was no pharmacokinetic difference between single and combination treatment (Fig. S6U–W).

## Discussion

Although PARPis represent important potential tools for cancer treatment, novel strategies are required to improve their efficacy. To identify druggable candidate DRPCCs for combination with PARPis, we analyzed the correlation between the genome-wide expression of individual genes and mutations (the proxies of DNA damage) in a pan-cancer TCGA cohort. CDK4/6, which function as critical regulators of the cell cycle, showed the most significant negative correlation with mutation, suggesting that CDK4/6 possess the most significant ability to reduce DNA damage. Notably, combined inhibition of CDK4/6 and PARP showed synergy in both RB-proficient and RB-deficient cancer cells.

Our study also demonstrated that the combination of CDK4/6i and PARPi showed synergy in not only RB-proficient but also in RB-deficient breast cancer cells. Previous studies have demonstrated that HR DNA repair is highly suppressed in G1/S phase^38–40^, the progression through which is mediated by CDK4/6 in RB-proficient cancer cells. Therefore, it is not surprising that PARPi and CDK4/6i combinations are highly effective in RB-proficient cancer cells. However, it was not anticipated that this combination would also exhibit synergy in RB-deficient cells. If the combination shows acceptable tolerability in humans as it does in mice, the combination would therefore be expected to demonstrate activity in the great majority of patients with breast cancer. Our findings thus suggest that the combination could potentially expand the spectrum of patients likely to benefit from PARPi or CDK4/6i alone beyond those with tumors carrying HR defects or that are RB-proficient. Furthermore, considering the pro-survival function of cyclin D-CDK4/6^21,22^, we propose that measuring the levels of cyclin D and/or CDK4/6 might help to predict the likelihood of synergistic interactions between CDK4/6i and PARPi.

Although CDK4/6i has been approved by the FDA, the molecular mechanism of CDK4/6i in cancer treatment has remained elusive. As CDK4/6 regulate the G1/S transition, it is logical that the CDK4/6i causes cell cycle arrest in RB-proficient cancer cells^23,24^. Conversely, the CDK4/6i does not halt the proliferation of RB-deficient cells, supporting the role of RB as the rate-limiting substrate of CDK4/6^21,31^. Unexpectedly, inhibition of CDK4/6 also triggers the apoptosis of RB-deficient cancer cells^42,43^. These studies strongly support that CDK4/6 may also control RB-deficient cells in a cell cycle-independent manner. Moreover, during the preparation of this manuscript, two research groups reported that CDK4/6 could regulate metabolism in a cell cycle-independent manner^32,44^. Consistent with our observation, one group described that the inhibition of CDK4/6 enhances ROS in RB-deficient cells through a cell cycle-independent mechanism. Thus, CDK4/6 represents an oncoprotein that affects cancer cells in both cell cycle-dependent and -independent manners, with the cell cycle-independent property to be exploited for the development of new strategies.

Several limitations of this study should be noted. Firstly, although we show that the increase of ROS by the combination of CDK4/6i and PARPi is the dominant mechanism to synergistically inhibit RB-deficient cancer cells, we cannot exclude other mechanisms that may also cooperate or contribute to this synergistic effect. For example, CDK4/6 has been reported to phosphorylate DNMT1 and block its autophagy-dependent degradation^45^. Therefore, it remains necessary to further characterize the means by which other mechanisms cooperate or contribute to the CDK4/6i and PARPi synergistic effect in RB-deficient cancer cells. Secondly, although pan-cancer analysis revealed that the combination of CDK4/6i and PARPi might potentially show synergy in multiple cancer types, the primary focus of the current study was on breast cancer. This was because the FDA has approved a CDK4/6i (palbociclib) for the treatment of breast cancer, in which PARPis have also shown promising clinical activity. However, we believe that the combination of CDK4/6i and PARPi is highly likely to show synergy in other cancer types as well. Despite these limitations, our findings provide a rationale for clinical application of PARPi in the setting of combination with CDK4/6i to treat breast cancer.

## Supplementary information

Figure S1

Figure S2

Figure S3

Figure S4

Figure S5

Figure S6

Supplementary Figure Legends

Table S1-3

Table S4

Table S5

Table S6-17

Table S18

Supplementary Table Legend

Table S1

Table S2

Table S3

Table S4

Table S5

Table S6

Table S7

Table S8

Table S9

Table S10

Table S11

Table S12

Table S13

Table S14

Table S15

Table S16

Table S17

Table S18
