# Supplementary figures and images for: Pan-cancer analysis reveals synergistic effects of CDK4/6i and PARPi combination treatment in RB-proficient and RB-deficient breast cancer cells
Source: Cell Death Dis. 2020 Apr 6;11(4):219. doi: 10.1038/s41419-020-2408-1 (PMC7136254; doi:10.1038/s41419-020-2408-1)

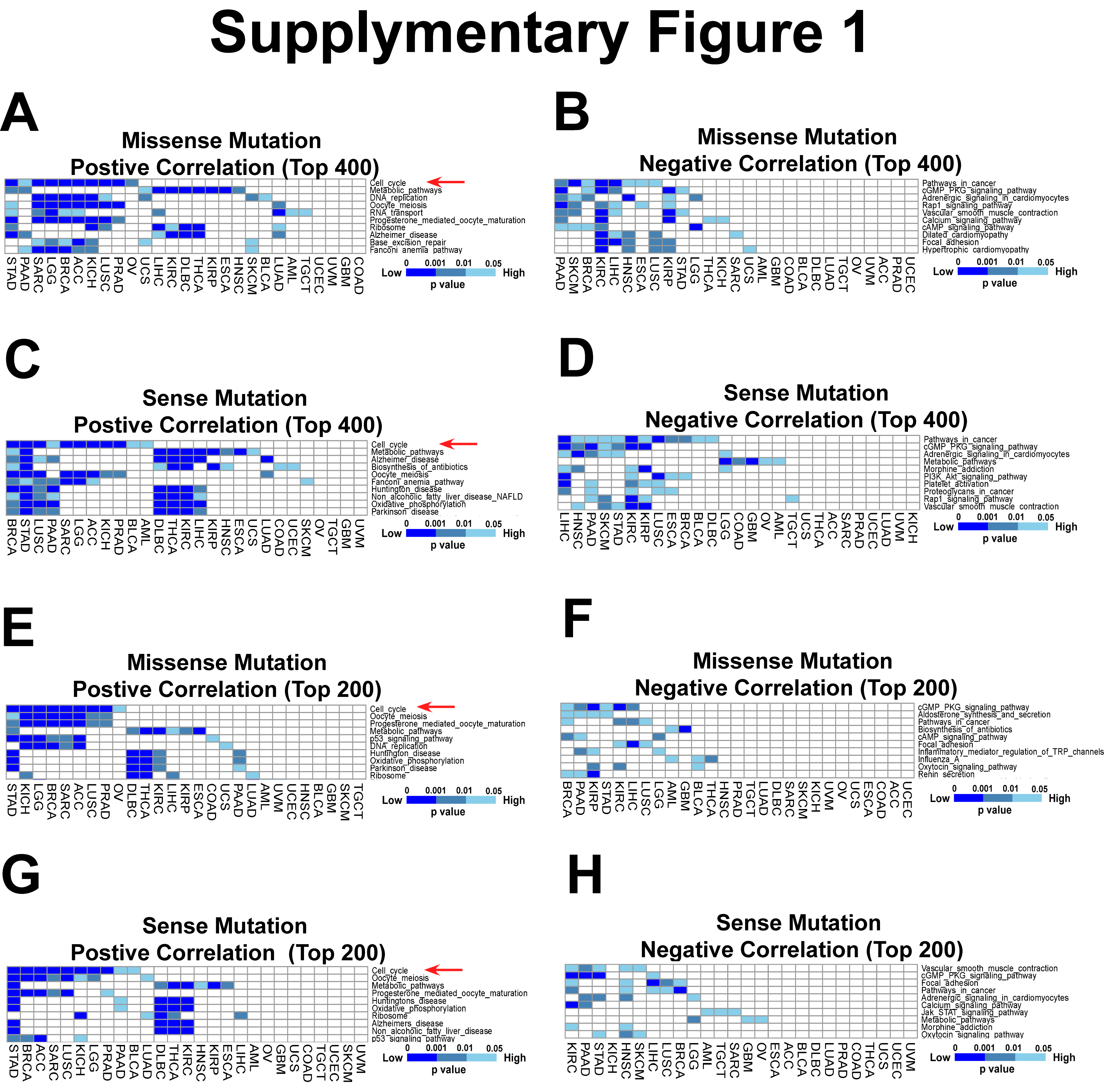

Supplement: Supplementary file 1 — Figure S1 [file 41419_2020_2408_MOESM1_ESM.tif]

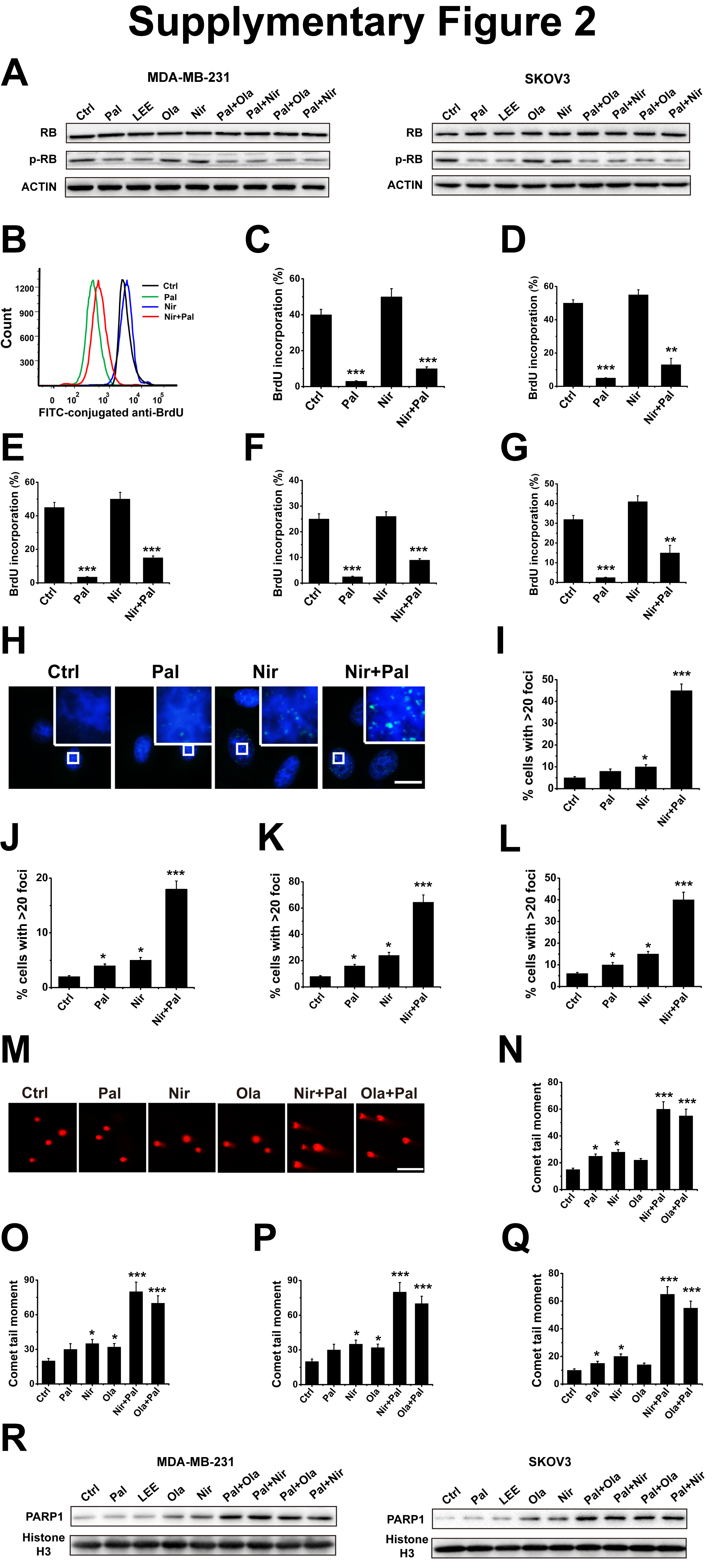

Supplement: Supplementary file 2 — Figure S2 [file 41419_2020_2408_MOESM2_ESM.tif]

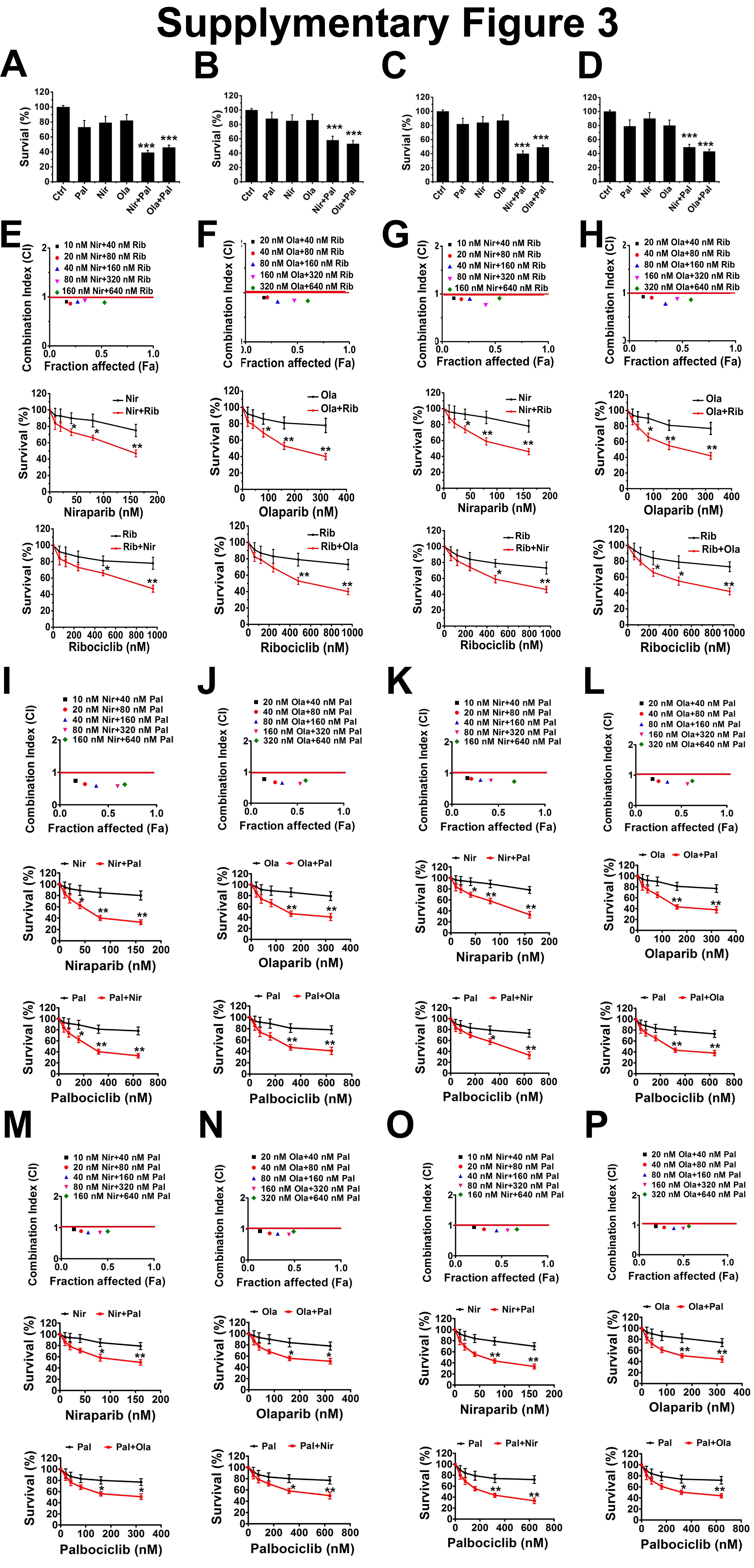

Supplement: Supplementary file 3 — Figure S3 [file 41419_2020_2408_MOESM3_ESM.tif]

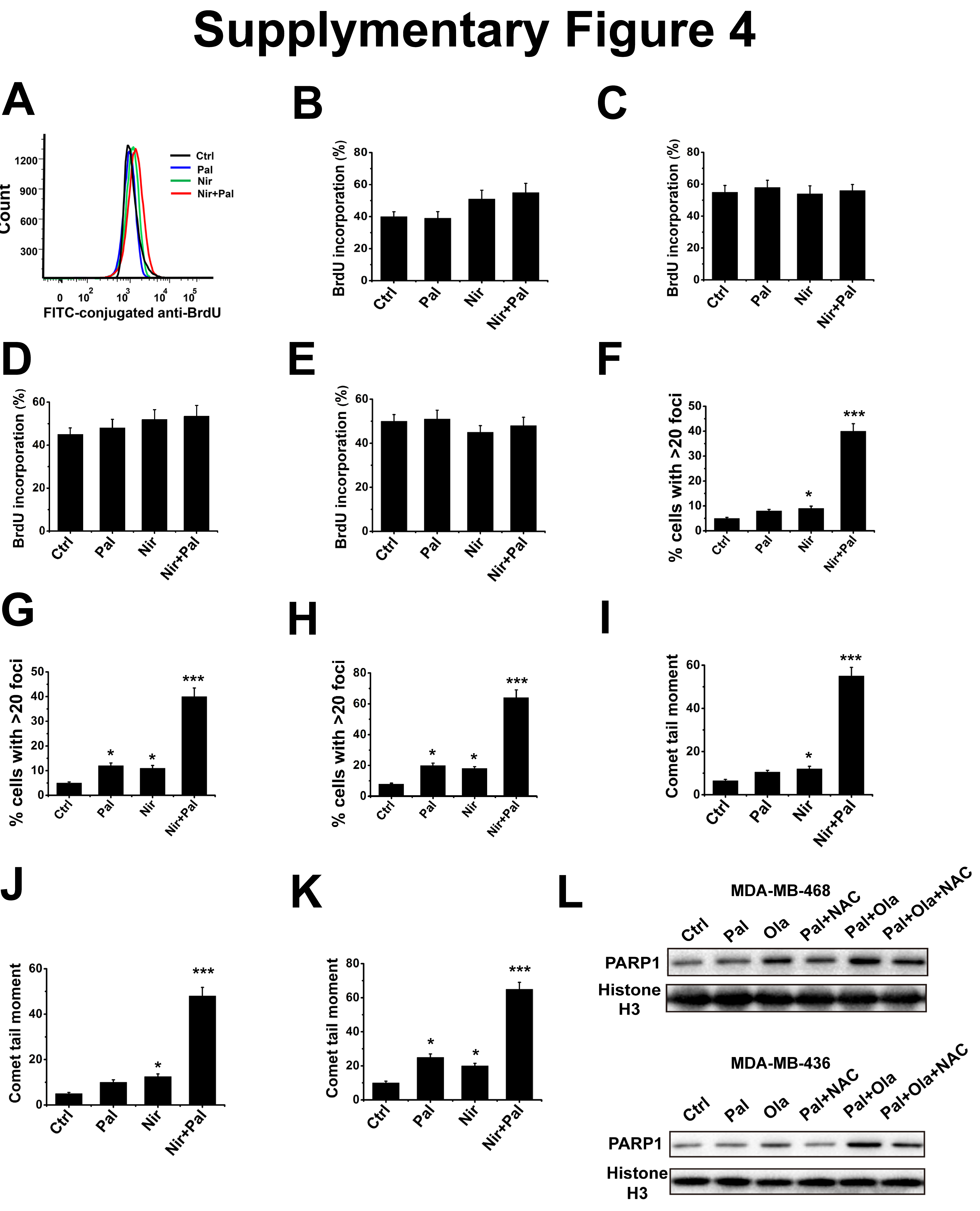

Supplement: Supplementary file 4 — Figure S4 [file 41419_2020_2408_MOESM4_ESM.tif]

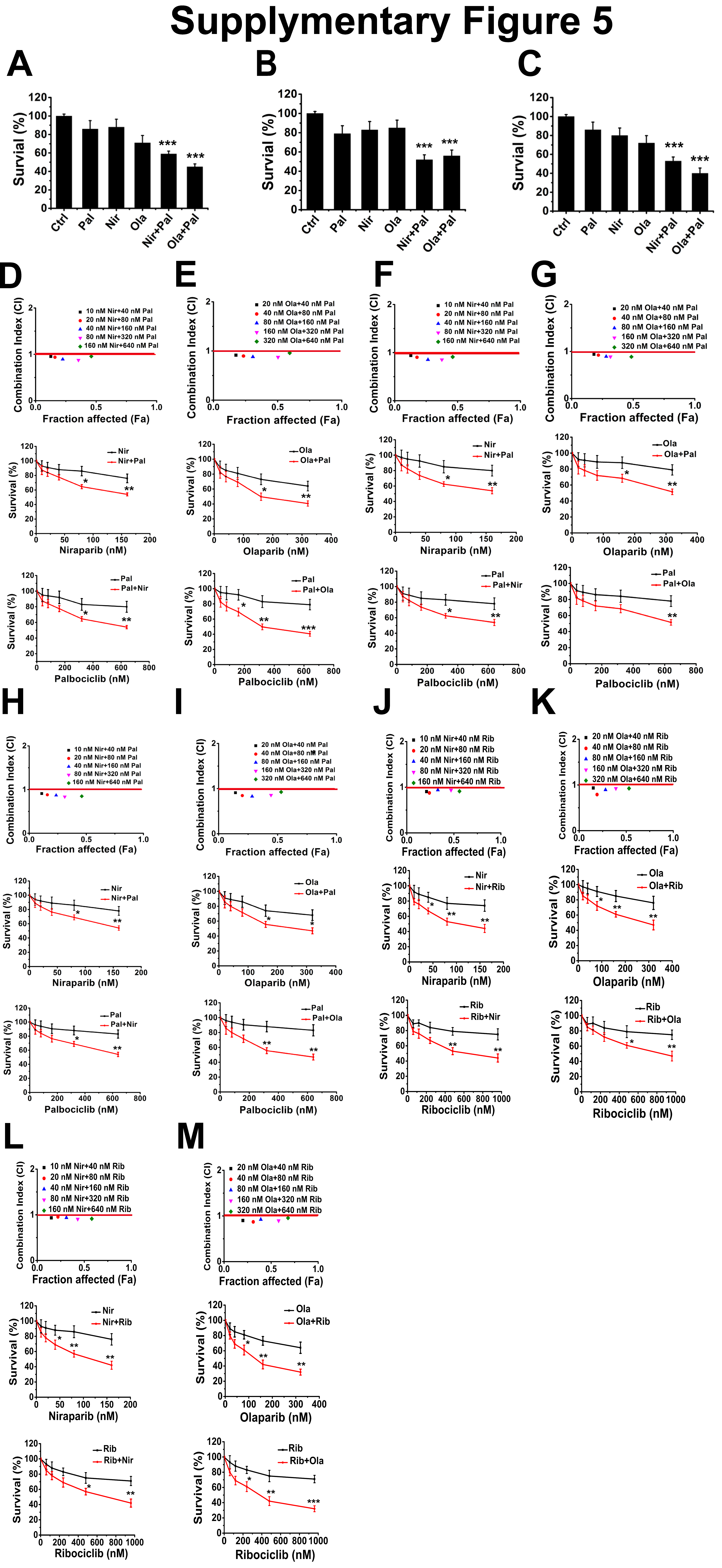

Supplement: Supplementary file 5 — Figure S5 [file 41419_2020_2408_MOESM5_ESM.tif]

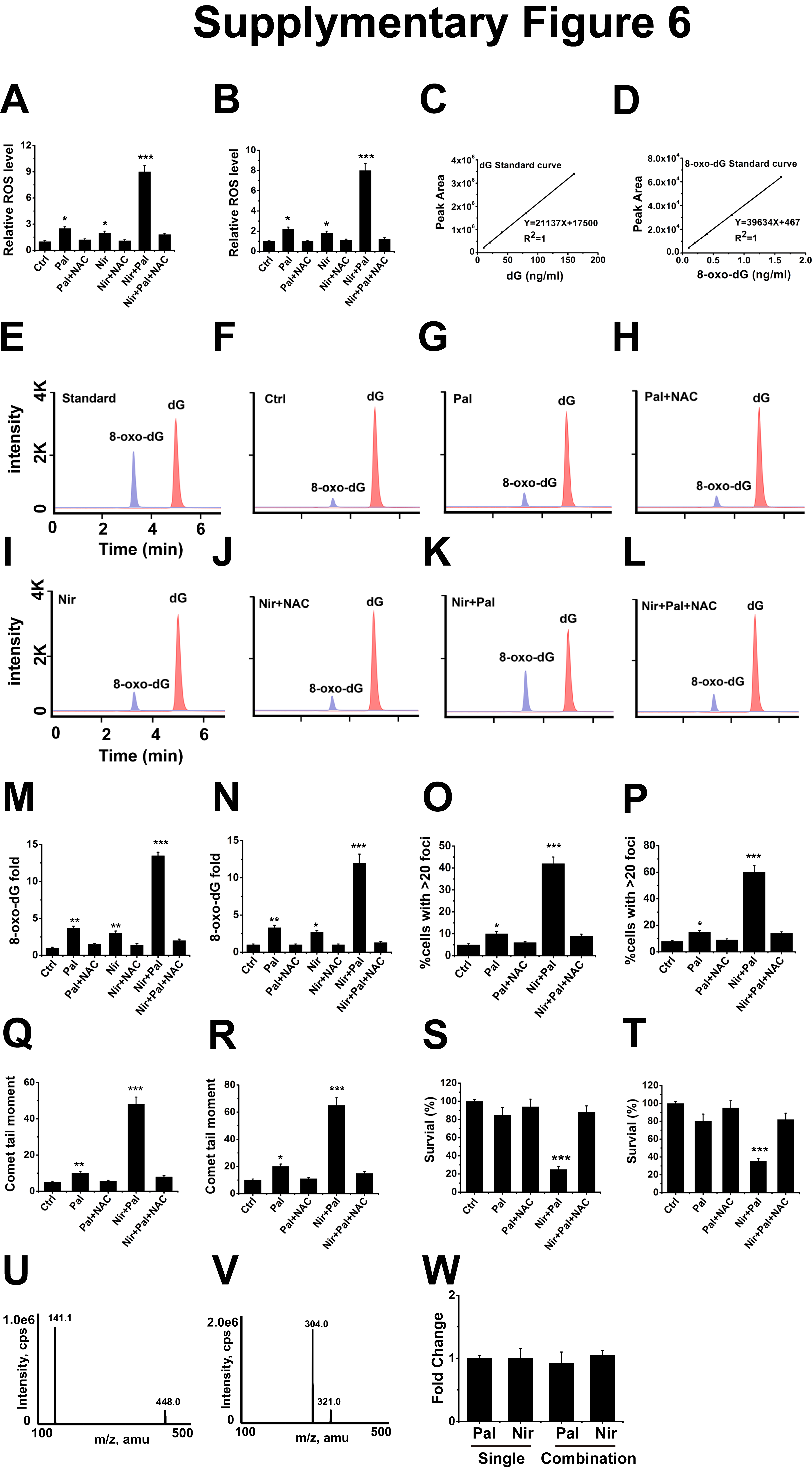

Supplement: Supplementary file 6 — Figure S6 [file 41419_2020_2408_MOESM6_ESM.tif]
