# Supplementary material for: Pan-cancer analysis reveals synergistic effects of CDK4/6i and PARPi combination treatment in RB-proficient and RB-deficient breast cancer cells
Source: Cell Death Dis. 2020 Apr 6;11(4):219. doi: 10.1038/s41419-020-2408-1 (PMC7136254; doi:10.1038/s41419-020-2408-1)
Supplement: Supplementary file 7 — Supplementary Figure Legends [file 41419_2020_2408_MOESM7_ESM.docx]

**SUPPLEMENTARY FIGURE LEGENDS**

**fig. S1. Cell cycle pathway shows the most significant positive correlation with mutation.** (**A** to **H**) Heatmap depicting the top 10 enriched pathways of the top 400/200positive/negative genes correlated with missense or sense mutations across 27 cancer types.

**fig. S2. Combination of CDK4/6i and PARPi synergistically enhanced DNA damages in RB-proficient cells.** (**A**) RB, pRB and ACTIN (loading control) in MDA-MB-231 and SKOV3 cells treated with 50 nM niraparib, 100 nM olaparib, 200 nM palbociclib, 300nM ribociclib or the combination of them as noted for 24 hours. (**B**) Representative flow cytometric analysis of BrdU incorporation in MDA-MB231 cells treated with 50 nM Niraparib, 200 nM Palbociclib, or the combination of Niraparib and Palbociclib for 24 hrs. Cells were pulse-labeled with BrdU for 1 h. (**C** to **G**) BrdU incorporation percentage of MDA-MB231 (C), MCF7 (D), SKOV3 (E), HeLa (F), and Hs578T (G) cells treated as described in B. (**H** to **L**) γ-H2AX focus analyses of MCF7 (H and I), SKOV3 (J), HeLa (K) and Hs578T (L) cells treated as described in Fig. 4D. Scale bar = 10 μm (H). (**M** to **Q**) Comet analyses of MCF7 (M and N), SKOV3 (O), HeLa (P) and Hs578T (Q) cells treated as described in Fig. 4F. Scale bar = 50 μm (M). (**R**) PARP1 and histone H3 (loading control) in chromatin fractions from MDA-MB-231 and SKOV3 cells treated as described in A. Data are from three independent experiments, mean± SEM (represented by error bars). **p* < 0.05, ***p* < 0.01, ****p* < 0.001, by the student’s *t*-test, drugs treatments versus control.

**fig. S3. Combination of CDK4/6i and PARPi showed synergistic effect in the inhibition of multiple RB-proficient cell lines.** (**A**, **B**, **C** and **D**) Colony formation analyses of MCF7 (A), SKOV3 (B), HeLa (C) and Hs578T (D) cells treated as described in Fig. 4H. (**E**, **F**, **G** and **H**) Combination index (Top) and cell survival analyses of MDA-MB-231 (E and F) and SKOV3 (G and H) cells treated every two days with niraparib (10–160 nM)/ olaparib (20–320 nM) and ribociclib (60–960 nM), or the combination of those at a constant ratio (niraparib: ribociclib = 1: 6, olaparib: ribociclib = 1: 3) for 7 days. (**I**, **J**, **K** and **L**) Combination index (Top) and cell survival analyses of MCF7 (I), SKOV3 (J), HeLa (K) and Hs578T (L) cells treated as described in Fig. 4I. (**M**, **N**, **O** and **P**) Combination index (Top) and cell survival analyses of MCF7 (M), SKOV3 (N), HeLa (O) and Hs578T (P) cells treated as described in Fig. 4L. Data are from three independent experiments, mean± SEM (represented by error bars). **p* < 0.05, ***p* < 0.01, ****p* < 0.001, by the student’s *t*-test, single treatment versus combination treatments.

**fig. S4**. **Combination of CDK4/6i and PARPi synergistically enhanced DNA damages in RB-deficient cells.** (**A** to **E**) BrdU incorporation of MDA-MB468 (A and B), MDA-MB436 (C), BT549 (D), HCC1937 (E) cells treated 50 nM Niraparib and/or 200 nM Palbociclib for 24 hrs. Then cells were pulse-labeled with BrdU for 1 h. (**F**, **G** and **H**) γ-H2AX focus analyses of MDA-MB436 (F), BT549 (G) and HCC1937 (H) cells treated as described in Fig. 5D. (**I, J** and **K**) Comet analyses of MDA-MB436 (I), BT549 (J) and HCC1937 (K) cells treated as described in Fig. 5F. (**L**) PARP1 and histone H3 (loading control) in chromatin fractions from MDA-MB-468 and MDA-MB-436 cells treated with 100 nM olaparib, 200 nM palbociclib, 5mM NAC or their combination as noted for 24 hours. Data are from three independent experiments, mean± SEM (represented by error bars). **p* < 0.05, ***p* < 0.01, ****p* < 0.001, by the student’s *t*-test, drugs treatments versus control.

**fig. S5. Combination of CDK4/6i and PARPi showed synergistic effect in the inhibition of multiple RB-deficient cell lines.** (**A**, **B** and **C**) Colony formation analyses of MDA-MB436 (A), BT549 (B) and HCC1937 (C) cells treated as described in Fig. 5H. (**D**, **F** and **H**) Combination index and cell survival analyses of MDA-MB436 (D), BT549 (F) and HCC1937 (H) cells treated as described in Fig. 5I. (**E, G** and **I**) Combination index and cell survival analyses of MDA-MB436 (E), BT549 (G) and HCC1937 (I) cells treated as described in Fig. 5L. (**J**, **K, L** and **M**) Combination index and cell survival analyses of MDA-MB-468 (J and K) and MDA-MB-436 (L and M) cells treated every two days with niraparib (10–160 nM)/ olaparib (20–320 nM) and ribociclib (60–640 nM), or the combination of those at a constant ratio (niraparib: ribociclib = 1: 6, olaparib: ribociclib = 1: 3) for 7 days. Data are from three independent experiments, mean± SEM (represented by error bars). *p < 0.05, **p < 0.01, ***p < 0.001, by the student’s t-test, single treatment versus combination treatments.

**fig. S6. Combination of CDK4/6i and PARPi shows synergistic effect in RB-deficient cells through ROS.** (**A** and **B**) ROS Levels in BT549 (A) and HCC1937 (B) cells treated as described in Fig. 6B. (**C**) Standard curve of dG for LC-MS/MS analysis. (**D**) Standard curve of 8-oxo-dG for LC-MS/MS analysis. (**E** to **L**) dG and 8-oxo-dG peaks of standards (E) and MDA-MB468 cells treated with mock (F), 200 nM Palbociclib (G), 200 nM Palbociclib + 2.5 mM NAC (H), 50 nM Niraparib (I), 50 nM Niraparib + 2.5 mM NAC (J), 50 nM Niraparib + 200 nM Palbociclib (K), or 50 nM Niraparib + 200 nM Palbociclib + 2.5 mM NAC (L) for 24 hrs. (**M** and **N**) 8-oxo-dG level of BT549 (M) and HCC1937 (N) cells treated as described in Fig. 6G. (**O** and **P**) γ-H2AX focus analyses of BT549 (O) and HCC1937 (P) cells treated as described in Fig. 6J. (**Q** and **R**) Comet analyses of BT549 (Q) and HCC1937 (R) cells treated as described in Fig. 6M. (**S** and **T**) Colony formation analyses of BT549 (S) and HCC1937 (T) cells treated as described in Fig. 7B. (**U** and **V**) Base ion mass transitions for LC-MS-MS analysis of Palbociclib(U) and Niraparib(V) standard. The MRM transitions were monitored as follows: 448.0 to 141.1 (Palbociclib); 321.0 to 304.1 (niraparib). (**W**) LC–MS/MS assay of pharmacokinetic effects between single and combination treatment. Data are from three independent experiments, mean± SEM (represented by error bars). **p* < 0.05, ***p* < 0.01, ****p* < 0.001, by the student’s *t*-test, drugs treatments versus control.
