# Supplementary material for: Pan-cancer analysis reveals synergistic effects of CDK4/6i and PARPi combination treatment in RB-proficient and RB-deficient breast cancer cells
Source: Cell Death Dis. 2020 Apr 6;11(4):219. doi: 10.1038/s41419-020-2408-1 (PMC7136254; doi:10.1038/s41419-020-2408-1)
Supplement: Supplementary file 8 — Table S1-3 [file 41419_2020_2408_MOESM8_ESM.pdf]

table S1. Summary of TCGA cancer types, patients, and all mutation loads surveyed in this study.

Tumor type	Patient Number	A>C	A>G	A>T	C>A	C>G	C>T	INS	DEL	Mutation	Mutation (average)
AML	164	66	230	85	237	130	1204	155	64	2171	13.24
ACC	77	690	2525	550	2595	1604	5739	3312	9993	27008	350.75
BLCA	129	772	2410	985	3662	10382	19571	315	882	38979	302.16
LGG	286	338	1448	378	765	792	5467	101	556	9845	34.42
BRCA	978	7674	7940	3296	12114	14747	35922	2915	4156	88764	90.76
COAD	204	4948	8407	1840	15524	1572	40862	1042	1619	75814	371.64
ESCA	184	3057	5520	2355	14349	4393	20569	1596	4062	55901	303.81
GBM	148	334	1239	476	1023	758	6563	144	415	10952	74.00
HNSC	504	3049	12130	6194	18075	19122	52937	2097	4765	118369	234.86
KICH	66	254	1936	286	674	389	3177	47	94	6857	103.89
KIRC	426	2207	4981	4243	5423	3108	10152	9243	2932	42289	99.27
KIRP	281	1282	3119	1774	2858	2137	4868	595	1954	18587	66.15
LIHC	367	2377	13617	6044	8664	3285	12573	878	4281	51719	140.92
LUAD	230	2003	5756	6167	25478	9950	18620	1070	2020	71064	308.97
LUSC	177	1763	6006	4920	20781	10161	20510	0	5	64146	362.41
DLBC	48	936	2922	680	1438	1405	8894	166	374	16815	350.31
OV	316	1140	2269	1553	3425	3176	6602	221	646	19032	60.23
PAAD	150	1445	2872	587	5838	677	18655	59	175	30308	202.05
PRAD	498	1499	4887	1437	4391	2245	21970	1056	2855	40340	81.00
SARC	245	617	1764	1143	2480	1462	10645	570	1184	19865	81.08
SKCM	363	5888	12031	7083	7840	5169	278247	1032	3083	320373	882.57
STAD	371	10994	27705	5601	18058	5579	87570	6185	24750	186442	502.54
TGCT	155	520	1858	433	5078	844	4351	437	754	14275	92.10
THCA	403	235	1240	590	768	811	3379	56	265	7344	18.22
UCS	57	999	1273	271	2235	671	4525	327	883	11184	196.21
UCEC	239	11585	19282	2540	46457	2243	95990	748	2051	180896	756.89
UVM	80	96	209	115	166	133	1266	24	158	2167	27.09
Total	7146	66768	155576	61626	230396	106945	800828	34391	74976	1531506	214.32

For each data type and each cancer type, the numbers of patients and mutations available in the analysis are shown. Base-substitution mutations include A>C, A>G, A>T, C>A, C>G and C>T substitution. INS and DEL represent insertion and deletion mutations, respectively.

table S2. Summary of cancer types, patients, and missense mutation loads.

Tumor type	Patients Number	A>C	A>G	A>T	C>A	C>G	C>T	INS	DEL	Mutation	Mutation (average)
AML	164	52	170	70	182	105	805	155	64	1603	9.77
ACC	77	226	1205	118	664	513	2459	3312	9993	18490	240.13
BLCA	129	615	1697	832	2946	8793	12545	315	882	28625	221.90
LGG	286	272	1068	305	631	670	3770	101	556	7373	25.78
BRCA	978	5438	5106	2645	9514	12060	22542	2915	4156	64376	65.82
COAD	204	4584	5952	1594	15057	1366	29676	1042	1619	60890	298.48
ESCA	184	2266	3397	1780	10791	3364	12790	1596	4062	40046	217.64
GBM	148	265	853	394	827	626	4410	144	415	7934	53.61
HNSC	504	2375	8046	4985	13928	15601	33534	2097	4765	85331	169.31
KICH	66	34	111	78	145	72	718	47	94	1299	19.68
KIRC	426	1794	3211	3554	4239	2530	6375	9243	2932	33878	79.53
KIRP	281	1114	2104	1454	2215	1773	3080	595	1954	14289	50.85
LIHC	367	1926	9663	4874	6785	2766	8174	878	4281	39347	107.21
LUAD	230	1638	4162	4622	18511	8127	11249	1070	2020	51399	223.47
LUSC	177	1427	4174	3957	16336	8497	13234	0	5	47630	269.10
DLBC	48	726	1676	544	1024	1080	4847	166	374	10437	217.44
OV	316	937	1609	1277	2783	2696	4322	221	646	14491	45.86
PAAD	150	1296	2108	501	4922	606	12555	59	175	22222	148.15
PRAD	498	1153	3117	1139	3467	1733	14124	1056	2855	28644	57.52
SARC	245	432	1101	816	1791	1091	6273	570	1184	13258	54.11
SKCM	363	4764	7646	5920	5791	4079	171033	1032	3083	203348	560.19
STAD	371	8964	18784	4611	14174	4460	57474	6185	24750	139402	375.75
TGCT	155	280	1019	297	3750	596	2445	437	754	9578	61.79
THCA	403	184	890	538	604	664	2159	56	265	5360	13.30
UCS	57	880	854	211	1885	558	3006	327	883	8604	150.95
UCEC	239	10031	13103	2134	39623	1912	63311	748	2051	132913	556.12
UVM	80	83	152	100	131	106	866	24	158	1620	20.25
Total	7146	53756	102978	49350	182716	86444	507776	34391	74976	1092387	152.87

For each data type and each cancer type, the numbers of patients and mutations available in the analysis are shown.

table S3. Summary of cancer types, patients, and sense mutation loads.

Tumor type	Patients Number	A>C	A>G	A>T	C>A	C>G	C>T	Mutation	Mutation (average)
AML	164	14	60	15	55	25	399	568	3.46
ACC	77	464	1320	432	1931	1091	3280	8518	110.62
BLCA	129	157	713	153	716	1589	7026	10354	80.26
LGG	286	66	380	73	134	122	1697	2472	8.64
BRCA	978	2236	2834	651	2600	2687	13380	24388	24.94
COAD	204	364	2455	246	467	206	11186	14924	73.16
ESCA	184	791	2123	575	3558	1029	7779	15855	86.17
GBM	148	69	386	82	196	132	2153	3018	20.39
HNSC	504	674	4084	1209	4147	3521	19403	33038	65.55
KICH	66	220	1825	208	529	317	2459	5558	84.21
KIRC	426	413	1770	689	1184	578	3777	8411	19.74
KIRP	281	168	1015	320	643	364	1788	4298	15.30
LIHC	367	451	3954	1170	1879	519	4399	12372	33.71
LUAD	230	365	1594	1545	6967	1823	7371	19665	85.50
LUSC	177	336	1832	963	4445	1664	7276	16516	93.31
DLBC	48	210	1246	136	414	325	4047	6378	132.88
OV	316	203	660	276	642	480	2280	4541	14.37
PAAD	150	149	764	86	916	71	6100	8086	53.91
PRAD	498	346	1770	298	924	512	7846	11696	23.49
SARC	245	185	663	327	689	371	4372	6607	26.97
SKCM	363	1124	4385	1163	2049	1090	107214	117025	322.38
STAD	371	2030	8921	990	3884	1119	30096	47040	126.79
TGCT	155	240	839	136	1328	248	1906	4697	30.30
THCA	403	51	350	52	164	147	1220	1984	4.92
UCS	57	119	419	60	350	113	1519	2580	45.26
UCEC	239	1554	6179	406	6834	331	32679	47983	200.77
UVM	80	13	57	15	35	27	400	547	6.84
Total	7146	13012	52598	12276	47680	20501	293052	439119	61.45

For each data type and each cancer type, the numbers of patients and mutations available in the analysis are shown.
