# Supplementary material for: Pan-cancer analysis reveals synergistic effects of CDK4/6i and PARPi combination treatment in RB-proficient and RB-deficient breast cancer cells
Source: Cell Death Dis. 2020 Apr 6;11(4):219. doi: 10.1038/s41419-020-2408-1 (PMC7136254; doi:10.1038/s41419-020-2408-1)
Supplement: Supplementary file 9 — Table S4 [file 41419_2020_2408_MOESM9_ESM.pdf]

table S4. Genes showing positive correlation with mutation load in at least 5 types of cancer.

Symble	BRCA	ACC	SARC	LUAD	STAD	LGG	PRAD	SKCM	LUSC	UCEC	COAD	KICH	PAAD	UCS	BLCA	KIRC	OV	UVM	TGCT	THCA	LIHC	GBM	HNSC	DLBC	ESCA	KIRP	AML	Count
CDCA2	158	57	71	313	51	205	283	65	388	337	325																	11
PRC1	85	71	202	213	163	317	145	194		270		127	308															11
CDCA8	15	35	70	71	110	180	60		144			244	168															10
CDKN3	37	85	62	125	46			357	15	251		316		267														10
CENPA	7	9	59	19	241	133	263	155	47						166													10
DLGAP5	63	4	129	126	104	216	147	397	83			357																10
MELK	41	125	33	92	31	135	160						305	39	175													10
AURKA	29	191	4	60		88	95		50							165	254											9
BIRC5	22	121	61	106	176	202		325						362				300										9
CDC20	2	52	27	18	269		387		8				387	146														9
CDC45	13	10	18	158	146	62				138			398				99											9
CDK1	51	55	263		84	208	72		180				171		324													9
CEP55	53	31	14	26	35	215	137	20					373															9
CKS2	91	175	78		60	16			110				27		182	307												9
GSG2	311	69	1	173		122				187	140	323						277										9
KIF18A	179	108	25	398	206	285	378	266						184														9
KIF4A	47	56	9	250	381	163	195					381	329															9
NCAPH	49	154	12	17	224	193	309		84										230									9
NDC80	126	26	345	37	199	96			166					68	164													9
NEK2	141	15	169	22	126		107	192	2										120									9
NUF2	76	70	31	8		137	369		46				389		124													9
OIP5	99	366	330	359	65	145		340					23		82													9
RAD51AP1	109	262	395	46	116	270	295		135						79													9
SGOL1	93	109	44	52		98	158	289	10						56													9
SGOL2	173	11	221	27		78	48	193	266						278													9
SPC24	105	117	294	324		184				400		112	193						4									9
TROAP	5	42	79	63		231			9			371	190		377													9
UBE2T	32	227	259	48	101			186	114				12		42													9
BUB1	62	97	156	33	153	71	154		66																			8
BUB1B	230	16	321	302	102	267	90					310																8
CCNA2	34	75	103	309	2	183	354		275																			8
CCNB1	89	131	32		19	255	102		38							123												8
CCNB2	33	17	55	117	23	139						259	281															8
CDC25C	69	135	147	264	80	222	114		75																			8
CDCA5	14	36	13	77			311		252			87						151										8
CENPE	297	319	223	270	382	292	193	337																				8
CENPL	97	53	96	59		8	352		146							354												8
CKAP2L	204	82	130	10	118	146	143	121																				8
DEPDC1	140	68	104	127	10	105		91	191																			8
EBP	225		122										150	71	44	247				359	255							8
EXO1	36	34	157	136	70	69	346		205																			8
GTSE1	23	8	39	198		195	310			57		154																8
HJURP	11	27	90	23	139	138	150		59																			8
IQGAP3	87	137	306			210	396	276		288			118															8
KIF11	244	13	355	194	48		27	181					247															8
MCM10	88	122	45	13	284	362	398		227																			8
NCAPG	64	59	64	144	30	54	265		98																			8
OTX1	175	79		357						88	191				211					221		103						8
PKMYT1	48	132	131	276		55				160		73	141															8
POC1A		333	167		47	370			51				133					14	340									8
RACGAP1	196	48	145	171	175		77		124				399															8
RAD54L	71	203	117	67	330	346		87					208															8
RRM2	72	74	166		36	162	175		393	27																		8
SHCBP1	163	113	51	294	120	265				351							29											8
SKA1	144	266	37	174	44	291	242							176														8
SKA3	60	226	309	66	152	340	41		360																			8
TPX2	20	94	6	29		48	71		14				317															8
UBE2C	1	83	3	50		213			57				161		378													8
ZWINT	130	81		209	105			22					117		58					182								8
ARHGAP11A		127	351		88	237	100	125				379																7
ASPM	169	21	316	110		124	110		279																			7
AURKB	18	39	22	39	372	251				315																		7
CDCA3	10	30	287	64					78			396			300													7
ERCC6L	177	90	16			186	97		181			207																7
EZH2	121	167			117	34	383						376		381													7
FAM64A	59		50	16	166	158	203												367									7
FAM72B	78	103	54	38				138	39						280													7
FAM72D	269	92	101	32				182	120			395																7

FEN1	101	47	246							245		269		59	356													7
FLAD1						233			211				105				370			97	63		98					7
FOXM1	25	77	60	73		72	397					94																7
HDGF	249		140	1					118				9		317						40							7
KIF15	274	145	331	42	334	134						254																7
KIF20A	27	78	257		288	194	35		155																			7
KIF23	167	5	113	57	243	248		207																				7
KIF2C	24	62	26	9	140	223			147																			7
KIFC1	6	25	174	31		198			348										348									7
LAGE3	309								16			308		297	14						20			226				7
MTFR2	9	239	34	30	66	380			400																			7
MTHFD2	115			304	41				299		120				92								226					7
ORC6	54		215	290	187								201		126		274											7
PBK		220	47		4	177	234	168		336																		7
PLK1	16	12	28	109	239				340				269															7
PPAT				101	71	310	9										180						384				252	7
RAD51	200	51		354	12	155				122			152															7
RPA3			65						129				51	37	95	81					114							7
TRIP13	70	50	15	14	200	111	116																					7
TTK	31		48	15	220	247	15	240																				7
TYMS		87		83	17					346	19			61	400													7
ACTL6A	189			182		245		133							178				178									6
ASF1B	86	102			215	53				385									220									6
AUNIP	267		10	28	211				19						130													6
BCL2L12			191	164								47	288		53						324							6
BRI3BP							317			39				218	154	326									371			6
C5ORF34	300	136	21	134		355			398																			6
CDC25A	166		149	315	245	282			69																			6
CDT1	35	159								8		243	113					21										6
CENPF	180	120	297	132		128	37																					6
CENPM	102	72	199		53					41				123														6
CHAF1A	392		143			103				15		261					281											6
CHEK2	259	20		334	217	203								353														6
DDIAS		261	17	345		1	211								229													6
DEPDC1B	61		280			211	73		108											267								6
E2F1	21			178					42			198	195		52													6
EIF2AK1							179		109				3	73		155				65								6
FAM72A	203	119	205	21				294	162																			6
FANCI	343	310	183	332		174							298															6
FBXO5	252	112		121	275	296													40									6
GMNN	321	209	80		165			248						342														6
LIN9	375	126		191	229	315																					360	6
LMNB2	146	66	146	277		14				377																		6
LONP1										25		174		122		246				196		13						6
LRR1	232	61	224		112	161								147														6
MAD2L1	108		35		3	73						262					226											6
MCM6	187	210		93	143	30								224														6
MDH1	397				369										180	250				99				317				6
MKI67	191	33	195			368	118						220															6
MRPL13	395				98				264						308	14				249								6
MYBL2	4	86	11	3		171			18																			6
NCAPG2		326		223	185	197	207					130																6
ORC1	145	253	97	218	127	84																						6
PARPBP	198		86	169	309	38						362																6
PCNA	282				287	377			392	74				136														6
PLEK2					285	379					374		115	112											291			6
PLK4	295	313	109	292	59	361																						6
POLD1		141	289									241		174	116			84										6
PPM1G	199	318				352							340		10			396										6
RAD18			398			13	149	135	36						179													6
RHEBL1		49		152				58	366					257	250													6
SLC35A2	326		188										170		9	344									384			6
SPC25	55	2	100	153	15									280														6
STIL	131	93	163			199	191	54																				6
TACC3	65	6	57	303		114			378																			6
TK1	182	271	125	341						391							107											6
TOP2A		115	307	24		176	66												215									6
TUBA1B	42		244		395				295			235		90														6
UBE2S	46	18	42	284					58			46																6

UCK2	284		253						324				59		328								128					6
UQCRC1					362					268				93				329			391			237				6
WDR12				99	336	308										79				173			347					6
ZNF367	396					60				231				135	186				250									6
ZNF695	107			25			227		223						288								155					6
ACP1	213				232										1	214			18									5
ALG3	50														270			278			204		312					5
ANLN	94		84	311			132	332																				5
ARHGEF39	160	276		317					243					11														5
ATP5G3	257				40											400				338			266					5
BLM	208	296	87	79			237																					5
BUB3		105			108			204			124		100															5
C17ORF53	368	190	24	187		187																						5
CACYBP	305			248	182				32						30													5
CASC5		267			178	397	210	316																				5
CCNE2	310			217					370	259					226													5
CCT5	111		112	91									60			151												5
CDC6			94	181			165			375							84											5
CDK4	283											33		256		199				19								5
CENPQ			88	275	380			147									225											5
CHCHD3			116			320							386			183				37								5
DDX39A	83	104	81	305		289																						5
DIAPH3	266	211		88	122		359																					5
DNA2	315	257		356				195	177																			5
DONSON	124			154	400				303	303																		5
DSCC1	154			62		80			121						103													5
DTL	294	285			121	118	215																					5
E2F2	219	218		227	390	281																						5
EME1	170		7	36		160											227											5
ESCO2		270	164		49	246	220																					5
ESPL1	129	260	226	75		218																						5
FAM83D	28	98		160								161											134					5
FANCA	188					200				20			44								351							5
GDF15						258				279			209			293									331			5
GINS2					68					22					245			321						116				5
GMPS	227			202	297		29																77					5
H2AFZ	90				1					111		120			31													5
IARS2					173		88												312	82			14					5
ILF2		32	377	206																314			136					5
KIAA0101	325	196			263	313				298																		5
KIAA1524	164		134	34		350		223																				5
KIF14	110	29		85		126	67																					5
KIF18B	77	19	73	68						338																		5
KPNA2	119		8	6	125				54																			5
LMNB1	162	63			394	136	167																					5
MAGOHB	347		348						151						87	208												5
MCM5	291	215	291							50								66										5
MIS18A	74		298	355		234			327																			5
MRPL11	306											28			309						113			223				5
MRPL14	320												127		258						18			306				5
MRPL15	277				227				333							396				192								5
MRPL51	234														370	242				374	365							5
MVD										18			175					217			192			131				5
NDUFB9	349															330				57	329			321				5
NEIL3	233	60			57	278	129																					5
NOL10				265		101		60	318														326					5
NOP10										61		355	353		157					235								5
NUDT8	394									171			273	16		393												5
PAICS				143	168		315					398															368	5
PAIP1			365	280												99			107	81								5
PAQR4										77		176	182						232	262								5
PFDN2	193			229									1		64						179							5
POLE2	350			282	95			343					251															5
POLQ	178		114	135		82									231													5
POP4			74						326				391			182					172							5
POP7	316		341										280		396						125							5
PSMD8			99									287				390				318	373							5
PTCD1						75										142				118	106		102					5
PTTG1	17	84			16	156																		253				5

PYCR1	356	283		120					260			115																5
RANBP1	183	363	102	260											364													5
RFC2	127		262			117				286		300																5
RFC4	117			360		104		12							19													5
RHEB												206	367		398		207				377							5
RRM1		398			387		243							148					270									5
SAC3D1	366								354					221	160		359											5
SASS6				142			377	118	283						335													5
SEC13	302														152			36			270				160			5
SHMT2	113	161			212											237							364					5
SPR														107		23		210		165			178					5
SUV39H1	192		5						74					391							57							5
TBRG4	288		152			154							34			55												5
TCF19	44					27		109		19							258											5
TICRR	66	40	20	103								279																5
TIMM50	280		187						174							267				134								5
TMEM106C								2							386	4		241		257								5
TNFSF9			66							29	49			21			131											5
TRAIP			325	90	155	323			25																			5
UHRF1	147	99	38	350						60																		5
WDR62	134		53	221		244				166																		5
ZWILCH		22	210		360		178	238																				5

The rank numbers of each positive gene in different cancer types are shown.
