# Supplementary material for: Pan-cancer analysis reveals synergistic effects of CDK4/6i and PARPi combination treatment in RB-proficient and RB-deficient breast cancer cells
Source: Cell Death Dis. 2020 Apr 6;11(4):219. doi: 10.1038/s41419-020-2408-1 (PMC7136254; doi:10.1038/s41419-020-2408-1)
Supplement: Supplementary file 10 — Table S5 [file 41419_2020_2408_MOESM10_ESM.pdf]

table S5. Genes showing negative correlation with mutation load in at least 5 types of cancer.

Symble	SARC	PRAD	KICH	BLCA	ACC	SKCM	LIHC	LUSC	KIRC	STAD	PAAD	ESCA	HNSC	LUAD	UCS	LGG	GBM	KIRP	THCA	UVM	BRCA	UCEC	OV	DLBC	COAD	TGCT	AML	Count
RCBTB2	14	59	99	116	180	190	278	371																				8
BOC					393	220			53	191	322	332	361															7
MAPK10			138				389				144			95	98	109	187											7
MRVI1						266	216	243	21	190						359		244										7
SYNPO2					25		143		137		367		168			42			165									7
ADGRA2						383	364		111	203								9		135								6
C1QTNF7							277	238	130	100			113	89														6
FCER1A	46			2	175			125						94							108							6
FRMD4A									152	215		83	133							347		359						6
GPRASP1		145					348			56								293		321	302							6
IGIP	274										221		73	258		25					27							6
IQCA1				15				262				153					21					1	398					6
MAGEE1					148					141	187	30						117						141				6
NRIP2	31			208		86				23											269				394			6
NT5C2							388	91							11	191							36	11				6
PDZD4	313						296		321	75		267		222														6
PRICKLE2					281		40			355	92					93						179						6
RASGRP2	254	308							15				228						235							393		6
SCN4B				287		60				91	239										149		71					6
SPATA18	13				71			44						3						215	45							6
TUB	183					180				219	70	20				160												6
ABAT	201		353		27																22				42			5
ADAMTS8	160								47	379				395					55									5
ADCY5	109					279				30	225					275												5
AFAP1L2									22									12			205	190	259					5
ANK3			306		331		67							385									282					5
ATP1B2						35			396	62								224				31						5
ATP8B2									7	385	166								289					183				5
BEX4	249										253	400				16						24						5
BTG2	400		111				358	356													173							5
CAB39L			101		100					331													346		65			5
CACNA1C				320		199	70		17									85										5
CBX7		334												96		236					43					228		5
CD1E				350	315			300			375			237														5
CEP68	371										50	135									187	122						5
CHST10										221	356	95										203	206					5
COL4A3			130		46		131						186		310													5
CORO2B						328				306	361											197	139					5
CYS1			112			318	202					204				14												5
DDB2	225	224		365				179						178														5
DLL1		326				336			322				191					375										5
DNAJC18										350	7	283										71	30					5
DYNC1I2	171		350					307							131					213								5
DZIP1							29			323	288	9										228						5
EFEMP1					258		109				289							7	125									5
EFS		123					133		244			253						259										5
EPHX2	10			360										341			132						149					5
ETS1							140		2				350					390						164				5
FLRT2							151						102					269	28	112								5
GNAO1									57	139						207		6					320					5
HSPB8				310						380	117		145			171												5
IKZF4	383				237						296										360		391					5
IL33				246							219			380					300							347		5
KIF26A			134	298		157	282											159										5
KLHDC1	84			30							106		347								2							5
LDLRAD4	12										337			352				361		235								5
LHFP	193								60	170			40					340										5
LRCH2	399								305		382							59		6								5
MYH11	382					347	246		81												345							5
NFASC	92								27	33								226							159			5
NME5	64				140		375							31							115							5
NTRK2				53			57					292				183					145							5
PEG3	87		91						383				381		211													5
PLEKHG1			303				187	400	42									67										5
RAPGEF4						277			398		98					331				86								5
RERG										240	208			365			352				140							5
RGS5					64	397			286	222											193							5
SALL2	114									46	82	103										180						5
SDPR										79	276									341	168	137						5
SERP2		222	250																4		256	58						5
SETBP1									18	27	317	274									13							5
SH3YL1	20						8								94								279	17				5
SLC22A17	386	16								105		164							203									5
SLIT3	369								44	302	125								164									5
SPRY1									64		83			240	292			359										5
SULT1C4						371	262			148					210							30						5
TCEAL3	323	208					50			64						77												5
TMEM132E	316						170			35				145					290									5
VILL	126	2				34			215					256														5
WDR35															301						319	102	99		151			5
ZFP2				301	373						22										144		325					5
ZNF135	134		257							189	14											96						5
ZNF354C				384			357				304	249											280					5
ZNF423						32	121		181	375					139													5
ZNF662		100				107				329												322			173			5

The rank numbers of each negative gene in different cancer types are shown.
