# Supplementary material for: Pan-cancer analysis reveals synergistic effects of CDK4/6i and PARPi combination treatment in RB-proficient and RB-deficient breast cancer cells
Source: Cell Death Dis. 2020 Apr 6;11(4):219. doi: 10.1038/s41419-020-2408-1 (PMC7136254; doi:10.1038/s41419-020-2408-1)
Supplement: Supplementary file 11 — Table S6-17 [file 41419_2020_2408_MOESM11_ESM.pdf]

table S6. Pathway enrichment analysis of the top 400 genes showing positive correlation with all mutation loads in every single type of cancers.

Pathway	AML	ACC	BLCA	LGG	BRCA	COAD	ESCA	GBM	HNSC	KICH	KIRC	KIRP	LIHC	LUAD	LUSC	DLBC	OV	PAAD	PRAD	SARC	SKCM	STAD	TGCT	THCA	UCS	UCEC	UVM
2_Oxocarboxylic_acid_metabolism											6.3E-04																
Alcoholism			6.9E-03																								
Alzheimer_disease											1.0E-07		2.3E-03			2.7E-10		1.2E-03				5.1E-06		6.9E-08			
Amino_sugar_and_nucleotide_sugar_metabolism																		3.4E-02								1.6E-02	
Aminoacyl_tRNA_biosynthesis													2.1E-02						8.0E-03			2.6E-03		1.6E-03			
AMPK_signaling_pathway								2.9E-03																			
Antigen_processing_and_presentation			1.5E-02																								
Arachidonic_acid_metabolism									4.2E-02																		
Arrhythmogenic_right_ventricular_cardiomyopathy																					3.2E-02						
Axon_guidance																		4.4E-02									
Basal_transcription_factors																						3.8E-02				1.3E-02	
Base_excision_repair		7.2E-04		2.6E-02						6.9E-03																	
Biosynthesis_of_amino_acids					2.7E-03						6.8E-05																
Biosynthesis_of_antibiotics					2.0E-02				1.4E-02		7.7E-07	2.9E-02				1.8E-02						3.4E-04		1.0E-05		1.1E-02	7.7E-03
Bladder_cancer								4.8E-02																			
Carbon_metabolism					2.3E-03				3.2E-02		8.6E-05					1.5E-02						8.2E-05		1.8E-04			
Cardiac_muscle_contraction																1.2E-03								4.7E-02			
Cell_cycle		1.2E-14		1.2E-14	1.2E-14					8.2E-03				1.2E-14	2.6E-06		1.9E-03	1.2E-03	7.1E-08	1.2E-14		4.1E-13			3.7E-02	3.7E-02	
Central_carbon_metabolism_in_cancer								3.8E-02																			
Chagas_disease_American_trypanosomiasis						1.2E-02																					
Chemical_carcinogenesis							2.5E-02																				
Chemokine_signaling_pathway						3.3E-02																					
Citrate_cycle											6.9E-05											9.8E-03		9.6E-06			
Collecting_duct_acid_secretion																								3.3E-02			
Colorectal_cancer																			3.0E-02						1.9E-02		
Cysteine_and_methionine_metabolism											2.1E-03			9.5E-03								2.2E-02					
Cytokine_cytokine_receptor_interaction						2.1E-02											2.0E-03										
Cytosolic_DNA_sensing_pathway																									5.2E-03		
DNA_replication		1.1E-05	2.3E-03	4.5E-11	4.0E-11					1.5E-05				1.1E-05	4.3E-02					1.0E-07	2.3E-02				2.4E-04	2.4E-04	
Drug_metabolism_cytochrome_P450							1.3E-02					2.9E-02															
Drug_metabolism_other_enzymes												4.4E-02															
ECM_receptor_interaction								7.6E-03																			
Endocytosis																					1.2E-02						4.6E-02
Epithelial_cell_signaling_in_Helicobacter_pylori_infection																								8.1E-03			
Epstein_Barr_virus_infection					9.4E-03					3.3E-02			2.0E-03							3.7E-02		4.5E-02					
Fanconi_anemia_pathway		6.1E-03		5.9E-08	3.7E-04									6.1E-03				2.2E-03		2.3E-04	1.2E-02						
Fat_digestion_and_absorption							1.3E-04																				
Fatty_acid_elongation																								2.7E-02			
Fatty_acid_metabolism												4.9E-02															
Focal_adhesion								2.2E-02																			
FoxO_signaling_pathway						4.0E-02																					
Fructose_and_mannose_metabolism							2.8E-02		2.9E-02									4.8E-02									
Galactose_metabolism							3.2E-03																				
Glyoxylate_and_dicarboxylate_metabolism											3.8E-03											4.0E-02		6.0E-04			
Hepatitis_B					1.2E-02																				3.0E-02		
Hepatitis_C																									1.9E-02		
Herpes_simplex_infection																									1.7E-02		
HIF_1_signaling_pathway								4.6E-02																			
Homologous_recombination		2.6E-02		2.4E-09	6.0E-03									3.6E-03				6.0E-03		4.9E-05							
HTLV_I_infection		1.5E-03		2.9E-02	2.3E-03																	3.1E-02					
Huntington_disease					2.5E-02						3.6E-06		6.4E-03			3.4E-12		7.3E-06				1.9E-06		2.4E-08			
Hypertrophic_cardiomyopathy_(HCM)																					4.3E-02						
Influenza_A						2.8E-03																					
Leishmaniasis						2.5E-04																					
Lysine_degradation		2.8E-02																									
Lysosome																									1.1E-02		
Maturity_onset_diabetes_of_the_young							1.6E-04																				
Metabolic_pathways							3.2E-06		1.3E-03		7.5E-12	3.1E-06	2.4E-04			8.1E-04						6.9E-03		6.1E-12	4.6E-02	4.6E-02	
Metabolism_of_xenobiotics_by_cytochrome_P450							1.8E-02																				
Mineral_absorption						4.4E-03																			2.8E-02		
Mismatch_repair			2.2E-02	1.8E-07	2.0E-02									1.5E-03	1.3E-02					1.9E-03							
mRNA_surveillance_pathway																			7.4E-03								
Mucin_type_O_Glycan_biosynthesis							2.6E-02																				
Natural_killer_cell_mediated_cytotoxicity						8.3E-03																					
N_Glycan_biosynthesis			3.9E-02																								
Nicotine_addiction									9.7E-03																		
Non_alcoholic_fatty_liver_disease											1.6E-05		2.9E-02			2.9E-11		4.6E-04				1.2E-03		6.9E-08	3.6E-02		
Nucleotide_excision_repair				2.2E-03							2.7E-02									2.5E-02	4.5E-02				3.0E-02	3.0E-02	
One_carbon_pool_by_folate																						1.8E-02					
Oocyte_meiosis		2.6E-05		8.5E-06	9.2E-08									4.4E-06	1.2E-04				4.5E-03	1.5E-06		1.2E-02					
Osteoclast_differentiation						1.2E-05																					
Oxidative_phosphorylation											1.1E-07		7.7E-05			2.1E-13		6.7E-06				1.8E-07		1.2E-14			
p53_signaling_pathway		1.6E-02		4.5E-05	6.6E-06									1.6E-02				2.8E-02	8.6E-03	4.9E-03		2.8E-03					
Parkinson's_disease					2.9E-02						2.8E-07		7.0E-03			5.8E-11		2.6E-04				1.6E-08		6.3E-10			
Pentose_and_glucuronate_interconversions												3.4E-04															
Peroxisome												1.1E-05														2.6E-02	
Phagosome			4.9E-02																					4.9E-02	3.9E-02		
Phosphatidylinositol_signaling_system																										1.4E-02	
PI3K_Akt_signaling_pathway								2.0E-02																			
PPAR_signaling_pathway							4.9E-02																				
Progesterone_mediated_oocyte_maturation		1.2E-04		6.9E-06	3.6E-04									3.0E-03	1.1E-04				2.4E-02	5.7E-06		7.8E-04					
Prolactin_signaling_pathway						2.6E-02																					
Proteasome					1.3E-05								4.3E-09			2.2E-04	1.7E-02			3.6E-03		2.6E-08			2.4E-02		
Protein_export																						3.7E-03					
Protein_processing_in_endoplasmic_reticulum			4.9E-03															4.0E-03									
Purine_metabolism																			1.8E-03						3.4E-02		
Pyrimidine_metabolism		8.0E-03	5.9E-03	4.3E-03						3.4E-02				8.0E-03	7.5E-03				1.4E-02			2.9E-02			1.2E-03	1.2E-03	
Pyruvate_metabolism																						2.6E-02					
Renal_cell_carcinoma								9.4E-03																			
Retinol_metabolism												2.5E-02															
Ribosome					2.3E-02					1.2E-14	1.7E-02		4.1E-06		9.6E-03	1.2E-14						1.5E-02		8.0E-08			
Ribosome_biogenesis_in_eukaryotes	2.9E-04			2.9E-04					2.3E-03				1.8E-02	1.2E-04													
RNA_degradation	1.4E-04																		1.5E-02								
RNA_polymerase													4.1E-02														
RNA_transport	1.4E-02	3.7E-02		3.0E-05	1.3E-02					4.7E-02			2.8E-03	1.4E-03						7.6E-03		9.9E-03					
Salmonella_infection																									1.8E-02	1.8E-02	
Serotonergic_synapse									3.0E-02																		
Signaling_pathways_regulating_pluripotency_of_stem_cells		3.5E-02																									
Small_cell_lung_cancer					2.1E-02																						
Spliceosome		2.8E-02	1.8E-04		6.9E-03					3.5E-02			4.6E-03		8.5E-03			2.1E-02									
Staphylococcus_aureus_infection						8.8E-03																					
Steroid_biosynthesis																		1.4E-02									
Steroid_hormone_biosynthesis									7.3E-03																		
Synaptic_vesicle_cycle																								2.5E-02	4.8E-03		
Systemic_lupus_erythematosus			7.7E-04																								
Tight_junction							7.3E-03																				8.3E-03
TNF_signaling_pathway						1.3E-02																					
Toll_like_receptor_signaling_pathway						1.3E-02											3.2E-02										
Transcriptional_misregulation_in_cancer																	3.6E-02										
Tuberculosis						9.2E-03																					
Ubiquitin_mediated_proteolysis															1.0E-02				4.5E-02								
Valine_leucine_and_isoleucine_degradation											5.4E-03	8.4E-03												3.4E-02		1.5E-02	
Vibrio_cholerae_infection																								2.5E-03			
Viral_carcinogenesis					3.7E-02																	3.2E-02					

P values of each pathway with p value < 0.05 in every single cancer type are shown.

table S7. Pathway enrichment analysis of the top 400 genes showing negative correlation with all mutation loads.

Pathway	AML	ACC	BLCA	LGG	BRCA	COAD	ESCA	GBM	HNSC	KICH	KIRC	KIRP	LIHC	LUAD	LUSC	DLBC	OV	PAAD	PRAD	SARC	SKCM	STAD	TGCT	THCA	UCS	UCEC	UVM
ABC_transporters					4.80E-02													4.00E-02									
Adipocytokine_signaling_pathway																		3.30E-02									
Adrenergic_signaling_in_cardiomyocytes				1.80E-03	1.90E-02				4.00E-04		2.80E-02	2.00E-02		3.20E-02				9.40E-04			2.60E-02						
Aldosterone_synthesis_and_secretion											2.40E-02	2.60E-03						1.30E-02									
Alzheimer_disease	2.29E-02																									2.10E-02	
Amoebiasis													1.80E-05														
Amphetamine_addiction				1.70E-02							4.40E-02																
Apoptosis																								7.90E-03			
Arrhythmogenic_right_ventricular_cardiomyopathy									2.10E-03			5.80E-03			4.10E-02												
Ascorbate_and_aldarate_metabolism		2.10E-02								2.30E-02																	
Axon_guidance													2.10E-02		8.70E-03							4.50E-02			1.40E-02		
B_cell_receptor_signaling_pathway																								4.40E-04			
Bacterial_invasion_of_epithelial_cells	6.94E-03																										
Basal_cell_carcinoma							2.50E-04														7.10E-04						
beta_Alanine_metabolism								4.10E-03		3.30E-02																	
Bile_secretion																					4.80E-02						
Biosynthesis_of_amino_acids				1.40E-03																							
Biosynthesis_of_antibiotics	4.20E-02			3.30E-03				1.10E-05																			
Biosynthesis_of_unsaturated_fatty_acids				1.50E-02																							
Butanoate_metabolism										2.30E-02																	
Calcium_signaling_pathway											5.50E-07	1.50E-05	4.20E-02					3.80E-03									
cAMP_signaling_pathway				4.20E-05							1.70E-04	1.60E-02															
Carbon_metabolism	5.12E-03			2.40E-04				3.40E-02																			
Cardiac_muscle_contraction								2.20E-02	2.80E-03																		
Cell_adhesion_molecules															1.50E-02							6.00E-03					
cGMP_PKG_signaling_pathway											3.10E-08	1.20E-06	3.30E-03					9.10E-07			4.60E-02	1.40E-02					
Chagas_disease																							1.70E-02	2.30E-04			
Chemokine_signaling_pathway	1.84E-02								8.20E-03									4.90E-03					2.90E-03	3.40E-02			
Cholinergic_synapse											1.80E-03	4.10E-03						3.10E-03									
Chronic_myeloid_leukemia															4.30E-02												
Circadian_entrainment				3.00E-04					8.80E-03		3.00E-03							2.40E-02									
Cocaine_addiction				2.50E-02																							
Complement_and_coagulation_cascades														1.40E-02													
Cytokine_cytokine_receptor_interaction																							7.80E-04				
Cytosolic_DNA_sensing_pathway																							5.70E-03				
Dilated_cardiomyopathy									4.80E-03		2.80E-04	4.50E-02	3.60E-02		4.30E-03												
Dopaminergic_synapse				8.80E-03							4.50E-02	7.30E-04						2.40E-02									
Drug_metabolism_cytochrome_P450		7.40E-04																	1.30E-03								
Drug_metabolism_other_enzymes		1.90E-02																									
ECM_receptor_interaction													4.20E-09		2.80E-05							4.40E-02					
Endocytosis	3.04E-03											2.50E-03				6.90E-03											
Epithelial_cell_signaling_in_Helicobacter_pylori_infection																								4.50E-02			
Epstein_Barr_virus_infection	4.74E-02																							1.40E-02			
ErbB_signaling_pathway			2.60E-03													4.70E-02								1.80E-03	4.70E-02		
Estrogen_signaling_pathway	2.37E-02											2.70E-02									4.60E-02						
Fat_digestion_and_absorption						3.60E-02																					
Fatty_acid_degradation		1.60E-02																									
Fatty_acid_metabolism				4.50E-03																							
Fc_epsilon_RI_signaling_pathway																							3.00E-02	4.70E-02			
Fc_gamma_R_mediated_phagocytosis	1.35E-04																										
Focal_adhesion					3.80E-02				1.60E-03		2.60E-04	2.90E-03	7.40E-11		3.80E-04												
FoxO_signaling_pathway																4.80E-03		2.90E-02									
GABAergic_synapse				1.30E-02							7.30E-03		3.80E-02														
Gap_junction											3.30E-02	1.60E-02	4.30E-02								3.00E-02						
Gastric_acid_secretion												2.70E-02															
Glutamatergic_synapse				1.10E-03							2.80E-02							3.60E-03									
Glycerophospholipid_metabolism								1.60E-02																			
Glycine_serine_and_threonine_metabolism				1.20E-02																							
Glycolysis_Gluconeogenesis	4.32E-02			1.80E-02																							
Glycosaminoglycan_biosynthesis_chondroitin_sulfate_dermatan_sulfate							4.00E-03						8.10E-04														
Glycosphingolipid_biosynthesis_ganglio_series									3.10E-02																		
GnRH_signaling_pathway	4.68E-02																										
Hedgehog_signaling_pathway														1.50E-05													
Hematopoietic_cell_lineage																							5.90E-03				
Hepatitis_B			3.70E-02														3.00E-02										
Herpes_simplex_infection																							1.90E-02	3.10E-02			
Hippo_signaling_pathway							3.20E-03										3.70E-02				1.00E-02						
Huntington_disease								2.70E-03																		4.30E-03	
Hypertrophic_cardiomyopathy									3.40E-03		4.80E-03	2.10E-03			3.00E-03										3.30E-02		
Inflammatory_bowel_disease																								3.90E-02			
Inflammatory_mediator_regulation_of_TRP_channels				2.40E-02		3.70E-02												7.00E-03									
Influenza_A	2.84E-02																						4.90E-03	2.60E-03			
Inositol_phosphate_metabolism				2.30E-02																							
Insulin_resistance												1.20E-02															
Insulin_secretion				4.50E-02							7.30E-03	4.70E-02															
Insulin_signaling_pathway	4.53E-02		2.90E-02																						5.70E-03		
Jak_STAT_signaling_pathway																								2.70E-02			
Legionellosis	4.32E-03																						2.40E-03				
Leukocyte_transendothelial_migration	2.02E-02																										
Long_term_depression				1.20E-02								1.20E-02															
Long_term_potentiation				1.70E-02										4.80E-02													
Lysosome	5.17E-08																						3.50E-02				
MAPK_signaling_pathway											3.40E-02													1.30E-02	4.60E-02	4.60E-02	
Melanogenesis																			3.20E-02		1.40E-02						
Melanoma													1.90E-02		4.10E-02												
Metabolic_pathways				6.00E-03				6.50E-07		5.30E-04																	
Metabolism_of_xenobiotics_by_cytochrome_P450																			1.00E-02								
Morphine_addiction				4.60E-03					2.80E-02		2.40E-03		4.80E-02														
mRNA_surveillance_pathway																1.40E-02											
Mucin_type_O_Glycan_biosynthesis														2.60E-02	1.90E-02												
Natural_killer_cell_mediated_cytotoxicity	8.39E-03																						1.30E-02	1.10E-02			
Neuroactive_ligand_receptor_interaction				1.60E-03														8.50E-03									
Neurotrophin_signaling_pathway																								3.40E-02			
NF_kappa_B_signaling_pathway																							1.80E-06	1.10E-06			
Nicotine_addiction				1.30E-02																							
NOD_like_receptor_signaling_pathway																							2.70E-03				
Non_alcoholic_fatty_liver_disease								1.60E-02																3.20E-02		3.40E-03	
Notch_signaling_pathway																					1.50E-02						
Oocyte_meiosis					4.10E-03																						
Osteoclast_differentiation	9.19E-07																						2.70E-05	1.20E-03			
Other_types_of_O_glycan_biosynthesis							1.40E-02																				
Oxidative_phosphorylation	1.43E-02							5.30E-04																		6.20E-03	
Oxytocin_signaling_pathway									9.60E-03		3.40E-04	1.00E-03						6.00E-03			4.00E-03						
p53_signaling_pathway																				2.50E-02							
Parkinson_disease	2.11E-02							8.90E-04																		8.80E-03	
Pathogenic_Escherichia_coli_infection																							4.10E-02				
Pathways_in_cancer					2.50E-03		2.60E-02		3.30E-02		1.10E-04	1.90E-02	3.70E-06		5.40E-03			4.10E-04			1.90E-03						
Pentose_and_glucuronate_interconversions	4.96E-02									1.20E-03																	
Peroxisome						9.00E-04																					2.60E-02
Pertussis	5.60E-03																						4.30E-02				
PI3K_Akt_signaling_pathway											9.90E-03	2.90E-02	1.40E-06		1.10E-02												
Platelet_activation	3.36E-02										2.80E-04	8.20E-04	5.20E-04					2.50E-02									
Porphyrin_and_chlorophyll_metabolism										1.50E-02																	
Progesterone_mediated_oocyte_maturation					2.30E-02																						
Prostate_cancer												1.60E-02															
Protein_digestion_and_absorption													4.50E-08														
Proteoglycans_in_cancer							4.50E-02						1.40E-03					7.80E-03									
Rap1_signaling_pathway											3.10E-04	2.40E-02	4.00E-02					3.40E-03			2.90E-03						
Ras_signaling_pathway									2.60E-02		1.60E-02		3.80E-03					1.60E-02						3.80E-02			
Regulation_of_actin_cytoskeleton	7.72E-03								4.50E-02		3.60E-03																
Renin_secretion											9.30E-03	1.00E-04						1.10E-04			3.80E-02						
Renin_angiotensin_system																		7.00E-03									
Retrograde_endocannabinoid_signaling				2.10E-03								2.90E-02						3.10E-02									
Rheumatoid_arthritis																							3.90E-04				
Ribosome																	2.20E-02				1.40E-03						
RIG_I_like_receptor_signaling_pathway																							3.30E-02				
RNA_degradation																3.20E-02				3.90E-02							
Salivary_secretion												4.90E-02		8.80E-03				3.70E-03									
Salmonella_infection	5.91E-04																						1.20E-03				
Serotonergic_synapse																		4.30E-02									
Shigellosis	4.56E-04																						2.40E-02				
Signaling_pathways_regulating_pluripotency_of_stem_cells							2.70E-02																				
Small_cell_lung_cancer													1.00E-02		4.60E-03		3.10E-02										
Sphingolipid_metabolism																		4.80E-02									
Steroid_biosynthesis								8.40E-03																			
Sulfur_relay_system						1.40E-02																				1.10E-02	
T_cell_receptor_signaling_pathway																							1.20E-03	1.40E-08			
TGF_beta_signaling_pathway													9.90E-03			9.90E-03											
Thyroid_hormone_signaling_pathway			3.10E-03									4.80E-03									2.50E-02						
Thyroid_hormone_synthesis																					1.20E-02						
Tight_junction									4.40E-02																		
TNF_signaling_pathway															1.30E-02								7.70E-05	1.30E-03			
Toll_like_receptor_signaling_pathway																							3.60E-04	2.00E-02			
Toxoplasmosis															2.10E-02												
Transcriptional_misregulation_in_cancer																							1.20E-03	2.00E-02			
Tuberculosis	1.19E-05																						3.80E-02				
Type_II_diabetes_mellitus																								1.50E-02			
Ubiquitin_mediated_proteolysis																2.00E-02											2.10E-02
Valine,_leucine_and_isoleucine_degradation				2.20E-02						4.10E-03																	
Vascular_smooth_muscle_contraction											6.90E-07	1.10E-08	4.60E-02					1.00E-03			9.20E-03	3.60E-02					
Wnt_signaling_pathway							2.50E-02														8.40E-05				2.10E-02	2.10E-02	
Leishmaniasis	3.13E-05																										
Phagosome	4.39E-04																										
Staphylococcus_aureus_infection	8.31E-04																										

P values of each pathway with p value < 0.05 in every single cancer type are shown.

table S8. Pathway enrichment analysis of the top 200 genes showing positive correlation with all mutation loads.

Pathway	AML	ACC	BLCA	LGG	BRCA	COAD	ESCA	GBM	HNSC	KICH	KIRC	KIRP	LIHC	LUAD	LUSC	DLBC	OV	PAAD	PRAD	SARC	SKCM	STAD	TGCT	THCA	UCS	UCEC	UVM
Alcoholism			1.30E-02																								
Alzheimer_disease											1.00E-03					2.40E-03		4.70E-02				1.50E-06		1.30E-06			
Aminoacyl-tRNA_biosynthesis													7.90E-03														
AMPK_signaling_pathway							3.60E-02	1.90E-03																			
Antigen_processing_and_presentation			2.20E-02																								
Ascorbate_and_aldarate_metabolism												2.20E-02															
Base_excision_repair		4.30E-04																									
Biosynthesis_of_antibiotics												9.00E-03				2.80E-03						4.40E-02		5.80E-03			
Carbon_metabolism					4.30E-02											2.10E-02						2.90E-02		1.50E-02			
Cardiac_muscle_contraction															4.80E-02									1.60E-02			
Cell_cycle		3.11E-12		1.23E-14	1.23E-14									6.40E-11	5.30E-05		1.20E-02	1.50E-02	2.60E-06	4.12E-13		4.50E-09					
Central_carbon_metabolism_in_cancer								3.10E-02																			
Citrate_cycle																								5.50E-04			
Colorectal_cancer														1.90E-02											4.90E-02		
DNA_replication				9.40E-07	5.50E-05					1.10E-02				4.30E-02						7.80E-03					1.20E-02	1.20E-02	
Epstein-Barr_virus_infection													4.20E-04					2.40E-02		2.10E-02							
Fanconi_anemia_pathway				5.70E-07	2.40E-02									1.30E-02				3.30E-03		3.20E-04							
Fat_digestion_and_absorption							7.30E-03																				
Focal_adhesion								6.30E-03																			
FoxO_signaling_pathway								1.40E-02																			
Fructose_and_mannose_metabolism							4.20E-02																				
GABAergic_synapse									8.20E-03																		
Glycerolipid_metabolism												1.30E-02															
Glycolysis_/_Gluconeogenesis					4.40E-02							1.90E-02															
Glyoxylate_and_dicarboxylate_metabolism																								4.70E-02			
Herpes_simplex_infection																									1.10E-02		
HIF-1_signaling_pathway								2.10E-02																			
Homologous_recombination				1.50E-04	4.60E-03								4.70E-02	2.30E-03				4.60E-03		4.20E-03							
HTLV-I_infection		9.80E-05																									
Huntington's_disease											5.40E-04					1.40E-05		1.70E-03				1.10E-06		5.50E-06			
Inositol_phosphate_metabolism																										3.60E-02	
Insulin_resistance																										4.90E-03	
Lysine_degradation								1.80E-02																			
Metabolic_pathways							1.20E-04				7.90E-05	9.40E-05	3.00E-02			9.00E-03						4.70E-03		1.20E-06			
MicroRNAs_in_cancer					1.80E-02																						
Mineral_absorption						2.30E-03																					
Mismatch_repair			4.00E-03	1.30E-03	2.90E-02									1.20E-03											3.70E-02		
Morphine_addiction									1.00E-02																		
Nicotine_addiction									6.10E-03																		
Non-alcoholic_fatty_liver_disease											2.50E-03					1.20E-03						8.30E-03		5.20E-08			
Non-small_cell_lung_cancer										3.40E-02																	
Nucleotide_excision_repair											2.00E-02														2.40E-02		
One_carbon_pool_by_folate																						3.70E-02					
Oocyte_meiosis		2.60E-06		5.70E-04	4.40E-08									4.80E-04	1.60E-04				4.00E-03	2.30E-04		2.60E-02					
Other_glycan_degradation											2.00E-02																
Oxidative_phosphorylation											2.10E-04					9.40E-05		4.50E-03				9.60E-08		1.10E-09			
p53_signaling_pathway		8.00E-04		3.70E-03	1.10E-03													4.40E-02		4.10E-02		3.50E-03					
Parkinson_disease											3.30E-04					8.10E-04		2.50E-02				2.10E-07		1.70E-06			
Pentose_and_glucuronate_interconversions												2.30E-04															
Pentose_phosphate_pathway									3.00E-02																		
Peroxisome												5.30E-03															
Phosphatidylinositol_signaling_system																										1.80E-02	
PI3K-Akt_signaling_pathway								1.00E-03																			
Progesterone-mediated_oocyte_maturation		4.30E-05		1.70E-04	6.50E-07										3.40E-04				9.80E-03	5.70E-06		2.10E-03					
Proteasome													7.50E-07							1.50E-03		3.90E-06					
Protein_processing_in_endoplasmic_reticulum			9.90E-03																								
Pyrimidine_metabolism										4.30E-02					2.60E-02										4.90E-02		
Renal_cell_carcinoma								5.00E-03																			
Retrograde_endocannabinoid_signaling									1.50E-02																		
Ribosome										1.23E-14			5.70E-03			4.30E-11								8.00E-03			
Ribosome_biogenesis_in_eukaryotes	2.47E-02			9.30E-03					4.80E-02				2.00E-02	4.60E-02													
RNA_transport	7.73E-03			2.10E-04							1.90E-02		4.40E-03														4.90E-02
Spliceosome			1.10E-02																								
Starch_and_sucrose_metabolism																										4.50E-02	
Steroid_biosynthesis			3.20E-02															1.50E-03									
Systemic_lupus_erythematosus			2.80E-03																								
Ubiquitin_mediated_proteolysis																				2.00E-02							
Valine_leucine_and_isoleucine_degradation											2.00E-02																
VEGF_signaling_pathway								2.70E-02																			
RNA_degradation	1.79E-02																										

P values of each pathway with p value < 0.05 in every single cancer type are shown.

table S9. Pathway enrichment analysis of the top 200 genes showing negative correlation with all mutation loads.

Pathway	AML	ACC	BLCA	LGG	BRCA	COAD	ESCA	GBM	HNSC	KICH	KIRC	KIRP	LIHC	LUAD	LUSC	DLBC	OV	PAAD	PRAD	SARC	SKCM	STAD	TGCT	THCA	UCS	UCEC	UVM
Acute_myeloid_leukemia																							3.40E-02				
Adrenergic_signaling_in_cardiomyocytes				3.80E-02	1.20E-02													1.30E-03			4.40E-02						
Aldosterone_synthesis_and_secretion												1.10E-02						3.00E-02				2.50E-02					
Amoebiasis													2.30E-05														
Ascorbate_and_aldarate_metabolism										4.00E-02																	
Axon_guidance															3.40E-02												
Bacterial_invasion_of_epithelial_cells	5.04E-03								4.10E-02																		
Basal_cell_carcinoma							1.00E-02																				
Basal_transcription_factors																4.20E-02											
beta_Alanine_metabolism								4.00E-03																			
Bile_secretion										4.80E-02											2.50E-02						
Biosynthesis_of_antibiotics	1.08E-02							1.50E-06																			
Butanoate_metabolism										4.00E-02																	
Calcium_signaling_pathway											4.60E-07	1.10E-04						1.70E-02									
cAMP_signaling_pathway				3.60E-03														6.00E-03									
Carbon_metabolism	5.47E-03			1.40E-02				6.30E-03																			
Cardiac_muscle_contraction									3.70E-02																		
Cell_adhesion_molecules																						2.40E-02					
cGMP_PKG_signaling_pathway											1.60E-06	1.90E-03						4.40E-04				4.00E-02					
Chemokine_signaling_pathway			1.70E-02																				3.10E-02				
Cholinergic_synapse											3.30E-02	3.10E-02						1.40E-02									
Circadian_entrainment				3.30E-02								1.90E-02															
Citrate_cycle								3.90E-02																			
Complement_and_coagulation_cascades													4.30E-02	6.20E-03													
Dilated_cardiomyopathy															8.70E-03												
Dopaminergic_synapse												4.10E-04															
Drug_metabolism_cytochrome_P450		3.20E-02																	2.00E-02								
ECM_receptor_interaction													4.70E-07														
Endocytosis	1.09E-03																										
Epithelial_cell_signaling_in_Helicobacter_pylori_infection			3.80E-02																								
ErbB_signaling_pathway			1.60E-02															3.60E-02									
Estrogen_signaling_pathway												2.10E-02															
Fc_gamma_R_mediated_phagocytosis	3.20E-02																										
Focal_adhesion											7.20E-03		4.00E-07														
GABAergic_synapse											1.40E-02																
Gap_junction												1.40E-02															
Glucagon_signaling_pathway				3.70E-02																							
Glutamatergic_synapse												3.30E-02															
Glycosaminoglycan_biosynthesis_chondroitin_sulfate_dermatan_sulfate													2.10E-02														
Glycosphingolipid_biosynthesis_lacto_and_neolacto_series									2.70E-02				3.40E-02														
Hedgehog_signaling_pathway														2.90E-03													
Hepatitis_B			2.20E-02																								
Hippo_signaling_pathway																	2.20E-02										
Histidine_metabolism														2.50E-02													
HTLV_I_infection																								8.30E-03			
Hypertrophic_cardiomyopathy															6.70E-03												
Inflammatory_mediator_regulation_of_TRP_channels				3.60E-02								2.10E-02						9.00E-03									
Influenza_A	3.83E-02		1.30E-02																								
Inositol_phosphate_metabolism											4.30E-02																
Insulin_resistance																	2.80E-02										
Insulin_signaling_pathway																									3.00E-03		
Jak_STAT_signaling_pathway																				2.00E-02				1.00E-02			
Long_term_depression												3.80E-03															
Long_term_potentiation												3.30E-02															
Lysosome	7.82E-06																										
Malaria																								9.90E-03			
MAPK_signaling_pathway											6.60E-03																
Metabolic_pathways	2.18E-02			8.90E-03				5.30E-05		6.90E-03							3.10E-02										
Morphine_addiction				2.80E-02							3.10E-03																
mRNA_surveillance_pathway																2.80E-02											
Mucin_type_O_Glycan_biosynthesis									3.70E-02																		3.50E-02
NF_kappa_B_signaling_pathway																								4.50E-02			
Nicotinate_and_nicotinamide_metabolism									3.30E-02																		
Oocyte_meiosis					1.90E-02																						
Osteoclast_differentiation	1.10E-02																						1.30E-03				
Other_types_of_O_glycan_biosynthesis							2.70E-02																				
Oxidative_phosphorylation	4.17E-02							4.90E-02																			
Oxytocin_signaling_pathway			3.10E-02								1.70E-03	2.70E-02						4.30E-02			3.00E-03						
p53_signaling_pathway															2.70E-02												
Pathways_in_cancer					1.10E-02				3.80E-02		7.90E-05		3.20E-02					5.10E-03									
Pentose_and_glucuronate_interconversions										8.10E-04																	
Peroxisome						4.00E-02																					
Phosphatidylinositol_signaling_system												2.10E-02															
PI3K_Akt_signaling_pathway													4.40E-04														
Platelet_activation	3.83E-02										1.40E-02	2.60E-03															
Porphyrin_and_chlorophyll_metabolism										1.30E-02																	
Protein_digestion_and_absorption													5.70E-06														
Proteoglycans_in_cancer																		6.30E-03									
Proximal_tubule_bicarbonate_reclamation																					1.80E-02						
Rap1_signaling_pathway											8.00E-03	2.40E-02															
Ras_signaling_pathway											1.20E-02							1.10E-02									
Regulation_of_actin_cytoskeleton											8.20E-03																
Regulation_of_autophagy																			4.30E-02								
Renin_secretion												5.70E-04						1.60E-02									
Renin_angiotensin_system																		1.60E-02									
Retinol_metabolism										4.20E-02																	
Retrograde_endocannabinoid_signaling				3.90E-02							2.50E-02	4.40E-03															
Salmonella_infection	1.71E-04																										
Serotonergic_synapse												3.10E-02															
Signaling_pathways_regulating_pluripotency_of_stem_cells																								3.80E-02			
Small_cell_lung_cancer													1.50E-02														
Terpenoid_backbone_biosynthesis								2.20E-02																			
TGF_beta_signaling_pathway																								4.10E-02			
Thyroid_hormone_signaling_pathway			8.60E-03																		2.00E-02						
Thyroid_hormone_synthesis																					3.70E-03						
TNF_signaling_pathway																							1.10E-02				
Transcriptional_misregulation_in_cancer																							2.00E-02				
Valine,_leucine_and_isoleucine_degradation								1.30E-02																			
Vascular_smooth_muscle_contraction											4.30E-05	1.60E-03										1.40E-02					
Vibrio_cholerae_infection	3.99E-02																										
Wnt_signaling_pathway				8.10E-03			2.70E-02														3.70E-02						
Drug_metabolism_other_enzymes	2.78E-02																										
Glycosaminoglycan_degradation	2.97E-02																										
Legionellosis	4.18E-02																										
Leishmaniasis	1.85E-02																										
Natural_killer_cell_mediated_cytotoxicity	7.89E-03																										
Pentose_phosphate_pathway	7.96E-03																										
Shigellosis	2.85E-04																										
Toxoplasmosis	2.68E-02																										
Tuberculosis	4.11E-02																										

P values of each pathway with p value < 0.05 in every single cancer type are shown.

table S10. Pathway enrichment analysis of the top 400 genes showing positive correlated with missense mutation loads.

Pathway	AML	ACC	BLCA	LGG	BRCA	COAD	ESCA	GBM	HNSC	KICH	KIRC	KIRP	LIHC	LUAD	LUSC	DLBC	OV	PAAD	PRAD	SARC	SKCM	STAD	TGCT	THCA	UCS	UCEC	UVM
2_Oxocarboxylic_acid_metabolism											6.00E-04																
Alcoholism			6.60E-03																								
Alzheimer_disease											4.70E-05		1.80E-02	3.10E-03		3.70E-08		2.80E-03				6.20E-06		3.10E-10			
Amino_sugar_and_nucleotide_sugar_metabolism																		3.00E-02							3.00E-02	1.80E-03	
Aminoacyl_tRNA_biosynthesis											2.10E-02		2.00E-02						9.70E-03			2.80E-03		7.30E-03			
AMPK_signaling_pathway								3.40E-03																			
Antigen_processing_and_presentation			1.40E-02			3.00E-02																					
Apoptosis																										2.70E-02	
Arachidonic_acid_metabolism									4.10E-02																		
Arrhythmogenic_right_ventricular_cardiomyopathy																					3.10E-02						
Axon_guidance																		3.70E-02									
Bacterial_invasion_of_epithelial_cells														6.30E-03											4.30E-02		
Basal_transcription_factors																						4.00E-02					
Base_excision_repair		8.90E-05		4.40E-03	4.90E-02					6.10E-03										4.50E-02	1.70E-02				4.70E-02		
Biosynthesis_of_amino_acids											6.20E-05																
Biosynthesis_of_antibiotics											2.80E-06	1.00E-02				1.60E-02						1.20E-03		4.20E-04		4.30E-02	
Carbon_metabolism					2.40E-02						1.50E-05					1.40E-02						9.40E-05		2.70E-03			
Cardiac_muscle_contraction																1.80E-02								4.60E-02			
Cell_cycle		3.70E-15		2.00E-23	1.40E-25					1.50E-17					1.50E-05		3.60E-03	1.10E-02	1.70E-08	9.10E-17		4.80E-12					
Central_carbon_metabolism_in_cancer								4.10E-02																			
Chagas_disease_(American_trypanosomiasis)						9.50E-03																					
Citrate_cycle											6.60E-04											1.00E-02		1.00E-04			
Colorectal_cancer																			3.40E-02								
Cysteine_and_methionine_metabolism											2.00E-03											2.30E-02					
Cytokine_cytokine_receptor_interaction						1.60E-02																					
Cytosolic_DNA_sensing_pathway																									4.70E-03		
DNA_replication		1.50E-04	1.40E-02	1.00E-13	3.30E-11					1.30E-05					4.30E-02					8.10E-09	2.90E-03				1.70E-03		
Drug_metabolism__other_enzymes												4.30E-02															
Endocytosis							4.80E-02							1.20E-03							1.10E-02						9.10E-03
Epithelial_cell_signaling_in_Helicobacter_pylori_infection																									2.40E-02		
Epstein_Barr_virus_infection					2.90E-03								1.60E-04				1.50E-02			1.70E-02		2.20E-02					
Fanconi_anemia_pathway		7.10E-03		9.00E-08	3.30E-04					6.60E-03								4.10E-02		2.70E-04	1.20E-02						
Fat_digestion_and_absorption							1.50E-04																				
Fatty_acid_elongation																								2.70E-02			
Fatty_acid_metabolism												4.80E-02															
FoxO_signaling_pathway						3.40E-02																					
Fructose_and_mannose_metabolism							3.00E-02		2.90E-02									4.30E-02									
Galactose_metabolism							2.50E-02																				
Gap_junction										4.80E-02																	
Glycosphingolipid_biosynthesis__lacto_and_neolacto_series							1.70E-02																				
Glyoxylate_and_dicarboxylate_metabolism											2.60E-02											7.10E-03		3.30E-02			
Hepatitis_B					1.10E-02					4.50E-02																	
Hepatitis_C																									1.70E-02		
Herpes_simplex_infection																	3.20E-02								5.30E-03	6.70E-03	
HIF_1_signaling_pathway								3.70E-03																			
Hippo_signaling_pathway														4.30E-02													
Homologous_recombination		2.80E-02		3.60E-09	5.60E-03					3.80E-03							2.00E-02	5.20E-03		5.60E-05							
HTLV_I_infection		5.90E-03			1.90E-03					6.30E-04																	
Huntington_disease											5.30E-05		1.60E-02			5.30E-10		6.90E-05				2.40E-06		1.10E-07			
Hypertrophic_cardiomyopathy)																					4.20E-02						
Influenza_A						4.60E-02																					
Inositol_phosphate_metabolism																										9.90E-03	
Insulin_resistance																										5.00E-02	
Leishmaniasis						1.10E-03																					
Lysine_degradation		3.10E-02																									
Lysosome																									9.70E-03		
Maturity_onset_diabetes_of_the_young							1.70E-02																				
Measles																									4.50E-02		
Metabolic_pathways							1.80E-05		6.70E-03		1.30E-11	2.40E-06	6.70E-04			5.40E-04		8.10E-03				9.30E-03		2.00E-08	4.90E-02		
Mineral_absorption						3.80E-03																					
Mismatch_repair			2.10E-02	9.90E-09	1.90E-02					1.40E-02					1.30E-02					2.10E-03							
mRNA_surveillance_pathway														1.30E-02					2.10E-03								
Mucin_type_O_Glycan_biosynthesis							2.70E-02																				
N_Glycan_biosynthesis			3.90E-02	1.80E-02																							
Natural_killer_cell_mediated_cytotoxicity						6.70E-03																					
Neurotrophin_signaling_pathway														4.40E-02													
Nicotine_addiction									9.50E-03																		
Non_alcoholic_fatty_liver_disease_(NAFLD)											2.60E-04					5.90E-09	3.30E-02	1.20E-02				1.40E-03		6.30E-08			
Nucleotide_excision_repair			3.40E-02	3.60E-04						2.10E-02	2.60E-02									5.40E-03	4.40E-02						
One_carbon_pool_by_folate																						1.90E-02					
Oocyte_meiosis		3.70E-05		1.30E-05	7.30E-08					8.60E-07				2.20E-03	5.90E-04				2.90E-04	1.20E-05		1.30E-02					
Osteoclast_differentiation						7.90E-04																					
Other_types_of_O_glycan_biosynthesis										3.20E-02																	
Oxidative_phosphorylation											1.60E-05		4.20E-03			8.40E-11		2.10E-05				2.30E-07		3.10E-13			
p53_signaling_pathway		1.90E-02		6.10E-05	5.70E-06					3.90E-03									3.30E-04	2.30E-02		6.40E-04					
Pancreatic_cancer																									2.10E-02		
Parkinson_disease					2.70E-02						3.30E-05					1.20E-08		7.10E-04				2.00E-08		2.40E-08			
Pathogenic_Escherichia_coli_infection														2.30E-02													
Pentose_and_glucuronate_interconversions									3.90E-02			3.30E-04															
Peroxisome												7.70E-05															
Phagosome			4.70E-02																								
Phosphatidylinositol_signaling_system																										2.20E-03	
PI3K_Akt_signaling_pathway								2.30E-02																			
PPAR_signaling_pathway							1.40E-02																				
Primary_immunodeficiency																									5.00E-02		
Progesterone_mediated_oocyte_maturation		8.20E-04		1.40E-06	3.10E-04					4.70E-07					1.10E-04				7.40E-03	4.40E-05		8.70E-04					
Proteasome					1.10E-06								2.30E-10			2.50E-05	1.20E-02			6.20E-04		2.30E-09					
Protein_export																						3.90E-03					
Protein_processing_in_endoplasmic_reticulum			1.50E-03					2.20E-02						3.30E-03													
Purine_metabolism																			8.10E-03						2.90E-02		
Pyrimidine_metabolism			1.90E-02	5.40E-03	4.70E-02										7.50E-03					4.10E-02					3.90E-03		
Pyruvate_metabolism																						5.60E-03					
Regulation_of_actin_cytoskeleton														3.70E-02													
Renal_cell_carcinoma								4.30E-02																			
Ribosome										3.40E-07	4.50E-02		6.50E-07	2.60E-02	9.60E-03	2.20E-20						5.90E-03		5.90E-15			
Ribosome_biogenesis_in_eukaryotes	3.20E-07			3.90E-04					9.70E-03				4.40E-03														
RIG_I_like_receptor_signaling_pathway																									2.80E-02		
RNA_degradation	4.10E-04																		4.10E-03								
RNA_polymerase													6.60E-03														
RNA_transport	3.30E-02	1.70E-02		3.80E-07	1.10E-02								2.50E-03	7.30E-05						3.00E-03		2.70E-02	3.60E-02				
Salmonella_infection						4.10E-02																			4.20E-03		
Serotonergic_synapse									2.90E-02																		
Shigellosis														1.20E-02													
Small_cell_lung_cancer					2.00E-02					4.30E-02																	
Spliceosome		3.40E-03	4.10E-05										1.40E-02		8.50E-03		1.80E-02										
Staphylococcus_aureus_infection						3.40E-02																					
Steroid_biosynthesis																		1.20E-02									
Steroid_hormone_biosynthesis									1.30E-03																		
Synaptic_vesicle_cycle																									1.90E-02		
Systemic_lupus_erythematosus			7.40E-04																								
Terpenoid_backbone_biosynthesis														1.10E-02													
Tight_junction							6.40E-04							2.70E-02													
TNF_signaling_pathway																										1.40E-02	
Toll_like_receptor_signaling_pathway																	2.00E-02								1.50E-02		
Tuberculosis						7.20E-03																					
Ubiquitin_mediated_proteolysis										3.50E-02					1.00E-02												
Valine_leucine_and_isoleucine_degradation											5.10E-03	4.60E-02															
Vibrio_cholerae_infection			4.90E-02																								
Viral_carcinogenesis			1.80E-02		3.40E-02																	1.60E-02			2.90E-02		

P values of each pathway with p value < 0.05 in every single cancer type are shown.

table S11. Pathway enrichment analysis of the top 400 genes showing negative correlated with missense mutation loads.

Pathway	AML	ACC	BLCA	LGG	BRCA	COAD	ESCA	GBM	HNSC	KICH	KIRC	KIRP	LIHC	LUAD	LUSC	DLBC	OV	PAAD	PRAD	SARC	SKCM	STAD	TGCT	THCA	UCS	UCEC	UVM
ABC_transporters					5.00E-02													4.50E-02									
Adrenergic_signaling_in_cardiomyocytes				6.30E-03	1.70E-03				5.10E-04			1.80E-02						4.80E-03			2.70E-02						
Aldosterone_synthesis_and_secretion					1.80E-02							2.40E-03						6.10E-04									
Alzheimer_disease																										2.00E-03	
Amoebiasis													1.30E-05														
Amphetamine_addiction				1.80E-02																							
AMPK_signaling_pathway			5.00E-02																								
Amyotrophic_lateral_sclerosis																									4.80E-02		
Apoptosis																								4.00E-02			
Arrhythmogenic_right_ventricular_cardiomyopathy									1.20E-02		1.40E-02	2.30E-02			4.20E-02												
Ascorbate_and_aldarate_metabolism		2.30E-02																									
Axon_guidance													1.80E-02		3.00E-02							4.40E-02			1.70E-02		
B_cell_receptor_signaling_pathway																								1.40E-02			
Bacterial_invasion_of_epithelial_cells	1.90E-02																										
Basal_cell_carcinoma							2.40E-04														7.70E-04						
Bile_secretion																					5.00E-02						
Biosynthesis_of_amino_acids				1.50E-03																							
Biosynthesis_of_antibiotics	2.80E-02			3.40E-03				4.30E-06																			
Biosynthesis_of_unsaturated_fatty_acids				1.70E-03																							
Calcium_signaling_pathway										1.60E-02	2.90E-06	5.40E-05						5.00E-03						3.30E-02			
cAMP_signaling_pathway				4.60E-05	3.40E-02						2.20E-03							2.70E-02			4.60E-02						
Carbon_metabolism	3.40E-03			2.50E-04																							
Cardiac_muscle_contraction									3.20E-03																		
Cell_adhesion_molecules										1.50E-02					4.70E-03							5.70E-03					
Central_carbon_metabolism_in_cancer													4.60E-02														
cGMP_PKG_signaling_pathway					1.30E-02						3.10E-08	5.10E-06	2.60E-03					1.50E-06			5.00E-02	1.30E-02					
Chagas_disease																								5.50E-05			
Chemical_carcinogenesis																			5.00E-02								
Chemokine_signaling_pathway									9.90E-03									5.10E-04						1.70E-03			
Cholinergic_synapse											7.10E-03	3.80E-03						3.80E-03									
Chronic_myeloid_leukemia															4.40E-02												4.80E-02
Circadian_entrainment				3.10E-04					1.00E-02		1.20E-02							2.80E-02									
Collecting_duct_acid_secretion	3.50E-02																										
Complement_and_coagulation_cascades																								1.40E-02			
Cytokine_cytokine_receptor_interaction		3.60E-02																						5.00E-02			
Dilated_cardiomyopathy									5.60E-03		6.50E-06		3.20E-02		4.50E-03					4.40E-02							
Dopaminergic_synapse				9.10E-03								6.60E-04						2.80E-02									
Drug_metabolism_cytochrome_P450		8.30E-04																	1.10E-03								
Drug_metabolism_other_enzymes		2.00E-02																									
ECM_receptor_interaction													2.70E-09		1.90E-04							4.30E-02					
Endocrine_and_other_factor_regulated_calcium_reabsorption										4.80E-02																	
Endocytosis	5.80E-04											6.00E-03				2.10E-02											
Epstein_Barr_virus_infection																								4.50E-02			
ErbB_signaling_pathway			2.80E-03																					9.30E-03			
Estrogen_signaling_pathway												2.50E-02									4.90E-02						
Fat_digestion_and_absorption						3.60E-02																					
Fatty_acid_elongation				1.90E-02																							
Fatty_acid_metabolism				4.70E-03																							
Fc_gamma_R_mediated_phagocytosis	8.00E-05																										
Focal_adhesion									6.50E-03		9.10E-04	2.60E-03	3.60E-11		1.50E-03												
GABAergic_synapse				1.30E-02							7.30E-03		3.30E-02							4.60E-02							
Gap_junction												1.50E-02	3.80E-02								3.20E-02						
Glutamatergic_synapse				1.20E-03														4.50E-03									
Glycerophospholipid_metabolism								4.10E-02						4.10E-02									4.70E-02				
Glycine_serine_and_threonine_metabolism				1.20E-02																							
Glycolysis_Gluconeogenesis	3.40E-02			1.90E-02																							
Glycosaminoglycan_biosynthesis_chondroitin_sulfate_dermatan_sulfate							2.60E-04						7.10E-04														
Glycosphingolipid_biosynthesis_ganglio_series									3.30E-02																		
Hedgehog_signaling_pathway					1.40E-02																						
Hematopoietic_cell_lineage																								8.40E-03			
Hepatitis_B			3.90E-02																								
Hippo_signaling_pathway							1.10E-02						4.00E-02								1.10E-02						
HTLV_I_infection																								2.70E-04			
Huntington_disease																										1.50E-02	
Hypertrophic_cardiomyopathy									3.90E-03		1.60E-04	8.60E-03			3.10E-03										3.80E-02		
Inflammatory_mediator_regulation_of_TRP_channels				2.50E-02		3.60E-02												1.90E-03									
Influenza_A	2.00E-02																							1.00E-02			
Inositol_phosphate_metabolism				2.30E-02							1.40E-02																
Insulin_resistance												3.20E-03					3.00E-02								3.20E-02		
Insulin_secretion				4.60E-02	2.20E-02							4.50E-02															
Insulin_signaling_pathway			1.00E-02																						7.20E-03		
Jak_STAT_signaling_pathway																								1.10E-02			
Legionellosis	3.20E-03																										
Leishmaniasis	1.80E-05																										
Leukocyte_transendothelial_migration	1.40E-02																										
Long_term_depression				1.20E-02								1.20E-02															
Long_term_potentiation				1.80E-02																							
Lysosome	4.20E-06																										
Malaria																								1.90E-02			
MAPK_signaling_pathway											1.40E-02																
Measles	2.70E-02																							2.10E-02			
Melanogenesis																			7.70E-03		1.50E-02						
Melanoma													1.70E-02		4.20E-02												
Metabolic_pathways	2.60E-02			2.20E-03		1.60E-02		3.50E-05																			
Metabolism_of_xenobiotics_by_cytochrome_P450																			9.00E-03								
Morphine_addiction				4.80E-03					3.10E-02		2.40E-03																
mRNA_surveillance_pathway														1.50E-03		1.50E-02											
Natural_killer_cell_mediated_cytotoxicity	1.70E-02																							9.50E-04			
Neuroactive_ligand_receptor_interaction				4.50E-03						2.90E-02								1.40E-03									
NF_kappa_B_signaling_pathway																								4.30E-04			
Nicotine_addiction				1.30E-02																							
Non_alcoholic_fatty_liver_disease																										1.30E-02	
Notch_signaling_pathway																					1.50E-02						
Oocyte_meiosis					4.50E-03																						
Osteoclast_differentiation	1.10E-05																							5.90E-03			
Other_glycan_degradation														1.80E-03													
Other_types_of_O_glycan_biosynthesis							1.30E-02																				
Oxidative_phosphorylation	9.80E-03																									1.70E-03	
Oxytocin_signaling_pathway									1.10E-02		3.40E-04	9.40E-04						7.70E-03			4.40E-03						
Parkinson_disease								7.30E-03																		9.70E-03	
Pathways_in_cancer					1.60E-02		1.10E-02		4.10E-02		3.30E-04		5.50E-07		1.40E-02			1.80E-03			8.40E-04						
Pentose_phosphate_pathway	4.30E-02																										
Peroxisome						4.40E-03																					
Pertussis	1.60E-02																										
Phagosome	1.50E-05																										
Phosphatidylinositol_signaling_system											1.40E-02	2.40E-02															
PI3K_Akt_signaling_pathway							2.40E-02				4.70E-02		7.40E-07		2.60E-02												
Platelet_activation											2.80E-04	7.40E-04	4.00E-04														
Primary_immunodeficiency																								3.30E-02			
Prion_diseases								2.70E-02																			
Progesterone_mediated_oocyte_maturation					2.40E-02																						
Prostate_cancer												1.50E-02															
Protein_digestion_and_absorption													3.00E-08														
Protein_processing_in_endoplasmic_reticulum												4.00E-02															
Proteoglycans_in_cancer							1.60E-02						2.90E-04					2.80E-02									
Pyrimidine_metabolism																											4.80E-02
Pyruvate_metabolism										3.60E-02																	
Rap1_signaling_pathway							2.00E-02				1.00E-02	2.20E-02	3.30E-02					3.90E-04			3.30E-03						
Ras_signaling_pathway									3.10E-02				2.90E-03					7.60E-03						4.60E-02			
Regulation_of_actin_cytoskeleton											1.00E-02																
Regulation_of_lipolysis_in_adipocytes																		1.80E-02									
Renin_angiotensin_system																		7.90E-03									
Renin_secretion											9.30E-03	6.00E-04						1.40E-04			3.90E-02						
Retrograde_endocannabinoid_signaling				2.20E-03								2.80E-02						3.60E-02									
Ribosome																					1.60E-03						
RNA_degradation																3.40E-02											
Salivary_secretion												4.70E-02															
Salmonella_infection	1.70E-03																										
Serotonergic_synapse																		5.00E-02									
Shigellosis	1.60E-03																										
Signaling_pathways_regulating_pluripotency_of_stem_cells							7.50E-03																				
Small_cell_lung_cancer													8.90E-03		2.00E-02		3.70E-02										
Sphingolipid_signaling_pathway																		2.10E-02									
Staphylococcus_aureus_infection	5.70E-04																										
Steroid_biosynthesis								3.60E-05																			
Sulfur_relay_system						1.40E-02																					
Synaptic_vesicle_cycle	6.90E-03																										
T_cell_receptor_signaling_pathway																								1.30E-06			
TGF_beta_signaling_pathway													8.40E-03			4.40E-02											
Thyroid_hormone_signaling_pathway			3.40E-03									4.40E-03									2.70E-02			3.10E-02			
Thyroid_hormone_synthesis																					1.30E-02						
TNF_signaling_pathway															1.40E-02									6.50E-03			
Toll_like_receptor_signaling_pathway																								2.30E-02			
Toxoplasmosis	4.00E-02														2.20E-02												
Tuberculosis	2.90E-04																										
Type_II_diabetes_mellitus																								1.70E-02			
Ubiquitin_mediated_proteolysis																											4.70E-02
Valine_leucine_and_isoleucine_degradation				2.20E-02																							
Vascular_smooth_muscle_contraction											6.90E-07	4.50E-07	4.00E-02					5.60E-03			9.90E-03	3.40E-02					
Vibrio_cholerae_infection	1.40E-02																										
Wnt_signaling_pathway						4.40E-02	2.40E-02														9.50E-05						

P values of each pathway with p value < 0.05 in every single cancer type are shown.

table S12. Pathway enrichment analysis of the top 200 genes showing positive correlated with missense mutation loads.

Pathway	AML	ACC	BLCA	LGG	BRCA	COAD	ESCA	GBM	HNSC	KICH	KIRC	KIRP	LIHC	LUAD	LUSC	DLBC	OV	PAAD	PRAD	SARC	SKCM	STAD	TGCT	THCA	UCS	UCEC	UVM
Alcoholism			1.10E-02																								
Alzheimer_disease											1.60E-02					2.50E-03						8.70E-06		1.00E-05			
Amino_sugar_and_nucleotide_sugar_metabolism																										1.10E-02	
Aminoacyl_tRNA_biosynthesis													7.30E-03														
AMPK_signaling_pathway							3.80E-02	9.70E-03																			
Antigen_processing_and_presentation			2.00E-02																								
Ascorbate_and_aldarate_metabolism												2.00E-02															
Axon_guidance														3.60E-02													
Bacterial_invasion_of_epithelial_cells														4.10E-02													
Base_excision_repair		6.40E-03																									
beta_Alanine_metabolism																											4.80E-02
Biosynthesis_of_antibiotics												2.80E-02				3.00E-03						4.40E-02				1.60E-02	
Carbon_metabolism					4.70E-02						4.80E-02					2.20E-02						2.90E-02					
Cell_cycle		2.70E-14		8.50E-19	6.10E-22					1.80E-21					5.00E-04		3.20E-02		2.30E-06	9.30E-17		4.50E-10					
Central_carbon_metabolism_in_cancer								4.70E-03																			
Chagas_disease						3.00E-02																					
Citrate_cycle																								6.70E-03			
Colorectal_cancer																									4.50E-02		
D_Glutamine_and_D_glutamate_metabolism														3.90E-02													
DNA_replication		7.40E-04		8.70E-08	6.20E-05					6.10E-04										8.40E-03					1.10E-02		
Endocytosis														1.30E-02													
Epstein_Barr_virus_infection													3.50E-04							2.40E-02							
Fanconi_anemia_pathway				2.00E-05	3.60E-03															3.60E-04							
Fat_digestion_and_absorption							7.60E-03																				
FoxO_signaling_pathway						1.70E-02		1.40E-02																			
Fructose_and_mannose_metabolism							4.30E-02																				
GABAergic_synapse									9.60E-03																		
Glycolysis_Gluconeogenesis					4.60E-02							1.70E-02															
Glyoxylate_and_dicarboxylate_metabolism																								4.80E-02			
Hepatitis_B										2.10E-02																	
Hepatitis_C																									7.20E-03		
Herpes_simplex_infection																									2.40E-03		
HIF_1_signaling_pathway								3.90E-03																			
Homologous_recombination				2.50E-04	3.70E-04								4.50E-02							4.60E-03							
HTLV_I_infection		2.70E-03								9.80E-05																	
Huntington_disease											8.50E-03					7.80E-05		6.60E-03				6.50E-06		6.20E-06			
Long_term_potentiation														2.70E-02													
Lysosome																									8.90E-04		
MAPK_signaling_pathway						2.40E-02																					
Metabolic_pathways							1.80E-05				2.50E-06	6.50E-03	4.20E-02			5.40E-03						4.70E-03		1.60E-06			
MicroRNAs_in_cancer					6.90E-03					3.80E-03																	
Mineral_absorption						1.70E-03																					
Mismatch_repair			3.70E-03	1.90E-03	3.10E-02																						
Morphine_addiction									1.20E-02																		
mRNA_surveillance_pathway																			1.70E-03								
Nicotine_addiction									6.90E-03																		
Non_alcoholic_fatty_liver_disease											9.90E-03					1.30E-03						2.80E-02		2.40E-05			
Nucleotide_excision_repair											1.90E-02																
One_carbon_pool_by_folate																						3.70E-02					
Oocyte_meiosis		4.00E-06		2.30E-06	4.90E-09					2.20E-08					1.50E-03				3.70E-03	4.40E-06		2.60E-02					
Oxidative_phosphorylation											5.40E-03					1.00E-04		4.30E-03				7.40E-07		1.20E-07			
p53_signaling_pathway		1.00E-03		6.90E-06	1.20E-03	4.00E-02				8.50E-05										7.70E-03		5.30E-04					
Parkinson_disease											7.40E-03					8.60E-04		2.40E-02				1.50E-06		1.40E-05			
Pentose_and_glucuronate_interconversions												1.90E-04															
Pentose_phosphate_pathway									3.30E-02		4.80E-02																
Peroxisome												2.90E-02														4.60E-02	
Progesterone_mediated_oocyte_maturation		5.90E-05		3.90E-06	8.00E-07					3.70E-07					3.10E-03				9.30E-03	6.90E-06		2.10E-03					
Prolactin_signaling_pathway						4.60E-02																					
Proteasome													6.30E-07							1.50E-02		3.90E-06					
Protein_processing_in_endoplasmic_reticulum			8.40E-03																								
Purine_metabolism																					3.50E-02						
Pyrimidine_metabolism									1.90E-02						3.10E-02												
Regulation_of_actin_cytoskeleton														4.50E-03													
Renal_cell_carcinoma								5.00E-03																			
Retrograde_endocannabinoid_signaling									1.70E-02																		
Ribosome										3.70E-03			5.00E-03	1.10E-02		5.40E-10								6.20E-05			
Ribosome_biogenesis_in_eukaryotes	4.20E-04			2.40E-03					1.00E-02				3.40E-03														
RNA_transport	3.70E-02			9.10E-05									3.90E-03	4.20E-05													4.60E-02
Signaling_pathways_regulating_pluripotency_of_stem_cells						2.10E-02																					
Spliceosome			9.70E-03																								
Steroid_biosynthesis			3.00E-02															1.50E-03									
Systemic_lupus_erythematosus			2.40E-03																								
T_cell_receptor_signaling_pathway														1.80E-02													
Thyroid_hormone_signaling_pathway								3.30E-02																			
Tight_junction														4.50E-02													
TNF_signaling_pathway																									4.70E-02		
Ubiquitin_mediated_proteolysis																				2.20E-02							
Valine_leucine_and_isoleucine_degradation											1.90E-02																
VEGF_signaling_pathway														2.20E-02													
Viral_carcinogenesis																									4.80E-02		

P values of each pathway with p value < 0.05 in every single cancer type are shown.

table S13. Pathway enrichment analysis of the top 200 genes showing negative correlated with missense mutation loads.

Pathway	AML	ACC	BLCA	LGG	BRCA	COAD	ESCA	GBM	HNSC	KICH	KIRC	KIRP	LIHC	LUAD	LUSC	DLBC	OV	PAAD	PRAD	SARC	SKCM	STAD	TGCT	THCA	UCS	UCEC	UVM
Adrenergic_signaling_in_cardiomyocytes					4.10E-04													3.60E-02									
Aldosterone_synthesis_and_secretion					4.00E-02							1.30E-02						3.20E-02				2.50E-02					
Alzheimer_disease												4.10E-02															
Amoebiasis													1.70E-05														
Arrhythmogenic_right_ventricular_cardiomyopathy															2.90E-02												
Axon_guidance															3.10E-02												
Bacterial_invasion_of_epithelial_cells	2.40E-02								4.30E-02																		
beta_Alanine_metabolism								3.60E-02																			
Bile_secretion																					2.40E-02						
Biosynthesis_of_antibiotics	3.00E-02			5.00E-02				1.90E-04																			
Butanoate_metabolism										2.10E-02																	
Calcium_signaling_pathway											2.30E-06	3.90E-03															
cAMP_signaling_pathway				3.30E-03	2.40E-03													2.60E-02									
Carbon_metabolism	2.20E-02			1.40E-02																							
Cell_adhesion_molecules																						2.40E-02					
Central_carbon_metabolism_in_cancer											3.00E-02																
cGMP_PKG_signaling_pathway					2.00E-02						1.10E-06	5.30E-04	9.00E-03					2.80E-03				4.00E-02					
Chemical_carcinogenesis								4.20E-02											3.20E-02								
Chemokine_signaling_pathway			1.60E-02															2.10E-02									
Cholinergic_synapse											2.90E-02	3.60E-02															
Chronic_myeloid_leukemia																											4.00E-02
Circadian_entrainment				3.10E-02								2.20E-02															
Complement_and_coagulation_cascades													3.90E-02											3.50E-02			
Cytokine_cytokine_receptor_interaction								2.30E-02																			
Cytosolic_DNA_sensing_pathway																							3.80E-02				
D_Glutamine_and_D_glutamate_metabolism				4.90E-02																							
Dilated_cardiomyopathy															1.10E-03												
Dopaminergic_synapse											4.60E-02	8.80E-05															
Drug_metabolism_cytochrome_P450																			2.10E-02								
Drug_metabolism_other_enzymes	2.70E-02																										
ECM_receptor_interaction													3.30E-07		8.90E-03												
Endocytosis	1.10E-02																										
Epithelial_cell_signaling_in_Helicobacter_pylori_infection			3.70E-02																								
ErbB_signaling_pathway																		3.80E-02									
Estrogen_signaling_pathway												2.50E-02															
Fat_digestion_and_absorption																		4.30E-02									
Fc_gamma_R_mediated_phagocytosis	3.10E-02																	3.50E-02									
Focal_adhesion											2.10E-02		3.20E-08		4.40E-02												
FoxO_signaling_pathway																							1.90E-02				
GABAergic_synapse											1.20E-02								3.70E-02								
Gastric_acid_secretion								3.30E-02																			
Glucagon_signaling_pathway				3.60E-02																							
Glutamatergic_synapse					2.20E-02							3.90E-02															
Glycolysis_Gluconeogenesis								2.70E-02																			
Glycosaminoglycan_biosynthesis_chondroitin_sulfate_dermatan_sulfate													1.90E-02														
Glycosaminoglycan_degradation	2.90E-02																										
Glycosphingolipid_biosynthesis_lacto_and_neolacto_series									2.70E-02																		
Hepatitis_B			2.10E-02																								
Herpes_simplex_infection																											4.40E-02
Hippo_signaling_pathway																	2.60E-02										
HTLV_I_infection																								5.00E-02			
Hypertrophic_cardiomyopathy															7.80E-04												
Inflammatory_mediator_regulation_of_TRP_channels				3.50E-02								2.40E-02						1.40E-03									
Influenza_A	3.70E-02		4.20E-02																					9.30E-03			
Insulin_resistance																	3.20E-02										
Insulin_secretion					4.50E-02																						
Insulin_signaling_pathway																									2.30E-03		
Legionellosis	7.00E-03																										
Leishmaniasis	3.20E-03																										
Leukocyte_transendothelial_migration	6.40E-03																						4.70E-02				
Long_term_depression												4.50E-03															
Long_term_potentiation												6.40E-03															
Lysosome	3.00E-04																										
Malaria																								1.40E-02			
MAPK_signaling_pathway					3.20E-02						1.80E-02																
Metabolic_pathways				2.80E-02				3.10E-04																			
Metabolism_of_xenobiotics_by_cytochrome_P450								3.50E-02											2.60E-02								
Morphine_addiction				2.70E-02							2.60E-03																
mRNA_surveillance_pathway														2.60E-03		2.80E-02											
Mucin_type_O_Glycan_biosynthesis									3.80E-02																		
Neuroactive_ligand_receptor_interaction				2.30E-02																							
Neurotrophin_signaling_pathway																									4.80E-02		
NF_kappa_B_signaling_pathway																								1.30E-02			
Osteoclast_differentiation	5.10E-04																							4.70E-02			
Other_types_of_O_glycan_biosynthesis							2.70E-02																				
Oxidative_phosphorylation								4.00E-02																			
Oxytocin_signaling_pathway			3.00E-02								6.30E-03	8.60E-03															
p53_signaling_pathway															2.50E-02												
Parkinson_disease								1.20E-02																			
Pathways_in_cancer					1.10E-02						2.60E-03		9.50E-03		3.00E-02												
Peroxisome						4.30E-02																					
Pertussis	2.10E-02																							4.30E-02			
Phagosome	1.40E-03																										
Phosphatidylinositol_signaling_system												2.40E-02															
PI3K_Akt_signaling_pathway													7.30E-05														
Platelet_activation	2.40E-03											3.40E-03															
Protein_digestion_and_absorption													4.20E-06														
Proximal_tubule_bicarbonate_reclamation																					1.80E-02						
Purine_metabolism												4.80E-02								4.50E-02							
Regulation_of_autophagy																			4.50E-02								
Renin_secretion					2.20E-02							7.30E-04						1.70E-02									
Renin_angiotensin_system																		9.30E-04									
Retrograde_endocannabinoid_signaling				3.80E-02							2.20E-02	5.50E-03															
Salmonella_infection	3.00E-02																										
Serotonergic_synapse												3.60E-02															
Vascular_smooth_muscle_contraction																						1.40E-02					

P values of each pathway with p value < 0.05 in every single cancer type are shown.

table S14. Pathway enrichment analysis of the top 400 genes showing positive correlated with sense mutation loads.

Pathway	AML	ACC	BLCA	LGG	BRCA	COAD	ESCA	GBM	HNSC	KICH	KIRC	KIRP	LIHC	LUAD	LUSC	DLBC	OV	PAAD	PRAD	SARC	SKCM	STAD	TGCT	THCA	UCS	UCEC	UVM
2_Oxocarboxylic_acid_metabolism												4.00E-02												7.50E-03			
ABC_transporters												5.00E-02															
Adrenergic_signaling_in_cardiomyocytes																							2.90E-02				
Alanine_aspartate_and_glutamate_metabolism												4.70E-04															
Alcoholism			4.30E-03														3.30E-04										
Alzheimer_disease					6.80E-03						1.80E-10		7.20E-04	9.60E-04	2.30E-02	7.20E-10		7.30E-03				9.20E-07		2.10E-04			
Amino_sugar_and_nucleotide_sugar_metabolism							1.50E-02											2.80E-02		5.70E-03						1.60E-02	
Aminoacyl_tRNA_biosynthesis																			9.10E-03								
Amoebiasis								3.70E-02		1.10E-02																	
Antigen_processing_and_presentation			1.10E-02																								
Arachidonic_acid_metabolism									4.20E-02																		
Arrhythmogenic_right_ventricular_cardiomyopathy_(ARVC)																					3.30E-02						
Bacterial_invasion_of_epithelial_cells														2.60E-02					1.80E-02								
Basal_transcription_factors																										1.30E-02	
Base_excision_repair		3.00E-02	1.20E-03																			2.10E-03					
Bile_secretion							5.00E-02					4.10E-02															
Biosynthesis_of_amino_acids					3.50E-04	5.40E-03						2.70E-03										1.00E-03		3.40E-02			
Biosynthesis_of_antibiotics					1.20E-02	4.20E-02			3.70E-02		9.70E-04	1.80E-04				1.30E-02						2.00E-08		1.90E-04		1.10E-02	
Butanoate_metabolism																						3.90E-02					
Calcium_signaling_pathway																							2.10E-03				
cAMP_signaling_pathway																							1.30E-02				
Carbon_metabolism					5.50E-03	1.20E-02					2.50E-02	5.50E-03				3.50E-02						2.80E-06		1.60E-03			
Cardiac_muscle_contraction											1.20E-02				4.40E-03	9.50E-04											
Cell_cycle	4.60E-02	4.70E-10	1.20E-02	6.80E-18	4.70E-18					1.70E-08					2.20E-07			3.00E-02	1.40E-08	1.10E-12		2.90E-13					
Central_carbon_metabolism_in_cancer								2.50E-02																			
cGMP_PKG_signaling_pathway																							1.60E-02				
Cholinergic_synapse																							1.90E-03				
Chronic_myeloid_leukemia								3.60E-02																			
Citrate_cycle											6.60E-03													7.00E-04			
Cocaine_addiction																							7.40E-03				
Cyanoamino_acid_metabolism												7.00E-03															
Cysteine_and_methionine_metabolism											2.60E-03																
Cytokine_cytokine_receptor_interaction																	2.30E-02										
DNA_replication			2.30E-05	6.20E-05	1.50E-06					1.40E-04								1.10E-02		1.70E-06		1.80E-02					
Dopaminergic_synapse																							4.20E-03				
Drug_metabolism_cytochrome_P450							4.70E-02																				
ECM_receptor_interaction				7.10E-03						7.90E-04																	
Endocytosis														1.30E-03							4.40E-03			9.90E-03			
Epstein_Barr_virus_infection													6.20E-03					4.00E-02		4.00E-02							
Fanconi_anemia_pathway		2.40E-02		2.80E-10	8.30E-03										4.60E-03			1.60E-03		2.60E-04	1.20E-02	1.60E-02					
Fat_digestion_and_absorption							1.00E-03																				
Focal_adhesion								2.70E-02																			
Folate_biosynthesis							3.10E-02																				
FoxO_signaling_pathway								8.50E-03																			
Fructose_and_mannose_metabolism							2.60E-02		3.00E-02									4.10E-02									
GABAergic_synapse																							2.20E-03				
Galactose_metabolism							2.90E-03																				
Glutamatergic_synapse																							4.30E-04				
Glycerophospholipid_metabolism																			3.80E-02								
Glycine_serine_and_threonine_metabolism												7.80E-04															
Glycosaminoglycan_biosynthesis_heparan_sulfate_heparin																							6.20E-03				
Glycosaminoglycan_degradation											1.20E-02																
Glyoxylate_and_dicarboxylate_metabolism											3.00E-02	1.40E-02												2.70E-02			
Herpes_simplex_infection																			2.70E-02								
HIF_1_signaling_pathway								2.80E-04																			
Hippo_signaling_pathway														4.40E-02													
Homologous_recombination				1.70E-05	4.70E-03										2.80E-03			4.90E-03		5.50E-05		4.70E-02					
HTLV_I_infection		1.70E-02			1.90E-02																	5.50E-03					
Huntington_disease					6.40E-04						1.30E-08		2.30E-03		6.30E-03	1.20E-12		5.70E-05				7.50E-08		2.20E-04			
Hypertrophic_cardiomyopathy																					4.40E-02						
Influenza_A						3.60E-02																					
Insulin_secretion																							4.60E-02				
Jak_STAT_signaling_pathway																	7.30E-04										
Leishmaniasis						8.50E-04																					
Lysine_degradation	8.90E-03																										
Lysosome											2.40E-04													2.60E-03			
MAPK_signaling_pathway								3.30E-02																			
Maturity_onset_diabetes_of_the_young							1.40E-04																				
Metabolic_pathways					4.90E-02		3.30E-06		4.90E-03		2.30E-14	2.00E-08	2.80E-04			2.70E-04						4.20E-06		1.50E-06	1.90E-02		
Metabolism_of_xenobiotics_by_cytochrome_P450							1.60E-02																				
Mineral_absorption						1.80E-02																			5.00E-03		
Mismatch_repair			2.10E-04	9.50E-04	1.70E-02						2.00E-02									1.70E-02		2.60E-02					
Morphine_addiction																							1.50E-02				
mRNA_surveillance_pathway														1.30E-02					3.20E-02								
Mucin_type_O_Glycan_biosynthesis							2.40E-02																				
Natural_killer_cell_mediated_cytotoxicity						5.10E-03																					
Neuroactive_ligand_receptor_interaction																							2.90E-05				
Neurotrophin_signaling_pathway														4.50E-02													
N_Glycan_biosynthesis			3.20E-02																								
Nicotine_addiction									9.90E-03								1.50E-02						3.50E-05				
Non_alcoholic_fatty_liver_disease_(NAFLD)					2.80E-02						6.70E-09		1.10E-02		4.00E-03	8.80E-11		1.10E-02				4.40E-06		2.90E-04			
Notch_signaling_pathway								5.00E-02																			
Nucleotide_excision_repair			5.80E-03								6.50E-03																
One_carbon_pool_by_folate																						1.70E-02					
Oocyte_meiosis		7.80E-05		8.50E-06	1.60E-06					3.40E-03				2.30E-03	7.80E-05				1.20E-03	2.80E-04		2.20E-04					
Osteoclast_differentiation_						5.50E-04																					
Other_glycan_degradation											1.00E-02																
Oxidative_phosphorylation					4.40E-03						1.00E-10		1.70E-05		8.80E-05	6.50E-12		8.10E-05				8.00E-07		1.10E-07			
p53_signaling_pathway		2.50E-03		4.50E-05	1.80E-04														4.10E-02	2.20E-02		1.50E-05					
Parkinson_disease					6.70E-03						4.60E-11		2.20E-03		2.60E-03	1.80E-10		2.20E-03				2.00E-09		1.60E-04			
Pathogenic_Escherichia_coli_infection																		3.40E-02									
Pathways_in_cancer						4.80E-02		1.80E-02																			
Pentose_and_glucuronate_interconversions												4.50E-03															
Peroxisome												2.40E-05	5.00E-02													2.60E-02	
Phagosome																								3.30E-02			
Phosphatidylinositol_signaling_system																										1.40E-02	
PI3K_Akt_signaling_pathway								1.60E-02									3.90E-02										
Progesterone_mediated_oocyte_maturation		7.00E-05		2.90E-04	3.80E-05					3.60E-03					7.00E-05				1.50E-03	4.20E-05		7.00E-04					
Proteasome					2.00E-02								1.60E-11			3.10E-02		3.90E-03		3.90E-03		1.30E-03					
Protein_processing_in_endoplasmic_reticulum			9.00E-03											1.00E-03													
Purine_metabolism										4.80E-02									7.40E-03								
Pyrimidine_metabolism			1.40E-02		1.20E-02																	8.60E-03					
Pyruvate_metabolism											1.80E-02																
Rap1_signaling_pathway								3.40E-03																			
Regulation_of_actin_cytoskeleton														5.70E-03													
Renal_cell_carcinoma								1.20E-04																			
Retrograde_endocannabinoid_signaling																	3.50E-02						1.90E-04				
Ribosome					4.40E-02					1.40E-18			8.20E-07	2.70E-02	2.20E-02	6.80E-29								8.80E-07			
Ribosome_biogenesis_in_eukaryotes	4.80E-02								3.70E-02	4.80E-02								1.80E-02									
RNA_degradation																			3.80E-03								
RNA_polymerase													4.10E-02														
RNA_transport	1.90E-02			6.70E-03									2.30E-02	7.90E-05					6.40E-03			2.30E-02					
Serotonergic_synapse									3.10E-02																		
Shigellosis														4.90E-02				1.90E-02									
Signaling_pathways_regulating_pluripotency_of_stem_cells								3.50E-02																			
Small_cell_lung_cancer				6.40E-03																							
Spliceosome			2.20E-05		1.20E-03								4.20E-02		6.20E-03			4.80E-03									
Staphylococcus_aureus_infection						3.00E-02																					
Steroid_biosynthesis																		5.30E-06									
Systemic_lupus_erythematosus			1.70E-03														1.90E-05										
Terpenoid_backbone_biosynthesis																						2.30E-02					
Thyroid_hormone_signaling_pathway								4.80E-02																			
Tight_junction							2.00E-02							2.80E-02													
Toll_like_receptor_signaling_pathway						2.80E-02																					
Transcriptional_misregulation_in_cancer	4.90E-02																7.30E-03										
Tuberculosis						4.00E-02																					
Tyrosine_metabolism												2.80E-02															
Ubiquitin_mediated_proteolysis	2.10E-02													2.70E-03					5.20E-03								
Valine_leucine_and_isoleucine_degradation											6.50E-03	1.80E-03														1.50E-02	
Vasopressin_regulated_water_reabsorption																							3.20E-02	2.20E-02			
Vibrio_cholerae_infection																								9.00E-03			

P values of each pathway with p value < 0.05 in every single cancer type are shown.

table S15. Pathway enrichment analysis of the top 400 genes showing negative correlated with sense mutation loads.

Pathway	AML	ACC	BLCA	LGG	BRCA	COAD	ESCA	GBM	HNSC	KICH	KIRC	KIRP	LIHC	LUAD	LUSC	DLBC	OV	PAAD	PRAD	SARC	SKCM	STAD	TGCT	THCA	UCS	UCEC	UVM
ABC_transporters			3.50E-03																					2.90E-02			
Adherens_junction											1.60E-02											6.60E-03					
Adipocytokine_signaling_pathway																		2.10E-02	1.00E-02								
Adrenergic_signaling_in_cardiomyocytes				1.40E-02					3.30E-06				3.50E-02					1.50E-03			2.60E-02	1.10E-02					
African_trypanosomiasis																							1.00E-02				
Alanine_aspartate_and_glutamate_metabolism				4.00E-02																							
Alcoholism																									2.70E-02		
Aldosterone_synthesis_and_secretion												2.80E-02						7.30E-03				4.70E-02					
Allograft_rejection																							3.50E-04				
Alzheimer_disease								2.00E-02																		2.10E-02	
Amoebiasis											2.30E-02		1.30E-05		7.70E-04												
AMPK_signaling_pathway				4.90E-03																							
Antigen_processing_and_presentation																							4.80E-02				
Apoptosis																							2.30E-02				
Arrhythmogenic_right_ventricular_cardiomyopathy									6.60E-06		1.60E-02	3.70E-03															
Autoimmune_thyroid_disease																							1.10E-02				
Axon_guidance									3.30E-02		1.30E-03				9.40E-03									4.50E-02			
Basal_cell_carcinoma							5.30E-05														4.50E-03						
Bile_secretion																					4.80E-02						
Biosynthesis_of_amino_acids				1.80E-04																							
Biosynthesis_of_antibiotics				1.90E-04				3.20E-04																			
Biosynthesis_of_unsaturated_fatty_acids				1.40E-03																							
Calcium_signaling_pathway									2.00E-02		8.30E-05	3.80E-04															
cAMP_signaling_pathway				3.70E-03								3.00E-03	2.40E-02								4.30E-02						
Carbon_metabolism				3.00E-05				7.50E-03																			
Cardiac_muscle_contraction									1.20E-06																		
Cell_adhesion_molecules																						9.70E-03	7.70E-07				
Cell_cycle																3.50E-03											
Central_carbon_metabolism_in_cancer													4.60E-02														
cGMP_PKG_signaling_pathway									1.20E-03		1.60E-04	1.90E-04	1.90E-04					6.30E-06			4.60E-02	1.80E-03					
Chagas_disease																							6.50E-08				
Chemokine_signaling_pathway									2.50E-02														2.30E-09				
Choline_metabolism_in_cancer																		4.00E-03				2.70E-02					
Cholinergic_synapse																		6.40E-03									
Circadian_entrainment				1.70E-02					9.10E-03			5.00E-02															
Circadian_rhythm					1.60E-02																						
Citrate_cycle								2.20E-02																			
Cocaine_addiction																								3.90E-02			
Colorectal_cancer																						2.00E-02					
Cytokine_cytokine_receptor_interaction																							9.10E-13				
Dilated_cardiomyopathy									4.30E-07		1.80E-03	8.40E-03	3.20E-02														
Dopaminergic_synapse																		1.20E-02									
Dorso_ventral_axis_formation													1.90E-02														
Drug_metabolism_cytochrome_P450		5.20E-04																	1.70E-03								
ECM_receptor_interaction									2.40E-02		2.10E-03		1.80E-06		4.30E-06												
Endocytosis								3.50E-02				4.60E-02				7.80E-03											
ErbB_signaling_pathway			4.10E-03													4.90E-02											
Estrogen_signaling_pathway																					4.60E-02						
Fat_digestion_and_absorption						4.20E-02																					
Fatty_acid_metabolism				5.50E-04																							
Fc_epsilon_RI_signaling_pathway			2.10E-02																				8.50E-03				
Fc_gamma_R_mediated_phagocytosis																							2.20E-02				
Focal_adhesion									1.30E-04		2.20E-07		5.20E-08		1.20E-06												
FoxO_signaling_pathway																5.40E-03											
GABAergic_synapse				3.70E-02					2.20E-02				3.30E-02														
Gap_junction												3.80E-02						4.30E-02			3.00E-02						
Glutamatergic_synapse				3.70E-02																							
Glutathione_metabolism																			1.60E-02								
Glycerophospholipid_metabolism								1.20E-02						4.30E-02													
Glycine_serine_and_threonine_metabolism				9.70E-03																							
Glycosaminoglycan_biosynthesis_chondroitin_sulfate_dermatan_sulfate							5.30E-03						4.70E-05														
GnRH_signaling_pathway																		4.80E-02									
Graft_versus_host_disease																							1.50E-03				
Hematopoietic_cell_lineage																							1.80E-06				
Herpes_simplex_infection																							8.40E-03				
Hippo_signaling_pathway							1.60E-03										1.00E-02				3.00E-02						
HTLV_I_infection																							7.10E-03				
Huntington_disease								1.20E-04																		4.30E-03	
Hypertrophic_cardiomyopathy									2.00E-07		5.50E-03	2.40E-02															
Inflammatory_bowel_disease																							1.30E-03	2.90E-03			
Inflammatory_mediator_regulation_of_TRP_channels						4.60E-02																					
Influenza_A	1.20E-02																						6.10E-04				
Insulin_resistance																						3.50E-02					
Insulin_secretion												3.30E-02															
Insulin_signaling_pathway			1.60E-02																			8.30E-03					
Intestinal_immune_network_for_IgA_production																							7.30E-03				
Jak_STAT_signaling_pathway																				2.30E-02			1.40E-03				
Leishmaniasis																							3.80E-02				
Leukocyte_transendothelial_migration									2.40E-02		1.10E-02												3.50E-02				
Malaria																							1.60E-03				
MAPK_signaling_pathway											6.80E-03											3.60E-03					
Measles	4.70E-02																						4.10E-05				
Melanogenesis							3.40E-02														1.40E-02						
Melanoma													1.70E-02		1.00E-02												
Metabolic_pathways	2.50E-02			3.00E-04		8.80E-03		1.30E-06									2.50E-02										
Metabolism_of_xenobiotics_by_cytochrome_P450																	1.60E-02										
Morphine_addiction				4.70E-02					7.40E-03		1.20E-02	6.30E-04	1.20E-02														
mRNA_surveillance_pathway														1.60E-03		1.50E-02											
mTOR_signaling_pathway			4.60E-02																								
Natural_killer_cell_mediated_cytotoxicity																							1.40E-08				
Neuroactive_ligand_receptor_interaction				7.00E-03								3.20E-02						2.30E-02									
NF_kappa_B_signaling_pathway																							8.30E-05				
Nicotine_addiction				1.10E-02																							
NOD_like_receptor_signaling_pathway																							8.10E-05				
Non_alcoholic_fatty_liver_disease								3.20E-02																		3.40E-03	
Notch_signaling_pathway												1.80E-02									1.50E-02						
Oocyte_meiosis					1.20E-02																						
Osteoclast_differentiation																							7.30E-06				
Other_glycan_degradation														2.50E-02													
Other_types_of_O_glycan_biosynthesis							1.80E-02																				
Oxidative_phosphorylation								3.20E-04																		6.20E-03	
Oxytocin_signaling_pathway									3.00E-03		1.70E-02										4.00E-03	1.70E-02					
p53_signaling_pathway															3.60E-02												
Parkinson_disease								4.80E-06																		8.80E-03	
Pathways_in_cancer			4.20E-02		3.40E-03		1.80E-03		3.50E-02		1.70E-04	3.80E-02	5.70E-04		1.10E-04	2.20E-02		1.50E-02			1.10E-02	3.20E-02					
Peroxisome						1.30E-03																					
Pertussis																							1.30E-04				
PI3K_Akt_signaling_pathway					3.90E-02		4.70E-02				2.70E-02		3.40E-05		2.10E-04												
Platelet_activation											5.60E-03	2.00E-02	4.00E-04		3.40E-02			4.60E-02									
PPAR_signaling_pathway				1.50E-02																							
Primary_immunodeficiency																							2.20E-04				
Prion_diseases																							1.00E-02				
Protein_digestion_and_absorption		3.70E-02										3.80E-02	2.00E-06		2.40E-02												
Proteoglycans_in_cancer							2.80E-02				2.40E-02		3.30E-03		3.30E-02			3.10E-02									
Proximal_tubule_bicarbonate_reclamation																				4.70E-02							
Purine_metabolism												1.20E-02	3.30E-02														
Pyruvate_metabolism								8.20E-03																			
Rap1_signaling_pathway											2.80E-05	1.30E-02						3.80E-02			2.90E-03		4.90E-02				
Ras_signaling_pathway			3.90E-02								7.40E-03		2.10E-02														
Regulation_of_actin_cytoskeleton									4.70E-02		1.40E-03		3.40E-02		4.30E-02												
Regulation_of_lipolysis_in_adipocytes																		9.80E-03									
Renin_angiotensin_system																		4.70E-03									
Renin_secretion												1.10E-02						1.50E-02		1.60E-02	3.80E-02						
Retrograde_endocannabinoid_signaling				2.20E-02																							
Rheumatoid_arthritis																							2.60E-06				
Ribosome																	6.60E-08				3.40E-04						
Salivary_secretion									2.30E-02									4.00E-02									
Salmonella_infection																							2.90E-04				
Serotonergic_synapse																		2.60E-02									
Shigellosis																							1.30E-03				
Signaling_pathways_regulating_pluripotency_of_stem_cells							4.10E-02																				
Small_cell_lung_cancer											3.20E-02		8.90E-03		9.90E-04												
Sphingolipid_metabolism																		5.30E-03									
Staphylococcus_aureus_infection																							7.00E-05				
Steroid_biosynthesis								5.90E-04																			
Sulfur_relay_system						1.60E-02																				1.10E-02	
Systemic_lupus_erythematosus																							7.40E-04				
T_cell_receptor_signaling_pathway																							7.80E-09				
TGF_beta_signaling_pathway													3.20E-02			2.10E-03											
Thyroid_hormone_signaling_pathway			1.40E-03									1.00E-02	1.00E-02								2.50E-02						
Thyroid_hormone_synthesis																					1.20E-02						
Tight_junction									4.50E-02																		
TNF_signaling_pathway															1.40E-02								9.40E-05				
Toll_like_receptor_signaling_pathway																							1.90E-05				
Toxoplasmosis															2.30E-02								3.70E-03				
Transcriptional_misregulation_in_cancer					2.80E-02															4.20E-02					2.10E-02		
Tuberculosis																							7.20E-04				
Type_I_diabetes_mellitus																							7.10E-04				
Vascular_smooth_muscle_contraction									2.50E-02		8.00E-04	1.90E-04									9.20E-03	1.50E-02					
VEGF_signaling_pathway						3.30E-02																					
Viral_myocarditis																							1.40E-05				
Wnt_signaling_pathway				3.00E-02		2.00E-02	1.20E-02														8.40E-05				2.60E-02		

P values of each pathway with p value < 0.05 in every single cancer type are shown.

table S16. Pathway enrichment analysis of the top 200 genes showing positive correlated with sense mutation loads.

Pathway	AML	ACC	BLCA	LGG	BRCA	COAD	ESCA	GBM	HNSC	KICH	KIRC	KIRP	LIHC	LUAD	LUSC	DLBC	OV	PAAD	PRAD	SARC	SKCM	STAD	TGCT	THCA	UCS	UCEC	UVM
Alcoholism			1.10E-02																								
Alzheimer's_disease											4.00E-05					2.00E-04						4.20E-06		4.90E-05			
Aminoacyl_tRNA_biosynthesis													8.60E-03														
Axon_guidance														3.90E-02													
Bacterial_invasion_of_epithelial_cells														4.40E-02													
Base_excision_repair		4.70E-03																									
Biosynthesis_of_antibiotics																1.40E-02						2.10E-04		5.40E-03			
Calcium_signaling_pathway																							1.30E-02				
Carbon_metabolism																						5.50E-03					
Cardiac_muscle_contraction																5.30E-03											
Cell_cycle		3.10E-10	2.90E-02	3.40E-11	1.50E-12					7.70E-09					1.10E-06			2.40E-02	1.10E-04	1.20E-08		5.30E-15					
Chronic_myeloid_leukemia																	4.70E-02										
Citrate_cycle																								6.30E-03			
Collecting_duct_acid_secretion																								4.60E-02			
Cytokine_cytokine_receptor_interaction																	4.20E-03										
Cytosolic_DNA_sensing_pathway																									3.40E-02		
D_Glutamine_and_D_glutamate_metabolism														4.00E-02													
DNA_replication				3.80E-03	7.40E-05													1.30E-03									
ECM_receptor_interaction				4.10E-02						1.50E-02																	
Endocytosis														1.50E-02													
Epstein_Barr_virus_infection													2.20E-03					3.70E-03		2.10E-02							
Fanconi_anemia_pathway				1.10E-02														6.50E-04									
Fat_digestion_and_absorption							6.00E-03																				
Fructose_and_mannose_metabolism							3.70E-02																				
GABAergic_synapse									6.50E-03																		
Hepatitis_B																	5.70E-03										
HIF_1_signaling_pathway								4.50E-02									2.40E-02										
Homologous_recombination				2.00E-03														5.40E-04									
HTLV_I_infection		1.70E-02															2.50E-02					3.00E-02					
Huntington's_disease											1.20E-04					2.80E-05		4.20E-02				3.00E-06		2.80E-05			
Inflammatory_bowel_disease																	3.50E-02										
Inositol_phosphate_metabolism																										3.60E-02	
Insulin_resistance																										4.90E-03	
Jak_STAT_signaling_pathway																	1.20E-03										
Long_term_potentiation														2.90E-02													
Lysosome											1.70E-02																
Metabolic_pathways							3.90E-03		3.40E-02		3.80E-05	6.40E-04				6.80E-03						1.30E-04		1.60E-04			
Mineral_absorption						1.20E-02																			1.40E-02		
Mismatch_repair			3.80E-03	1.70E-02	3.30E-02																						
Morphine_addiction									8.30E-03																		
Neuroactive_ligand_receptor_interaction																							4.60E-05				
Nicotine_addiction									5.10E-03														2.50E-04				
Non_alcoholic_fatty_liver_disease											9.80E-05					8.30E-05						2.90E-04		1.90E-05			
One_carbon_pool_by_folate																						2.70E-03					
Oocyte_meiosis		9.00E-04		2.60E-03	7.20E-06					3.20E-02				2.40E-02	2.80E-05					8.00E-03		9.10E-04					
Oxidative_phosphorylation											5.10E-06					4.30E-06		3.20E-02				3.50E-07		9.30E-08			
p53_signaling_pathway		6.20E-04			1.40E-03					3.70E-02												2.60E-03					
Parkinson's_disease											9.10E-06					4.90E-05						9.20E-08		1.10E-05			
Pathways_in_cancer																	1.20E-02										
Pentose_phosphate_pathway									2.70E-02																		
Peroxisome												7.90E-03															
Phagosome																								1.30E-02			
Phosphatidylinositol_signaling_system																										1.80E-02	
PI3K_Akt_signaling_pathway																	1.50E-02										
Progesterone_mediated_oocyte_maturation		2.70E-04			9.40E-05										5.00E-05				4.30E-02	3.10E-03		3.10E-05					
Proteasome													8.80E-07					2.70E-03		1.30E-04		2.50E-02					
Pyrimidine_metabolism															3.00E-02												
Regulation_of_actin_cytoskeleton														5.30E-03													
Retrograde_endocannabinoid_signaling									1.20E-02														7.80E-03				
Ribosome										2.20E-08			6.40E-03	1.20E-02		3.80E-16								1.70E-03			
Ribosome_biogenesis_in_eukaryotes				4.10E-02									2.20E-02														
RNA_degradation																			4.90E-03								
RNA_transport	1.20E-02										5.40E-03		1.90E-02	7.70E-06													
Small_cell_lung_cancer				3.90E-02																							
Spliceosome			2.40E-03		1.30E-03																						
Starch_and_sucrose_metabolism																										4.50E-02	
Steroid_biosynthesis			3.10E-02															2.90E-02									
Systemic_lupus_erythematosus			2.50E-03														1.70E-02										
T_cell_receptor_signaling_pathway														2.00E-02													
Tight_junction							4.10E-02																				
Toll_like_receptor_signaling_pathway																	3.10E-02										
Transcriptional_misregulation_in_cancer	4.70E-02																4.10E-02										
Tuberculosis																									4.70E-02		
Ubiquitin_mediated_proteolysis	2.50E-02														4.30E-03												
Valine_leucine_and_isoleucine_degradation											2.00E-02	1.00E-02															
Vasopressin_regulated_water_reabsorption																								1.80E-02			
VEGF_signaling_pathway														2.40E-02													
Vibrio_cholerae_infection																								3.00E-02			

P values of each pathway with p value < 0.05 in every single cancer type are shown.

table S17. Pathway enrichment analysis of the top 200 genes showing negative correlated with sense mutation loads.

Pathway	AML	ACC	BLCA	LGG	BRCA	COAD	ESCA	GBM	HNSC	KICH	KIRC	KIRP	LIHC	LUAD	LUSC	DLBC	OV	PAAD	PRAD	SARC	SKCM	STAD	TGCT	THCA	UCS	UCEC	UVM
ABC_transporters			1.30E-02																								
Adherens_junction																						2.10E-02					
Adipocytokine_signaling_pathway																			3.20E-02								
Adrenergic_signaling_in_cardiomyocytes				3.60E-02					4.90E-03									2.40E-03				1.30E-03					
Aldosterone_synthesis_and_secretion																		1.80E-03				3.00E-02					
Allograft_rejection																							1.40E-03				
Amoebiasis		2.10E-02											8.40E-04														
AMPK_signaling_pathway			1.10E-02																								
Apoptosis																							4.90E-02				
Arginine_biosynthesis				2.60E-02																							
Arrhythmogenic_right_ventricular_cardiomyopathy									4.10E-02			4.40E-02															
Autoimmune_thyroid_disease																							3.20E-02				
Axon_guidance															7.50E-03												
Bacterial_invasion_of_epithelial_cells	6.30E-04								1.00E-02																		
Basal_cell_carcinoma							1.30E-03																				
Basal_transcription_factors																3.60E-02											
Bile_secretion																					2.70E-02						
Biosynthesis_of_amino_acids	3.20E-03			1.40E-02																							
Calcium_signaling_pathway											1.20E-05	7.90E-04						5.80E-03									
cAMP_signaling_pathway				1.20E-02																		2.50E-02					
Carbon_metabolism	1.90E-02			2.90E-03																							
Cardiac_muscle_contraction									8.70E-03																		
Cell_adhesion_molecules																							1.00E-04				
cGMP_PKG_signaling_pathway											4.50E-05	4.80E-04	3.10E-02					9.50E-06				8.30E-06					
Chagas_disease																							8.60E-06				
Chemokine_signaling_pathway									1.50E-02														3.20E-05				
Cholinergic_synapse																		5.70E-03									
Circadian_entrainment				6.70E-03																							
Cytokine_cytokine_receptor_interaction													3.40E-02										1.20E-11				
D_Glutamine_and_D_glutamate_metabolism				4.90E-02																							
Dilated_cardiomyopathy															4.80E-02												
Dopaminergic_synapse																		5.00E-02									
Dorso_ventral_axis_formation												3.60E-02															
Drug_metabolism__cytochrome_P450		3.00E-02																									
ECM_receptor_interaction													3.30E-05		1.80E-04												
Endocytosis	2.50E-03							1.60E-03																			
Endometrial_cancer									1.90E-02																		
ErbB_signaling_pathway			3.90E-04																								
Estrogen_signaling_pathway																		2.60E-02									
Fc_epsilon_RI_signaling_pathway			4.00E-02																								
Fc_gamma_R_mediated_phagocytosis	2.70E-02																										
Focal_adhesion					3.10E-02				2.40E-02		1.90E-02		5.40E-05		3.60E-03												
GABAergic_synapse									1.30E-02																		
Glucagon_signaling_pathway				3.60E-02																							
Glutamatergic_synapse																		6.20E-03									
Glutathione_metabolism																			1.40E-02								
Glycine_serine_and_threonine_metabolism				1.30E-02																							
Glycosphingolipid_biosynthesis__lacto_and_neolacto_series									3.20E-02				3.00E-02														
Graft_versus_host_disease																							9.30E-03				
Hedgehog_signaling_pathway					2.20E-02																						
Hematopoietic_cell_lineage																							1.30E-04				
Hepatitis_B			5.40E-03																								
Herpes_simplex_infection																							3.50E-02				
HTLV_I_infection																							2.00E-02				
Huntington_disease								4.30E-03																			
Hypertrophic_cardiomyopathy															4.00E-02												
Inflammatory_bowel_disease																							1.00E-02				
Influenza_A			3.10E-03																				8.30E-03				
Intestinal_immune_network_for_IgA_production																							3.40E-03				
Jak_STAT_signaling_pathway	4.70E-02																			2.00E-02			4.40E-02				
Leukocyte_transendothelial_migration											3.30E-02																
Long_term_potentiation			3.70E-02	5.00E-02																							
Malaria																							2.70E-02				
MAPK_signaling_pathway			2.30E-02																								
Measles																							6.20E-05				
Melanoma															3.10E-02												
Metabolic_pathways				7.70E-03				3.10E-02									2.00E-02										
Metabolism_of_xenobiotics_by_cytochrome_P450																	4.00E-02										
Morphine_addiction									1.70E-02		1.40E-02	3.30E-03															
mRNA_surveillance_pathway														2.80E-03		2.20E-02											
Mucin_type_O_Glycan_biosynthesis									4.40E-02																		
Natural_killer_cell_mediated_cytotoxicity	2.50E-02																						2.00E-04				
NF_kappa_B_signaling_pathway																							1.40E-04				
Nicotinate_and_nicotinamide_metabolism									3.90E-02																		
NOD_like_receptor_signaling_pathway																							7.70E-04				
Notch_signaling_pathway												1.90E-03															
Oocyte_meiosis					1.40E-02													3.40E-02									
Osteoclast_differentiation	3.30E-02																						1.70E-03				
Other_types_of_O_glycan_biosynthesis							2.90E-02																				
Oxytocin_signaling_pathway									7.10E-03												1.60E-02	4.30E-02					
Pancreatic_secretion																		2.20E-02				4.30E-02					
Parkinson_disease								1.80E-02																			
Pathogenic_Escherichia_coli_infection	4.90E-03																										
Pathways_in_cancer					4.10E-04				2.60E-02		2.00E-03		2.20E-02		1.30E-02												
PI3K_Akt_signaling_pathway												3.70E-02	3.20E-03		5.70E-03												
Platelet_activation	3.20E-02										1.10E-02																
Primary_immunodeficiency																							7.70E-05				
Prion_diseases																							9.30E-03				
Progesterone_mediated_oocyte_maturation					3.80E-02																						
Protein_digestion_and_absorption		1.10E-02											3.10E-04														
Proteoglycans_in_cancer																		4.00E-02									
Proximal_tubule_bicarbonate_reclamation																					1.90E-02						
Purine_metabolism												1.30E-02															
Rap1_signaling_pathway											2.00E-02	8.60E-03															
Ras_signaling_pathway											2.10E-03																
Regulation_of_actin_cytoskeleton											5.70E-03																
Renin_secretion												5.50E-03						7.50E-04									
Retrograde_endocannabinoid_signaling				8.70E-03														2.80E-02									
Rheumatoid_arthritis																							1.10E-03				
Ribosome																	1.30E-02										
Salivary_secretion																		2.30E-03				5.70E-03					
Salmonella_infection	1.50E-05																						2.40E-02				
Serotonergic_synapse																		5.70E-03									
Shigellosis	1.70E-03																										
Small_cell_lung_cancer													1.20E-02		4.90E-02												
Staphylococcus_aureus_infection	3.70E-02																						5.60E-03				
Systemic_lupus_erythematosus																							2.00E-03				
T_cell_receptor_signaling_pathway																							9.10E-07				
Thyroid_hormone_signaling_pathway			1.60E-03																		2.20E-02						
Thyroid_hormone_synthesis																					4.10E-03						
Tight_junction																						5.60E-03					
TNF_signaling_pathway																							4.90E-04				
Toll_like_receptor_signaling_pathway																							1.30E-02				
Toxoplasmosis															5.50E-03												
Transcriptional_misregulation_in_cancer																				3.30E-02							
Tuberculosis	1.00E-02																										
Type_I_diabetes_mellitus																							1.80E-02				
Vascular_smooth_muscle_contraction									4.00E-02		3.40E-02	1.00E-02						7.20E-03			2.50E-02	1.70E-02					
Viral_myocarditis																							9.10E-04				
Wnt_signaling_pathway				3.00E-02			6.70E-03														4.00E-02						

P values of each pathway with p value < 0.05 in every single cancer type are shown.
