# Supplementary material for: Pan-cancer analysis reveals synergistic effects of CDK4/6i and PARPi combination treatment in RB-proficient and RB-deficient breast cancer cells
Source: Cell Death Dis. 2020 Apr 6;11(4):219. doi: 10.1038/s41419-020-2408-1 (PMC7136254; doi:10.1038/s41419-020-2408-1)
Supplement: Supplementary file 12 — Table S18 [file 41419_2020_2408_MOESM12_ESM.pdf]

table S18. Members of cell cycle pathway.

Symbol	Gene ID	Symbol	Gene ID	Symbol	Gene ID	Symbol	Gene ID
ABL1	25	CDC25B	994	FZR1	51343	RB1	5925
ANAPC1	64682	CDC25C	995	GADD45A	1647	RBL1	5933
ANAPC10	10393	CDC26	246184	GADD45B	4616	RBL2	5934
ANAPC11	51529	CDC27	996	GADD45G	10912	RBX1	9978
ANAPC13	25847	CDC45	8318	GSK3B	2932	SFN	2810
ANAPC2	29882	CDC6	990	HDAC1	3065	SKP1	6500
ANAPC4	29945	CDC7	8317	HDAC2	3066	SKP2	6502
ANAPC5	51433	CDK1	983	MAD1L1	8379	SMAD2	4087
ANAPC7	51434	CDK2	1017	MAD2L1	4085	SMAD3	4088
ATM	472	CDK4	1019	MAD2L2	10459	SMAD4	4089
ATR	545	CDK6	1021	MCM2	4171	SMC1A	8243
BUB1	699	CDK7	1022	MCM3	4172	SMC1B	27127
BUB1B	701	CDKN1A	1026	MCM4	4173	SMC3	9126
BUB3	9184	CDKN1B	1027	MCM5	4174	STAG1	10274
CCNA1	8900	CDKN1C	1028	MCM6	4175	STAG2	10735
CCNA2	890	CDKN2A	1029	MCM7	4176	TFDP1	7027
CCNB1	891	CDKN2B	1030	MDM2	4193	TFDP2	7029
CCNB2	9133	CDKN2C	1031	MYC	4609	TGFB1	7040
CCNB3	85417	CDKN2D	1032	ORC1	4998	TGFB2	7042
CCND1	595	CHEK1	1111	ORC2	4999	TGFB3	7043
CCND2	894	CHEK2	11200	ORC3	23595	TP53	7157
CCND3	896	CREBBP	1387	ORC4	5000	TTK	7272
CCNE1	898	CUL1	8454	ORC5	5001	WEE1	7465
CCNE2	9134	DBF4	10926	ORC6	23594	WEE2	494551
CCNH	902	E2F1	1869	PCNA	5111	YWHAB	7529
CDC14A	8556	E2F2	1870	PKMYT1	9088	YWHAE	7531
CDC14B	8555	E2F3	1871	PLK1	5347	YWHAG	7532
CDC16	8881	E2F4	1874	PRKDC	5591	YWHAH	7533
CDC20	991	E2F5	1875	PTTG1	9232	YWHAQ	10971
CDC23	8697	EP300	2033	PTTG2	10744	YWHAZ	7534
CDC25A	993	ESPL1	9700	RAD21	5885	ZBTB17	7709

According to DAVID Functional Annotation Tool, 124 members of cell cycle pathway are shown.
