# Supplementary material for: Pan-cancer analysis reveals synergistic effects of CDK4/6i and PARPi combination treatment in RB-proficient and RB-deficient breast cancer cells
Source: Cell Death Dis. 2020 Apr 6;11(4):219. doi: 10.1038/s41419-020-2408-1 (PMC7136254; doi:10.1038/s41419-020-2408-1)
Supplement: Supplementary file 13 — Supplementary Table Legend [file 41419_2020_2408_MOESM13_ESM.docx]

**SUPPLEMENTARY TABLES LEGENDS**

**table S1. Summary of TCGA cancer types, patients, and all mutation loads surveyed in this study.** For each data type and each cancer type, the numbers of patients and mutations available in the analysis are shown. Base-substitution mutations include A>C, A>G, A>T, C>A, C>G and C>T substitution. INS and DEL represent insertion and deletion mutations, respectively.

**table S2. Summary of cancer types, patients, and missense mutation loads.** For each data type and each cancer type, the numbers of patients and mutations available in the analysis are shown.

**table S3. Summary of cancer types, patients, and sense mutation loads.** For each data type and each cancer type, the numbers of patients and mutations available in the analysis are shown.

**table S4. Genes showing positive correlation with mutation load in at least 5 types of cancer.** The rank numbers of each positive gene in different cancer types are shown.

**table S5. Genes showing negative correlation with mutation load in at least 5 types of cancer.** The rank numbers of each negative gene in different cancer types are shown.

**table S6. Pathway enrichment analysis of the top 400 genes showing positive correlation with all mutation loads in every single type of cancers.** P values of each pathway with p value < 0.05 in every single cancer type are shown.

**table S7. Pathway enrichment analysis of the top 400 genes showing negative correlation with all mutation loads.** P values of each pathway with p value < 0.05 in every single cancer type are shown.

**table S8. Pathway enrichment analysis of the top 200 genes showing positive correlation with all mutation loads.** P values of each pathway with p value < 0.05 in every single cancer type are shown.

**table S9. Pathway enrichment analysis of the top 200 genes showing negative correlation with all mutation loads.** P values of each pathway with p value < 0.05 in every single cancer type are shown.

**table S10. Pathway enrichment analysis of the top 400 genes showing positive correlated with missense mutation loads.** P values of each pathway with p value < 0.05 in every single cancer type are shown.

**table S11. Pathway enrichment analysis of the top 400 genes showing negative correlated with missense mutation loads.** P values of each pathway with p value < 0.05 in every single cancer type are shown.

**table S12. Pathway enrichment analysis of the top 200 genes showing positive correlated with missense mutation loads.** P values of each pathway with p value < 0.05 in every single cancer type are shown.

**table S13. Pathway enrichment analysis of the top 200 genes showing negative correlated with missense mutation loads.** P values of each pathway with p value < 0.05 in every single cancer type are shown.

**table S14. Pathway enrichment analysis of the top 400 genes showing positive correlated with sense mutation loads.** P values of each pathway with p value < 0.05 in every single cancer type are shown.

**table S15. Pathway enrichment analysis of the top 400 genes showing negative correlated with sense mutation loads.** P values of each pathway with p value < 0.05 in every single cancer type are shown.

**table S16. Pathway enrichment analysis of the top 200 genes showing positive correlated with sense mutation loads.** P values of each pathway with p value < 0.05 in every single cancer type are shown.

**table S17. Pathway enrichment analysis of the top 200 genes showing negative correlated with sense mutation loads.** P values of each pathway with p value < 0.05 in every single cancer type are shown.

**table S18. Members of cell cycle pathway.** According to DAVID Functional Annotation Tool, 124 members of cell cycle pathway are shown.
